# Vertically transmitted microbiome protects eggs from fungal infection and egg failure

**DOI:** 10.1186/s42523-021-00104-5

**Published:** 2021-06-16

**Authors:** M. E. Bunker, G. Elliott, H. Heyer-Gray, M. O. Martin, A. E. Arnold, S. L. Weiss

**Affiliations:** 1 Department of Biology, University of Puget Sound, Tacoma, WA USA; 2 School of Plant Sciences and Department of Ecology and Evolutionary Biology, University of Arizona, AZ Tucson, USA

**Keywords:** Antifungal bacteria, Cloaca, Eggshell, Fitness, Illumina, Lizard, Microbiome, Scanning electron microscopy, *Sceloporus*

## Abstract

**Background:**

Beneficial microbes can be vertically transmitted from mother to offspring in many organisms. In oviparous animals, bacterial transfer to eggs may improve egg success by inhibiting fungal attachment and infection from pathogenic microbes in the nest environment. Vertical transfer of these egg-protective bacteria may be facilitated through behavioral mechanisms such as egg-tending, but many species do not provide parental care. Thus, an important mechanism of vertical transfer may be the passage of the egg through the maternal cloaca during oviposition itself. In this study, we examined how oviposition affects eggshell microbial communities, fungal attachment, hatch success, and offspring phenotype in the striped plateau lizard, *Sceloporus virgatus*, a species with no post-oviposition parental care.

**Results:**

Relative to dissected eggs that did not pass through the cloaca, oviposited eggs had more bacteria and fewer fungal hyphae when examined with a scanning electron microscope. Using high throughput Illumina sequencing, we also found a difference in the bacterial communities of eggshells that did and did not pass through the cloaca, and the diversity of eggshell communities tended to correlate with maternal cloacal diversity only for oviposited eggs, and not for dissected eggs, indicating that vertical transmission of microbes is occurring. Further, we found that oviposited eggs had greater hatch success and led to larger offspring than those that were dissected.

**Conclusions:**

Overall, our results indicate that female *S. virgatus* lizards transfer beneficial microbes from their cloaca onto their eggs during oviposition, and that these microbes reduce fungal colonization and infection of eggs during incubation and increase female fitness. Cloacal transfer of egg-protective bacteria may be common among oviparous species, and may be especially advantageous to species that lack parental care.

**Supplementary Information:**

The online version contains supplementary material available at 10.1186/s42523-021-00104-5.

## Background

Microbiomes associated with animals typically comprise diverse communities that live in and on their animal hosts [1, 2]. These microbes often confer benefits to their hosts, including nutritional supplementation and protection from pathogens, with the composition of the microbial community typically influenced by host behavior, health, and genetics [3, 4]. As studies of the microbiome expand, it is increasingly evident that microbiomes may play a key role in influencing the behavior and evolution of their animal hosts [2, 5, 6]. By impacting reproductive behavior or outcomes, microbiomes are increasingly appreciated as driving diversification and influencing speciation rates in diverse lineages [6].

Bacterial communities associated with hosts can affect individual fitness by diverse means of manipulating reproductive success: for example, they can influence mate choice, affect offspring survival, and behave as heritable phenotypes [4, 7]. In mammals, certain microbes are vertically transmitted through a variety of mechanisms: trans-ovarian and trans-uterine exchange, maternal-offspring contact during parturition, and via nursing and other post-natal interactions [8, 9]. However, both the mechanisms and consequences of vertical microbe transmission in oviparous animals, particularly reptiles, have received less attention [10].

Recent evidence suggests that egg-laying animals can pass microbes to their offspring throughout the developmental process [11–14]. The eggshell creates a barrier for microbial transmission to the embryo, but also an opportunity for host-microbe interactions via microbial protection of the egg from environmental pathogens. Infection from pathogens can lead to egg failure and decreased fitness, and host microbes can prevent those infections, increase hatch rates, and confer valuable benefits later in life [15–17]. Recent studies of house geckos and eastern fence lizards have shown that *in ovo* bacteria are present, likely transferred from the gut or ovarian microbiome during egg development [13, 18]. Studies of endangered sea turtles indicate that while infection with pathogenic strains of the fungus *Fusarium* is common in nests, so too is the presence of bacteria with antifungal properties, which could be deposited on eggs from the mother’s cloaca [17]. After oviposition, several species of birds have been observed transferring beneficial bacteria to their eggs through brood patches and preen oil, as well as other parental care behaviors [14, 19]. Birds also have the opportunity to transfer microbes directly to hatchlings by sharing their nest and food with their offspring [14, 19]. For species without parental care, like the majority of reptiles, passing of microbes must take place during egg development and oviposition.

*Sceloporus virgatus,* the striped plateau lizard, does not provide care for eggs after oviposition. This species is found primarily in oak-juniper scrub habitats of Arizona, New Mexico, and northern Mexico, and their reproduction is tied to the rain cycle. At the onset of the summer monsoon season (typically early July), gravid *S. virgatus* females bury eggs in soil nests (~ 6 cm deep [20]) and provide no further care. Throughout the ~ 8 week incubation period, eggs are exposed to soil-borne microbes at a time of warm, moist conditions, excellent for fungal growth. Given this challenge, selection may act to favor those females that provide antifungal protection to their eggs [15]. We hypothesize that, during oviposition, *S. virgatus* mothers transfer beneficial microbes from their cloaca onto the surface of their eggs, and that these microbes have antifungal properties that reduce infection by pathogenic fungi found in the nest environment [21].

Here we examine this hypothesis by comparing eggs that did and did not have opportunity to be inoculated with antifungal microbes from the mother’s cloaca; eggs that did not contact the cloaca were removed by dissection. We used scanning electron microscopy (SEM) and high-throughput amplicon sequencing to compare microbial loads and community structure of oviposited and dissected eggshells to determine the influence of the maternal microbiome. To investigate the protective function of the microbiome, we further examined the effect of pathogen exposure to these two groups of eggs on fungal attachment rates, hatch success, and hatchling phenotype. We predicted that relative to oviposited eggs, dissected eggs would have (i) a lower bacterial load and higher fungal load, (ii) a different bacterial community structure, (iii) a weaker relationship to maternal cloacal microbial communities, (iv) higher fungal attachment rates, and (v) lower hatch success and resulting hatchling condition when challenged with potential fungal pathogens.

## Methods

### Sample collection

Gravid *Sceloporus virgatus* females were collected using a loop of fishing line tied to a variable length fishing pole from areas surrounding the American Museum of Natural History’s Southwestern Research Station (SWRS) in Cochise County, Arizona, USA between June 28 and July 1, 2019. Only females with > 62 mm snout-vent length (SVL) were used for this study. Lizard cloacae were swabbed in the field immediately after capture by gently inserting a sterile swab (BD ESwab™) into the cloaca and slowly rotating it. Microbes were eluted from the swab into an Amies solution and stored at − 80 °C until DNA extraction and amplicon sequencing.

Lizards were kept in large outdoor enclosures at SWRS until July 2, 2019 when they were shipped overnight in individual plastic containers on ice packs to the Weiss lab at the University of Puget Sound. On the day of arrival, females were swabbed again for comparison to the field swab and randomly assigned to treatment groups to generate two categories of eggs: dissected (*n* = 135 eggs) and oviposited (*n* = 103 eggs). Females providing dissected eggs (*n* = 12 females) were euthanized with a two-step procedure using buffered MS-222 [22]. Eggs were surgically removed from the oviduct with sterilized instruments. Females providing oviposited eggs (*n* = 12 females) were injected with 2 USP units of oxytocin. Eggs were handled with sterile gloves and instruments. All eggs were weighed and assigned haphazardly to one of five experimental groups across three experiments, as described below.

### Experiment 1: describing microbial communities

The first experiment examined microbial load, community diversity, and community structure of eggs at Day 0 and Day 25 of incubation in soil. Eggs to be examined on Day 25 (approximately halfway through lab incubation) were individually buried in 50 ml cups of autoclaved vermiculite inoculated with a 1 g:10 ml soil:water slurry, made from soil collected at SWRS in order to expose eggs to natural environmental microbes (0.8 ml slurry/g vermiculite). Egg cups were covered in parafilm and incubated at 28 °C. After the appropriate time period, a small incision was made with sterile scissors at the tip of each egg, and the contents were expelled. A small portion (~ 15 mm^2^) of shell was saved at − 80 °C for amplicon sequencing, and the remaining shell was cut in half and prepared for imaging.

#### SEM imaging

Each eggshell piece was placed in 2 mL of fixative (2% paraformaldehyde, 2% gluteraldehyde, 2% DMSO, and 1x phosphate buffer solution (PBS)) for 2 h, then in 1x PBS for 48 h. Eggshells were dried with a standard SEM specimen drying procedure, mounted on individual stubs, and placed in an airtight container with desiccant overnight. Mounted specimens were sputter-coated with gold palladium and observed with the scanning electron microscope (SEM; Hitachi S3400N Variable Pressure Scanning Electron Microscope).

For Day 0 eggshells (dissected: *n* = 12, oviposited: *n* = 12), the average density of bacteria (the number of bacteria within the field of view) on the shell was quantified by scanning 30 randomly selected locations at 2.5 k x magnification per egg. At this stage, no fungi were identified on the eggshells. Day 25 eggshells (dissected: *n* = 11, oviposited: *n* = 9) were examined by scanning 15 randomly selected locations at 2.5 k x magnification to quantify the density of bacteria and fungal hyphae on the shell. Fewer locations were selected for imaging at Day 25 because bacteria were more abundant than on Day 0 and required less intensive scanning to find them. All observed bacteria were rod-shaped. We used two sample t-tests to determine whether dissected and oviposited eggshells differed in mean density of bacteria on Day 0 and on Day 25, as well as mean fungal hyphae on Day 25. Data were log-transformed as needed to meet test assumptions.

#### DNA extraction for amplicon sequencing

Total genomic DNA was extracted from eggshell pieces and cloacal swabs via the Qiagen DNEasy©Blood and Tissue Kit (Qiagen, Inc). For the cloacal swab samples we used the manufacturer’s protocol for Purification of Total DNA from Animal Blood or Cells, with the optional pre-treatment for Gram-positive bacteria (lysis buffer incubation). An extraction blank was included in each extraction and processed as below for library preparation and sequencing. Eggshells were rinsed with sterile PBS, then a ~ 2 × 2 mm square of shell was prepared for DNA extraction. Samples were incubated in the optional lysis buffer for 30 min at 37 °C. After incubation, Buffer AL and proteinase K were added to the tubes, and the shells were beat with sterile tungsten beads using a TissueLyser at 30 hz for 2 × 1 min. The samples were incubated at 56 °C for 90 min, while shaking at 500 RPM. From here, the extraction was completed according to the Purification of Total DNA from Animal Blood or Cells protocol, beginning at Step 3 (addition of pure ethanol). Extraction blanks for eggshell extractions included 200 uL of sterile PBS, as the sterile forceps and scissors that were used to subsample the shells were dipped into the PBS prior to the extraction procedure, as well as extraction kit blanks as above. DNA in all samples and blanks was quantified via Qubit prior to processing.

#### Illumina library prep

A two-step polymerase chain reaction (PCR) process was used to amplify the 16 s rRNA gene V4 region in each sample [23, 24]. PCR1 utilized 515F/806R primer pairs. Six variations of the primers were pooled, each with a 0–5 base pair (bp) shift, linked to locus-specific sequences and a consensus sequence with a 2 bp linker [24]. PCR1 had a 15 uL reaction volume, containing 7.5 uL Phusion Flash High Fidelity Master Mix (ThermoFisher, Waltham, MA), 0.15 uL of the forward and reverse primers, 0.75 uL molecular grade Bovine Serum Albumin (BSA, 20 mg/mL), 5.45 uL purified water, and 1 uL template DNA. The thermal cycling protocol was: initial denaturation at 98 °C for 10 s; 28 cycles of denaturation at 98 °C for 1 s, annealing at 57 °C for 5 s, and extension at 72 °C for 20 s; then one final extension at 72 °C for 60 s [24]. PCR products were visualized on a 2% agarose gel using 10x Sybr Green (Molecular Probes, Invitrogen; Carlsbad, CA). All iterations of PCR1 included a positive control, containing *Serratia* genomic DNA, and a negative control, containing only purified water and no DNA template. A mock community for bacteria from BEI Resources (ATCC, Manassas, VA) was processed in parallel to assess potential primer bias and evaluate the relationship of observed and expected read number (see [24]).

PCR1 was performed in triplicate, and all replicates were pooled for PCR2, which extended the amplicons with the sample-specific barcodes. Samples (including positive controls) that showed strong bands in at least two of three PCR1 replicates were diluted 1:4 in purified water before being used as template DNA in PCR2. Samples with faint or no bands in at least two of three PCR1 replicates were used directly for DNA template in PCR2. In addition to pooling replicates of the negative controls, negative controls from different PCR1 runs were further pooled to minimize the number of samples sent for sequencing. Because of this, some iterations of PCR2 contained a pooled PCR1 negative control, and some contained a new PCR2 negative control with purified water instead of DNA template.

PCR2 had a reaction volume of 20 uL containing 10 uL Phusion Flash High Fidelity Master Mix, 0.24 uL BSA, 8.01 uL of purified water, 0.75 uL of unique barcoded primer pairs (supplied by IBest Genomics Core, University of Idaho; see [24]), and 1 uL of template DNA. The thermal cycling protocol was the same as the protocol for PCR1, except with an annealing temperature of 51 °C. PCR2 was only run for 8 cycles (for a total of 36 cycles total). PCR2 products were visualized on a 2% agarose gel with 10x Sybr Green. We confirmed that each sample had undergone a band shift compared to PCR1, indicative of attachment of barcode primers. Each sample band was given a score from 0 to 5 based on band intensity; these scores were then used to determine pool volume for each sample. Samples were shipped to IBest Genomic Core for purification and sequencing on the Illumina MiSeq platform. All negative controls and mock communities were included in the sequencing process, but positive controls were not.

#### Illumina raw data processing and analysis

Sequences were received demultiplexed, with adapters and primers removed. Quality analysis for each sample was performed using FastQC [25] and those results were consolidated using MulitQC [26]. Mean quality scores and length distribution for the whole dataset was manually inspected and used to determine a cutoff length of 265 bp for forward reads and 185 bp for reverse reads. Samples were then processed in R v3.1.6 via the DADA2 [27] pipeline. Samples were trimmed as described above and filtered with a max expected error of 2. An average of 78% of reads were kept in all experimental samples after processing.

Taxonomic classification of amplicon sequence variants (ASVs) was performed through the assignTaxonomy function, using the Silva database [28], release 132. Potential contaminants were removed with the Decontam package [29], using the “prevalence” method with a threshold of 0.1. Control samples (*n* = 23), including experimental controls, extraction blanks, and PCR negatives were used for comparison. Any ASV that had fewer than 27 reads across all samples was discarded, based on the inspection of the mock community, and read numbers were log transformed.

Once samples had been processed, the phyloseq package [30] was used to organize and store data of different types for analysis. Shannon diversity index values were calculated using phyloseq (estimate_richness function). We assessed whether the Shannon diversity and richness of the cloacal microbiome differed before and after lizards were shipped to the lab using paired t-tests, and between females that would go on to be either dissected or induced to oviposit on the day of egg acquisition using two sample t-tests. We assessed whether diversity and richness of eggshell microbiomes differed based on egg type (dissected and oviposited) at Day 0 and Day 25 via two-sample t-tests when possible and Wilcoxon tests if data did not meet parametric assumptions. Response variables were log transformed when needed to meet the assumption of equal variance. We looked for a relationship between maternal cloacal diversity and Day 0 eggshell diversity using Pearson’s correlation tests; only animals that had both a Day 0 eggshell and an available cloacal swab were included in this analysis.

Pairwise distances between samples were calculated by the vegan package [31] using Bray-Curtis distances, and these distances were then used to generate non-metric multidimensional scaling (NMDS) plots. Dispersion between explanatory variables of interest was first tested with the betadisper function from the vegan package and a PERMANOVA test was performed to compare community composition (Adonis function from the vegan package). All plots were made with the GGplot2 package [32].

### Experiment 2: fungal attachment assays

To test whether the eggshell microbiome inhibits fungal attachment, fungal attachment assays were performed on dissected and oviposited eggshells after 9 d of incubation in sterile vermiculite mixed with sterile water (0.8 mL/g). Eggshell halves were put into 1 ml suspensions of *Aspergillus protuberus* (1.04 × 10^6^ hyphae/mL) or *Neocosmospora rubicola* (2.48 × 10^6^ hyphae/mL) and incubated at room temperature for 48 h. These fungi had been previously cultured from soil samples taken from areas where natural *S. virgatus* nest burrows would be constructed, and are known to be pathogenic in other systems [33, 34].

After incubation, shells were rinsed with deionized water and prepared for SEM as described above. The density of fungi attached to the shell surface were quantified at 2.5 k x magnification per egg. Counts were performed on 15 random locations across each shell piece and the density of fungal hyphae on dissected and oviposited eggs were compared. Hyphal attachment of *A. protuberus* was non-normal, even following standard transformations, and was compared across egg types with a Mann-Whitney U test. Log-transformed *N. rubicola* attachment was analyzed with a two sample t-test.

### Experiment 3: hatch success

The final experiment considered hatch success of dissected and oviposited eggs that were incubated in either sterile (dissected: *n* = 42; oviposited: *n* = 31) or fungal-inoculated (dissected: *n* = 41; oviposited: *n* = 30) vermiculite. Each mother had eggs in both incubation conditions. Eggs were individually buried in 50 ml cups of sterile vermiculite moistened with 0.8 mL/g of either sterile water or fungal suspension (described below). Egg cups were covered in parafilm and incubated at 28 °C.

The fungal suspension used to challenge the *S. virgatus* eggs included 9 strains of fungi originally cultured from failed *S. virgatus* eggs with visible fungal infection (Table 1). Plates with complete lawns of each strain were flooded with a sterile 0.01% Tween solution, and a sterile spreader was used to dislodge fungal spores and hyphae into the liquid. The concentration of each fungal solution was determined with a hemocytometer and adjusted to ~ 1 X 10^8^ cells/mL of solution, and then the nine fungi were combined into a single fungal suspension.

**Table 1:** Fungal strains included in the fungal suspension used for egg incubation

Taxonomic classification	Estimated final spore concentration (spores/mL)
*Purpureocillium lilacinum*	6.25 × 10 ^4^
*Aspergillus sydowii*	5.33 × 10 ^5^
*Penicillium* sp.	3.25 × 10 ^6^
*Penicillium* sp.	1.05 × 10 ^5^
*Aspergillus insuetus*	6.13 × 10 ^5^
*Neocosmospora rubicola*	6.05 × 10 ^6^
*Penicillium canescens*	3.25 × 10 ^5^
Unknown species	3.20 × 10 ^5^
Unidentified mixed culture	1.08 × 10 ^5^

At Day 25 of incubation, eggs were unearthed, examined for viability, swabbed for culturing of bacteria and other experimental work (not included herein), and re-buried. Eggs were scored as non-viable if they were completely desiccated and/or overgrown with fungus. Beginning at Day 36 of incubation, eggs were checked daily for hatchlings. We scored hatch success of eggs, and hatch time (i.e., incubation period), body mass, and SVL of hatchlings.

The effect of egg type (dissected vs. oviposited) and incubation environment (sterile vs. fungal-inoculated) on viability at Day 25 and hatch success were examined using a generalized linear mixed-effects model (GLMM) with a binomial (logit) error distribution and mother ID as a random factor. Viability at Day 25 and hatch success were calculated in R with the cbind(x,y) function, where x was the number of non-viable or failed eggs and y was the number of viable or hatched eggs. The effects of egg type and incubation environment on hatchling phenotype were examined using linear mixed models with mother ID as a random factor. We used the lme4 package [35] to run the models and used the lmerTest package [36], as needed, to calculate *p*-values.

The above experiment followed up on a preliminary study of females collected June 27 to July 4, 2017 (minimum SVL = 58 mm). These females (*n* = 14) were kept in large outdoor enclosures until July 8, 2017 when eggs were acquired by dissection or oviposition, as described above. All eggs from the 2017 study were incubated individually in 50 ml cups at 30 °C in the presence of natural environmental microbes by moistening vermiculite with a 1 g:10 ml soil:water slurry made from soil collected at SWRS (0.8 ml slurry/g vermiculite); eggs were incubated in the Arnold lab at the University of Arizona, Tucson, AZ. As above, we scored hatch success of eggs, and hatch time, body mass, and SVL of hatchlings. Data were analyzed similarly to above with the exclusion of an incubation environment factor. This study initially included a group of females (*n* = 8) treated with a 3 d series of oral antibiotic (0.03 ml/d of compounded 2 mg/ml enrofloxacin) in an attempt to reduce and alter the cloacal microbiome; however, the treatment was largely unsuccessful (see Additional File 1) so eggs from those females are not included here.

## Results

### SEM imaging

**Fig. 1:**
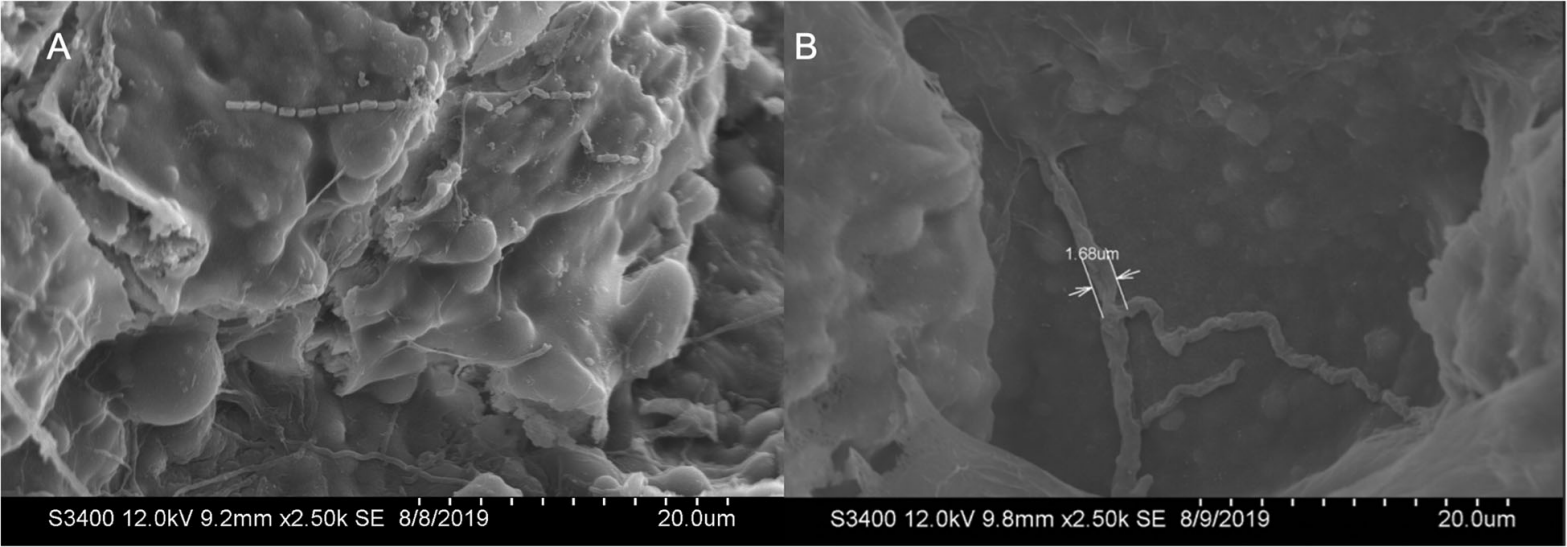
**A** Scanning electron microscope images of rod-shaped bacteria and fungal hyphae on *S. virgatus* eggshells. **B** Fungal hyphae cover the eggshell in a branched structure that spreads across the surface. Both images taken at 2.5 k magnification

**Fig. 2:**
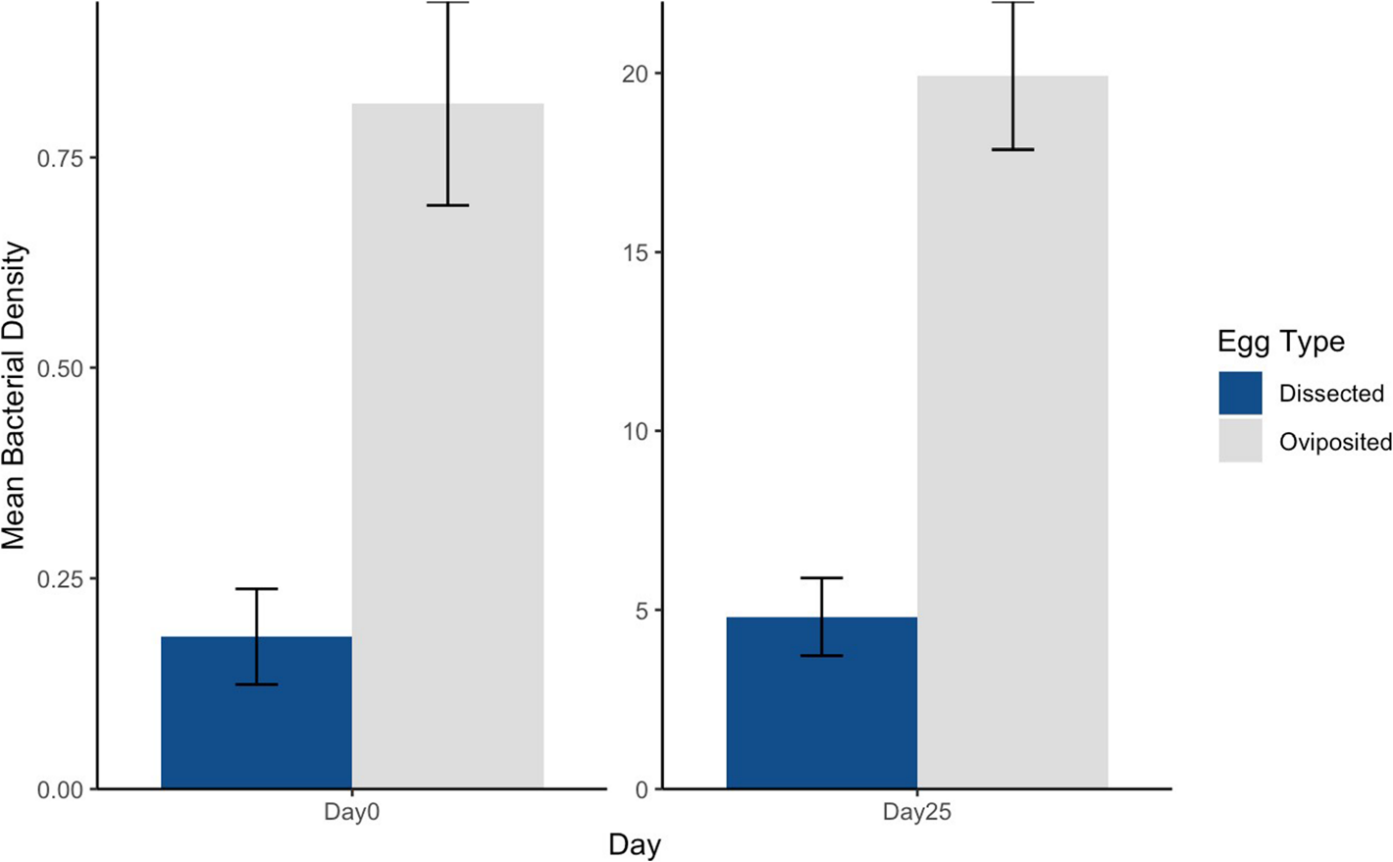
The mean (**±**SE) density of bacteria (per 1800 μm^2^) on *S. virgatus* eggshells at Day 0 and Day 25 of incubation in soil slurry. Bacteria were significantly less dense on dissected eggs than on oviposited eggs on both days (*p* < < 0.001). Counts were made via SEM by randomly selecting locations on the eggshell surface and measuring the density of bacteria at 2.5 k magnification

**Fig. 3:**
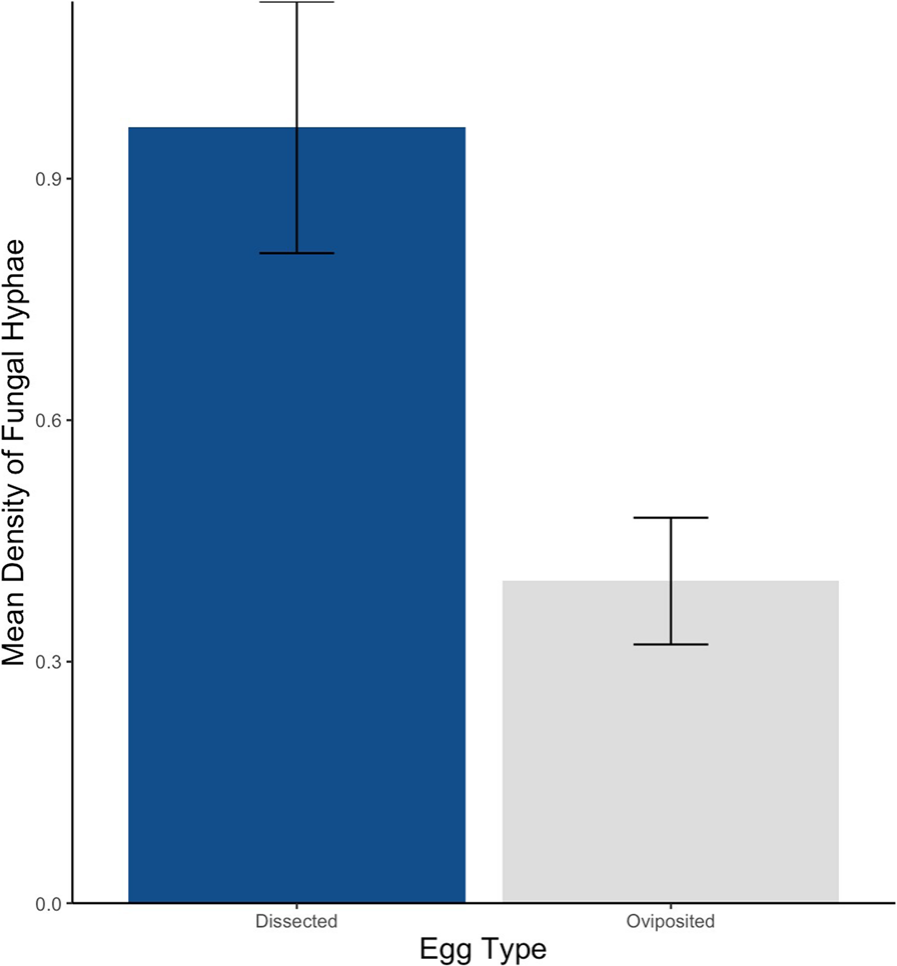
Mean (**±**SE) density (per 1800 μm^2^) of fungal hyphae found on *S. virgatus* eggshells on Day 25 of incubation. The density of hyphae was significantly greater on dissected eggs than on oviposited eggs (*p* = 0.008)

The density of bacteria observed on eggshell surfaces (Fig. 1a) differed between egg types on both Day 0 (t = − 5.81, df = 22, *p* < < 0.001) and Day 25 (t = − 6.82, df = 18, *p* < < 0.001). As predicted, the average density of eggshell bacteria was significantly lower on dissected eggs than on oviposited eggs at both time points (Fig. 2). Fungal hyphae were not observed on eggshells at Day 0, but were present at Day 25 (Fig. 1b), with significantly more hyphae on dissected eggs than on oviposited eggs (t = 3.01, df = 18, *p* = 0.008; Fig. 3).

### Cloacal microbial communities

The cloacal microbiome of gravid females did not change significantly from the time they were collected in the field in AZ to the time they were sampled in the laboratory (Table 2). On the day of egg acquisition, the cloacal microbiome of females to be dissected and females to be induced to oviposit differed in alpha diversity (t = 4.11, df = 15, *p* = 0.001) and richness (t = 3.90, df = 15, *p* = 0.001; Table 3). This pattern was unexpected as the females were randomly assigned to treatment groups. Given the pattern was for dissected females to have more diverse cloacal microbiomes, and our prediction is that the eggs from these females will have less diverse eggshell microbiomes, the difference detected here is conservative, working against our ability to find the predicted pattern. The two groups of females did not differ in cloacal microbiome dispersion (F = 0.14, df = 1,15, *p* = 0.716) or composition (F = 1.69, df = 1,15, *p* = 0.161).

**Table 2:** Effect of shipping on cloacal microbiome of *S. virgatus* females

	Test statistic	df	p
Shannon diversity	t = 1.49	16	0.156
Richness	t = 1.47	16	0.162
Community dispersion	F = 1.39	1,36	0.246
Community composition	F = 0.91	1,36	0.472

**Table 3:** Diversity metrics from laboratory swabs of *S. virgatus* female cloacae from each treatment group

	Dissected	Oviposited
Shannon diversity	4.11 ± 0.10	3.36 ± 0.15
Richness	65.78 ± 7.27	31.75 ± 4.29

Data are means ± SE

Overall, the cloacal community was dominated by *Enterobacteriaceae,* which makes up 67.95% ± 7.2% of the communities on average, on the day of egg acquisition. The next most abundant taxa was *Helicobacteraceae,* making up an average of 20.1% ± 7.2% of reads on average. *Lachnospiraceae, Bacteroidaceae,* and *Corynebacteriaceae* were all between 1 and 4% on average, and no other families made up more than 1% of the reads.

### Eggshell microbial communities

**Fig. 4:**
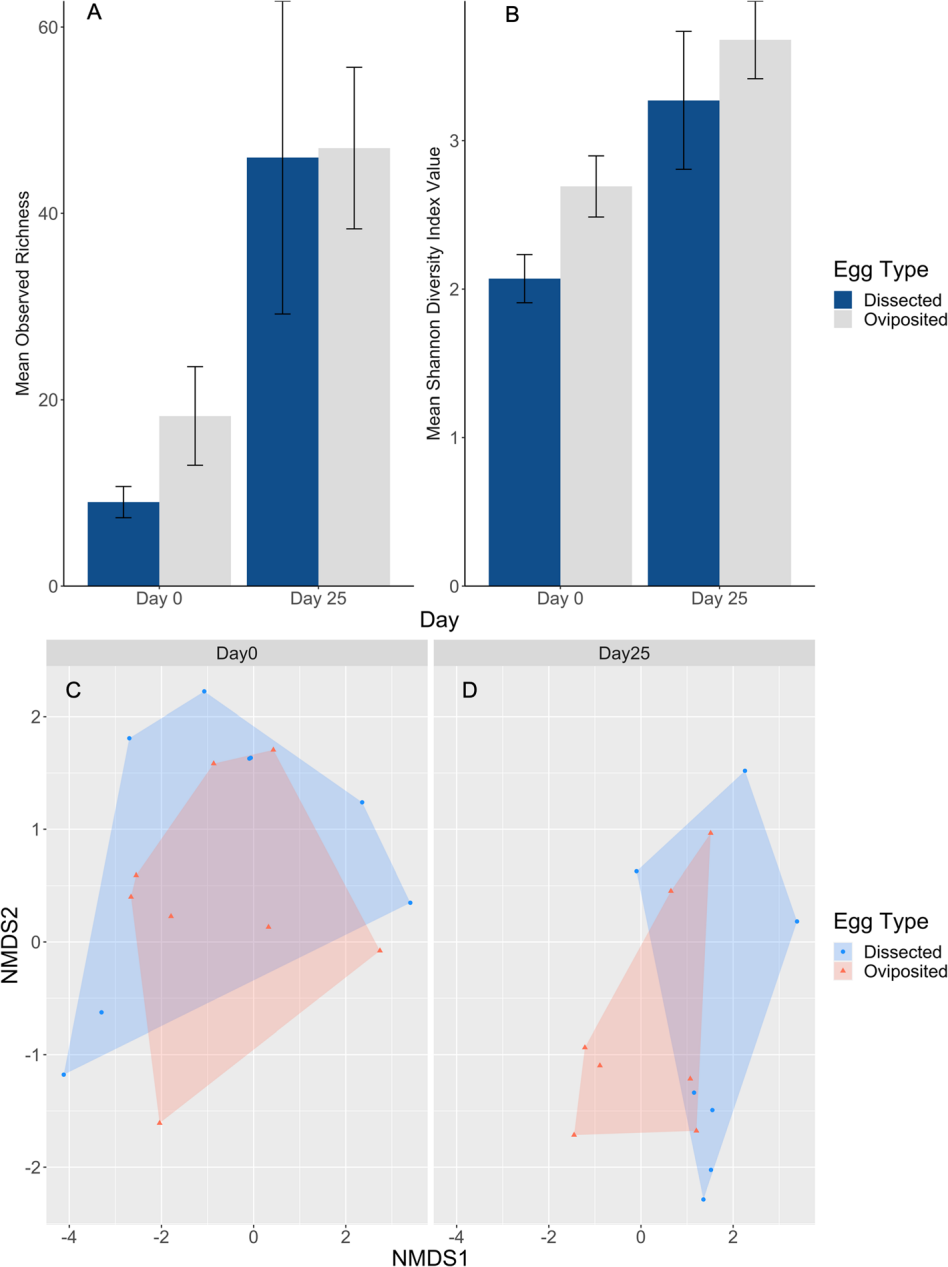
**A** Mean observed richness (**±**SE) and **B** Mean Shannon diversity index values (**±**SE) for dissected and oviposited *S. virgatus* eggs. Communities were sampled at Day 0 and Day 25 of incubation in soil slurry. Non-metric multidimensional scaling plots were created by using Bray Curtis distance to calculate pairwise distances based on community composition on **C**) Day 0 and **D**) Day 25. Three dimensions were used to calculate distances, but only the most influential two are pictured here

Microbial richness and community diversity were significantly lower on dissected eggshells than on oviposited eggshells on Day 0 (richness: t = − 2.39, df = 14, *p* = 0.031; Shannon: t = − 2.60, df = 14, *p* = 0.021; Fig. 4a-b). These differences were not observed at Day 25 of incubation (richness: t = − 0.05, df = 12, *p* = 0.959; Shannon: W = 21, *p* = 0.710).

Beta diversity was visualized through non-metric multidimensional scaling of pairwise Bray-Curtis distances with 3 dimensions to minimize stress. The most influential dimensions are visualized in Fig. 4c-d. There was no visual separation of the two egg types on Day 0 (Fig. 4c), but there is at Day 25 (Fig. 4d). The dispersion of the groups on Day 0 was not different between egg types (F = 0.23, df = 1,14, *p* = 0.642), and the samples of a given egg type were not more similar to one another than they were to samples in the other group (F = 0.75, df = 1,14, *p* = 0.951). On Day 25, the dispersion between groups was similar (F = 0.49, df = 1,12, *p* = 0.499), but the composition on the two types of eggs showed trends of separation (F = 1.40, df = 1,12, *p* = 0.067). This indicates that at Day 25 of incubation, the eggshells within each treatment group tended to be more similar to one another than they were to eggshells in the other group.

**Fig. 5:**
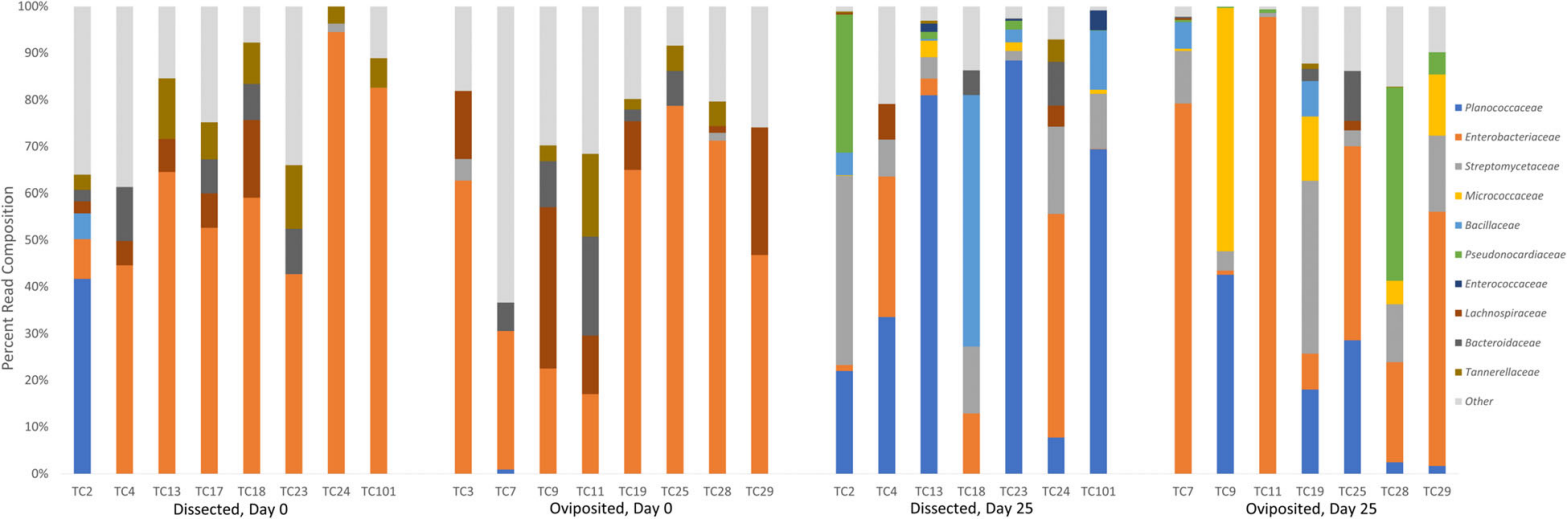
Percent composition of bacterial families in eggshell communities from eggs that were either dissected from *S. virgatus* females or oviposited. Communities were sampled immediately after egg acquisition and on Day 25 of incubation in soil slurry. Each vertical bar represents a different eggshell; labels are identification numbers for the female which provided the egg. Colored portions of the bars represent the relative abundance of the top ten most abundant taxa; the remaining taxa were combined into the “other” category. The y-axis indicates the percent composition of total reads for that sample

We also detected differences in the composition of microbial communities associated with oviposited and dissected eggshells (Fig. 5). The largest component on Day 0 were *Enterobacteriaceae*, making up an average of 56.2 ± 9.3% of all reads from dissected eggs and 49.2 ± 8.4% of total reads from oviposited eggs. The next most abundant taxa on the dissected eggs was *Tannerellaceae* (7.1 ± 1.7% of reads). For the oviposited shells, the second most abundant taxa was *Lachnospiraceae* (12.6 ± 4.5% of reads).

By Day 25 of incubation, oviposited eggs still had an average of 43.3 ± 13.7% of reads representing *Enterobacteriaceae*, with some samples having up to 97.7% of their total reads falling into that family. For dissected eggs, *Enterobacteriaceae* represented only 13.7 ± 7.0% of reads, and the highest percentage of *Enterobacteriaceae* for any given dissected egg was only 47.9%. Instead, the most prominent family for those samples was *Planococcaceae* (43.2 ± 13.7% of reads; maximum of 88.4%). Only one dissected egg shell contained *Planococcaceae* on Day 0 (41.7% of reads).

### Cloacal swab and eggshell community comparison

**Fig. 6:**
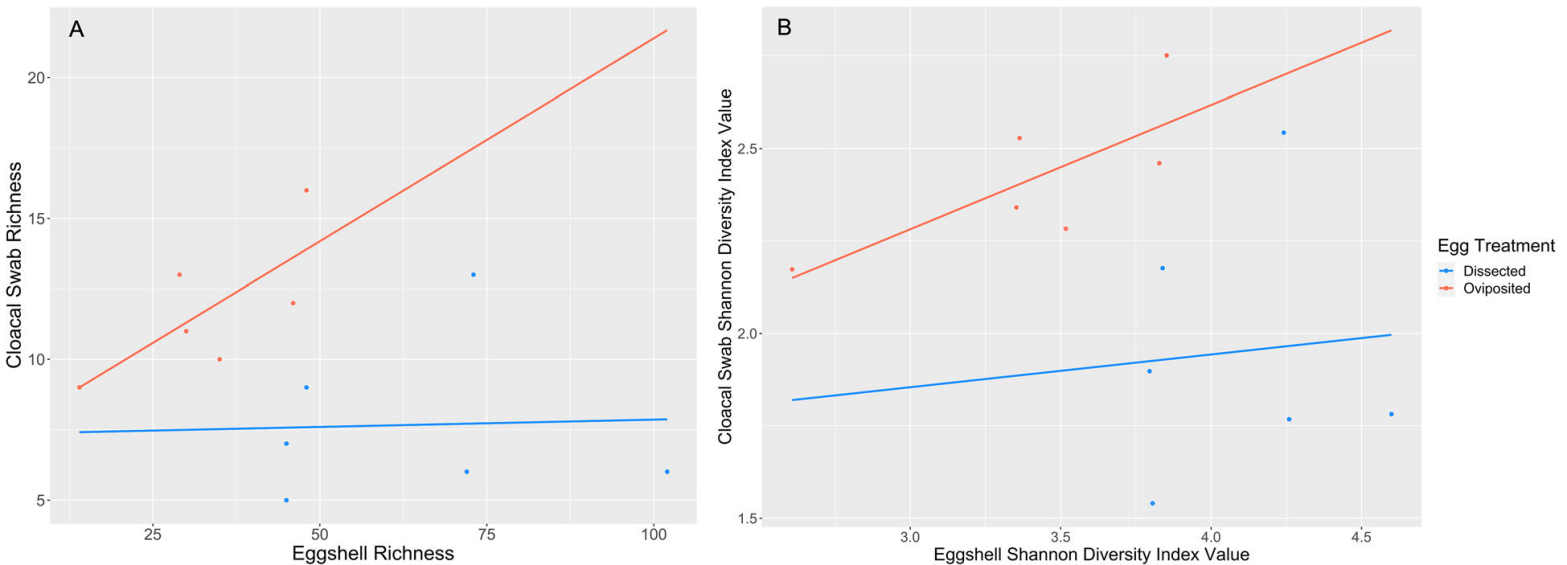
Correlation of **A**) observed richness and **B**) Shannon diversity index of *S. virgatus* eggshell bacteria relative to that of the mother’s cloaca sampled on the same day. Correlations are not significant for dissected eggs (*p* = 0.940, *p* = 0.877) but are nearly so for oviposited eggs (*p* = 0.102, *p* = 0.087)

We compared the microbiome of maternal cloacae to that of eggshells at Day 0, predicting a positive relationship for oviposited eggs and no relationship for dissected eggs. For oviposited eggs, there were trends for positive relationships between cloacal and eggshell richness (r = 0.73, df = 4, *p* = 0.102, Fig. 6a) and alpha diversity (r = 0.75, df = 4, *p* = 0.089, Fig. 6b). These trends were not present for dissected eggs (richness: r = 0.04, df = 4, *p* = 0.940, Fig. 6a; alpha diversity: r = 0.08, df = 4, *p* = 0.877, Fig. 6b).

### Fungal attachment

**Fig. 7:**
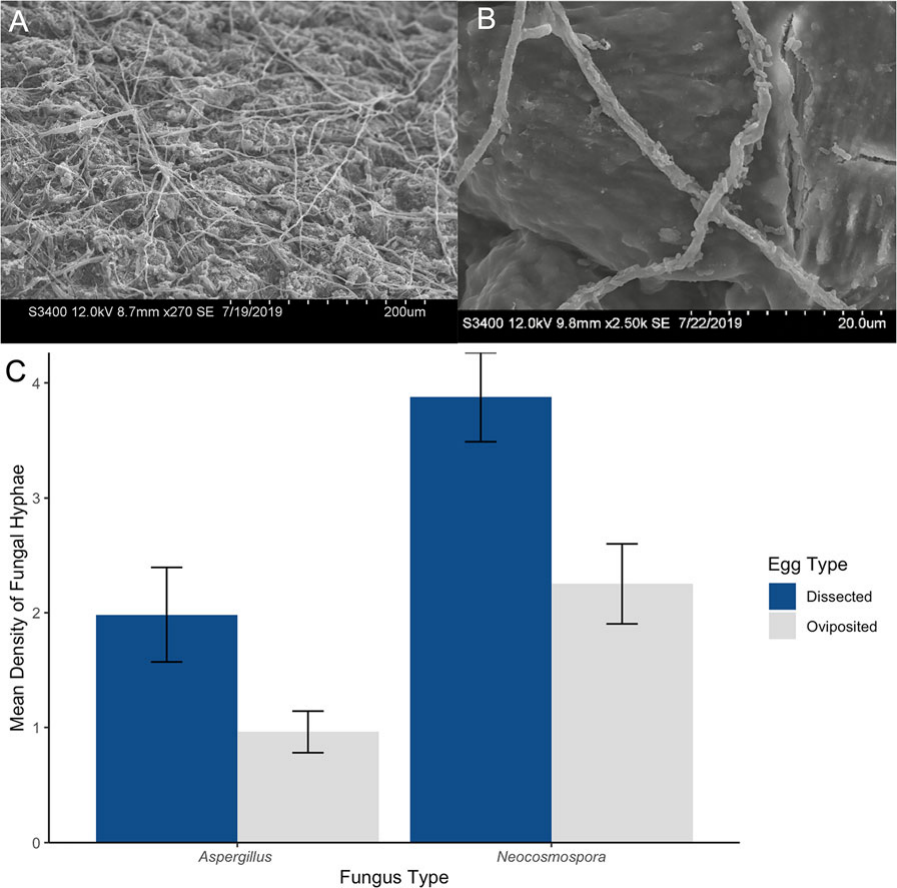
**A** Hyphal attachment of *Aspergillus protuberus* on an eggshell of *S. virgatus* at 270X magnification. **B** At 2.5 k magnification, we find hyphae covered in rod-shaped bacteria that are roughly 2 μm in length. **C** Mean (± SE) density of fungal hyphae (per 22,800 μm^2^) on dissected and oviposited *S. virgatus* eggs exposed to the fungal species *A. protuberus* and *Neocosmospora rubicola*. Fungal hyphae were significantly more dense on dissected eggs than oviposited eggs, for both *A. protuberus* (*p* = 0.012) and *N. rubicola* (*p* = 0.015)

Following a 48 h incubation with *Aspergillus protuberus* or *Neocosmospora rubicola*, fungal hyphae were identified on both dissected and oviposited eggshells (Fig. 7a) and in some cases were tightly associated with bacteria (Fig. 7b). For both fungal species, we found significantly more hyphae on dissected eggs than on oviposited eggs (*A. protuberus*: W = 62.5, *p* = 0.012; *N. rubicola*: t = 2.75, df = 15, *p* = 0.015; Fig. 7c).

### Hatch success and offspring phenotype

**Fig. 8:**
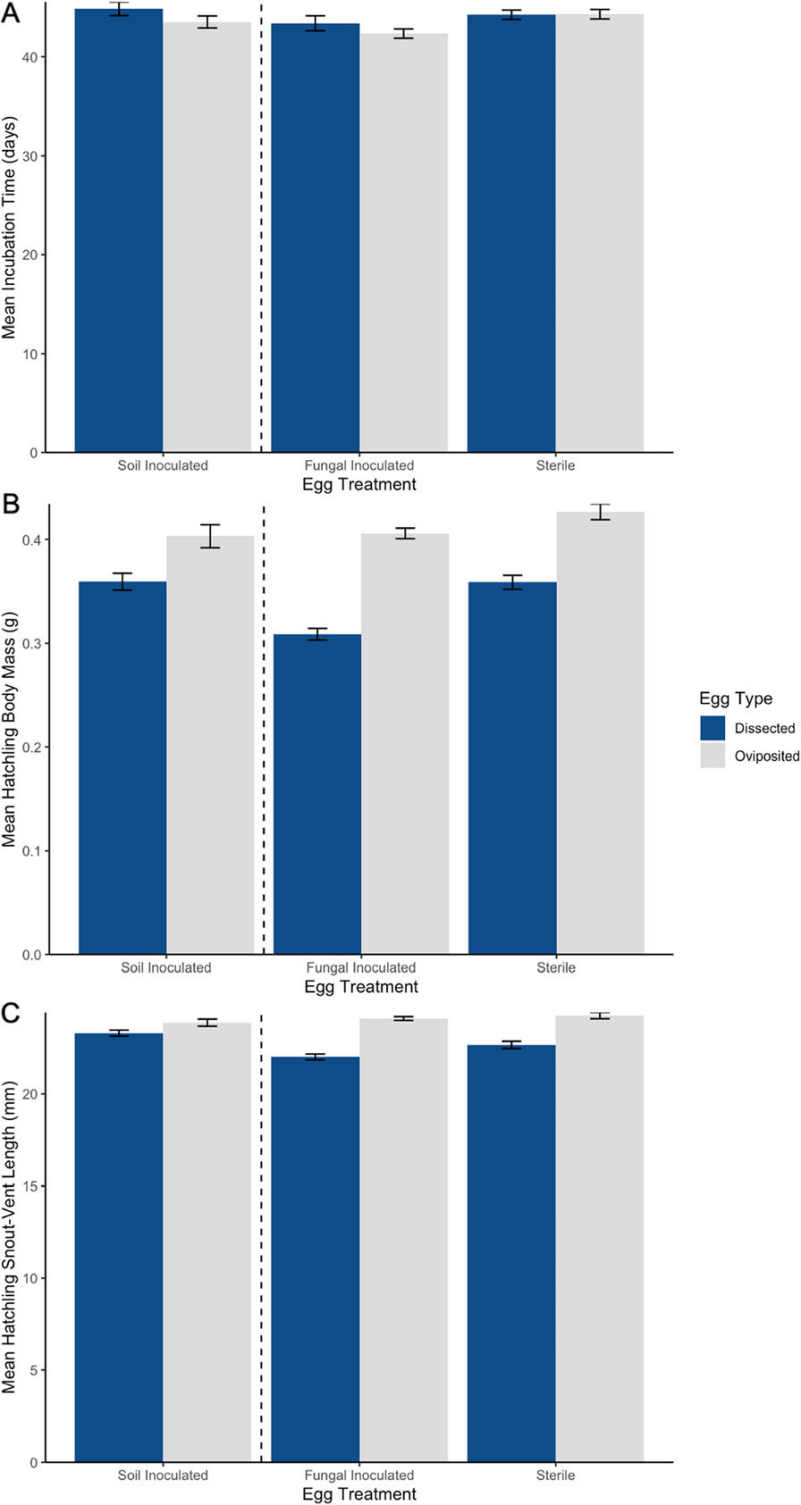
Mean (± SE) **A** incubation time, **B** body mass, and **C** SVL of hatchlings that emerged from dissected and oviposited *S. virgatus* eggs. In 2017, eggs were incubated in vermiculite inoculated with a soil slurry from the native range of *S. virgatus*. In 2019, eggs were incubated in vermiculite inoculated either with a fungal suspension derived from fungus that had infected failed *S. virgatus* eggs, or with sterile water. The dashed lined separates results from 2017 and 2019. Hatchlings from dissected and oviposited eggs did not differ in incubation size, but did significantly differ in mass and length

For the 2017 eggs, which were all exposed to naturally occurring microbes via a soil-inoculated vermiculite, hatch success was significantly lower for dissected eggs (42.3%) than oviposited eggs (86.9%; family: binomial, link: logit, z = − 4.19, *p* < < 0.001). Hatchlings that emerged from dissected eggs had similar hatch times (t = 1.21, *p* = 0.249) but were 24% smaller in mass (t = − 9.02, *p* < < 0.001) and 8% smaller in length (t = − 7.15, *p* < < 0.001) than hatchlings from oviposited eggs (Fig. 8).

For the 2019 eggs, at Day 25 of incubation, oviposited eggs had 100% viability, whereas dissected eggs had 73% viability. Viability of dissected eggs depended on incubation environment, with significantly higher viability in sterile (85%) than fungal-inoculated (61.0%) media (family: binomial, link: logit, z = − 2.36, *p* = 0.018). Hatch success was significantly lower in dissected eggs (59.8%) than in oviposited eggs (89.5%; family: binomial, link: logit, z = − 3.50, *p* < 0.001), whereas incubation environment did not significantly impact hatch success and was removed from the final model based on AIC.

Of hatched offspring, the effect of egg type on hatch time depended on the incubation environment (t = 2.45, *p* = 0.017, Fig. 8a). However, analyses investigating the effect of egg type in only sterile conditions and, separately, in only fungal-inoculated conditions both showed no significant pattern (sterile: t = 0.02, *p* = 0.982; fungal: t = − 0.95, *p* = 0.359, Fig. 8a).

As in 2017, hatchlings emerging from dissected eggs in 2019 were significantly smaller than those emerging from oviposited eggs, both in terms of body mass and length (Fig. 8b-c). Hatchlings from dissected eggs were 12% lighter than those from oviposited eggs (t = 2.42 *p* = 0.025; Fig. 8b), but hatchling body mass was unaffected by incubation environment (t = 0.26, *p* = 0.795) and the interaction of egg type and incubation environment (t = 1.40, *p* = 0.165). The effect of egg type on hatchling SVL depended on the incubation environment (t = 3.16, *p* = 0.002, Fig. 8c). Hatchlings from dissected eggs were 6% smaller in length than those from oviposited eggs in sterile conditions (t = 5.21, *p* = 0.001) but hatchlings from the two egg types did not differ in SVL when incubated in fungal-inoculated conditions (t = 1.29, *p* = 0.218). Note that mothers who produced dissected and oviposited eggs did not differ in size (mass: t = − 0.76, df = 20, *p* = 0.456, SVL: t = − 0.89, df = 20, *p* = 0.382).

## Discussion

Compared to oviposited eggs, dissected *S. virgatus* eggs that did not pass through the maternal cloaca had a lower bacterial density, lower bacterial diversity, a distinct microbial community, higher fungal density mid-incubation, and higher fungal attachment. These microbial differences appear to have an effect on female fitness by influencing egg viability, hatch success, and hatchling phenotype.

Amplicon sequencing revealed that microbiome on the eggshell of oviposited eggs is composed mostly of *Enterobacteriaceae* at both Day 0 and Day 25. Many members of this group have antifungal capabilities; in particular *Serratia* strains can prevent fungal growth and infection in various plant and animal systems [37–39]. In comparison, while dissected eggs began with a large proportion of *Enterobacteriaceae* on Day 0 (when no fungus was found), by Day 25 they had been largely colonized by other bacterial strains from the incubation environment, and they had higher fungal density. One of the key functions of the microbiome in other systems is to prevent pathogenic incursion through competition or direct intervention [40–43], and it is likely that one of those mechanisms is at work here.

The cloacal microbial community of *S. virgatus* lizards is also largely dominated by *Enterobacteriaceae* [21] and, among oviposited eggs, the diversity of eggshell microbes tended to correlate to the diversity of the maternal cloaca. These patterns, along with significantly higher bacterial loads on oviposited eggs relative to dissected eggs, support the hypothesis that microbes are transmitted directly from cloaca to egg during oviposition. Maternal transmission of microbes is a well-studied and important part of animal development, but such transmission for egg-laying animals has been less clear [8, 44]. Birds transfer microbes to their eggs, but often through nest tending behavior, rather than during oviposition [14, 19]. Sarmiento-Ramirez (2014) found sea turtle eggshells populated by some bacteria that also have been isolated from cloacae, although no direct comparison was made [17]. There is also evidence that in-ovo microbes can be passed down during egg development, and generally correlate to the maternal gut microbiome [13].

The microbiome has been shown to be highly localized in general, and in squamates specifically [10, 45, 46]. It is possible that in this system the cloaca acts as a bottleneck, to ensure that only specific beneficial bacteria are deposited upon eggs. The cloacae of wild *S. virgatus* have relatively low microbial diversity compared to other vertebrates [21]. This is unusual, as the cloaca is the terminus of the gastrointestinal and reproductive tracts, and thus has more often been shown to have higher diversity [45, 46]. This pattern could indicate positive selection for this particular cohort of bacteria, which offer benefits to the eggs during development.

One of those benefits appears to be preventing fungal attachment to eggs, which is a common cause of egg failure in many oviparous animals [47, 48]. We found that, along with higher bacterial loads, oviposited eggs were less likely to host fungus, even when incubated in an environment inoculated with fungus. Bacteria have been shown to prevent fungal growth in other systems, particularly soil bacteria that protect plant roots from fungal pathogens, and bacteria found on amphibian skin [37, 42, 49]. Generally, bacteria can disrupt fungal growth by breaking down mycelia through production of hydrolytic enzymes such as chitinase or protease [38].

As a further consequence of the bacterial and fungal attachment differences between dissected and oviposited eggs, dissected eggs had lower mid-incubation viability -- 15% lower than oviposited eggs when incubated in sterile conditions and 39% lower than oviposited eggs when incubated in fungal-inoculated conditions. Hatch success was also significantly lower in dissected eggs than in oviposited eggs, and the magnitude of the effect tended to increase with increased exposure to environmental pathogens. The effect of dissection on hatch success was a 24% reduction in sterile conditions, 45% reduction in fungal-inoculated conditions, and 52% reduction in soil-inoculated conditions. These patterns suggest that environmental pathogens are killing the eggs, and that maternal microbes offer protection.

Hatchlings that emerged from dissected eggs were significantly smaller than hatchlings from oviposited eggs. Smaller body size was not due to earlier hatching, which may occur in response to egg infection [50–52]. Rather, reduced size may be due to metabolic shifts in response to embryonic stress [53, 54]. As hatchling body size is likely to correlate to survival [55, 56], smaller hatchlings may be costly to female reproductive success. Thus, the vertically transmitted maternal microbiome may benefit female fitness both by increasing hatch success and by increasing the survival of those hatchlings.

Other mechanisms may also be at play. For instance, beneficial host-generated compounds secreted from cloacal glands [57] could be added to the eggshell during the final passage from oviduct to external environment, or perhaps a mechanical stimulus triggers some key embryonic developmental processes. These or other hypotheses could explain why dissection tended to reduce hatch success even in sterile environments. Though this pattern was not statistically significant, it suggests additional benefits occur during oviposition, beyond protection from fungal pathogens. None of these possibilities preclude antifungal protections, and indeed multiple influences on hatch success are likely to exist in any one system. What is evident here is that there is a clear relationship between oviposition, bacterial load, and offspring success.

## Conclusion

We have found compelling evidence for the vertical transmission of microbes with antifungal capabilities from the cloaca of *S. virgatus* females to their eggshells. Observational data using SEM show increased bacterial load and decreased fungal attachment on eggs that were laid via oviposition compared to those that were dissected. Experimental manipulation showed that this coincided with increased hatch success and offspring quality, while amplicon sequencing data confirmed that the composition of the eggshell microbiome is consistent with the core cloacal microbiome, and contains microbes known to have antifungal properties. We are currently working to quantify the antifungal effects by directly challenging bacteria found in the maternal cloaca with pathogenic environmental fungi in vitro. Next steps also include further examining the mechanism behind maternal transmission, as well as studies to document consequences of fungal infection in natural nests. This current study can be expanded to other species that face similar challenges to egg success, especially those that lack parental care, and could offer a new avenue for conservation and restoration research for other oviparous animals.

## Supplementary Information

**Additional file 1. **Effect of antibiotic treatment on the cloacal microbiome of female *Sceloporus virgatus*.

**Additional file 2.** R script used to process sequences from 2017 samples discussed in Additional file 1

**Additional file 3.** R script used to process all sequences from 2019 samples discussed in the main text.

**Additional file 4.** R script for statistical analyses of all data derived from sequencing: richness, Shannon diversity, beta diversity, and differential abundance analyses.

**Additional file 5.** R script for statistical analyses of all experimental data: SEM analyses, fungal attachment assays, hatch success, and hatchling phenotype.

**Additional file 6.** Taxonomy assignments for all ASV’s.

**Additional file 7.** Community composition data for cloacal and eggshell samples.

## Data Availability

The sequences supporting the conclusions of this study, including biological samples, sampling controls, extraction controls, PCR controls, and mock communities, are available in the sequence read archive of NCBI (BioProject PRJNA687039, http://www.ncbi.nlm.nih.gov/bioproject/687039, SAMN17131990–17132125). R scripts for processing sequences and for all statistical analyses used in this study are included as Additional files 2, 3, 4, and 5. The taxonomy assignments for all ASVs, following processing and removal of contaminants, are in Additional file 6. Community composition data for cloacal and eggshell samples are included in Additional file 7.

## Abbreviations

ASV: Amplicon sequence variant
NMDS: Non-metric multidimensional scaling
PBS: Phosphate buffer solution
PCR: Polymerase chain reaction
SEM: Scanning electron microscope
SVL: Snout to vent length
SWRS: Southwestern Research Station
