# Supplementary material for: Vertically transmitted microbiome protects eggs from fungal infection and egg failure
Source: Anim Microbiome. 2021 Jun 16;3:43. doi: 10.1186/s42523-021-00104-5 (PMC8207602; doi:10.1186/s42523-021-00104-5)
Supplement: Supplementary file 1 — Additional file 1. Effect of antibiotic treatment on the cloacal microbiome of female Sceloporus virgatus. [file 42523_2021_104_MOESM1_ESM.pdf]

Additional file 1: Effect of antibiotic treatment on the cloacal microbiome of female *Sceloporus virgatus*

Methods

We collected gravid *Sceloporus virgatus* females using a loop of fishing line tied to a variable length fishing pole from areas surrounding the American Museum of Natural History's Southwestern Research Station (SWRS) in Cochise County, Arizona between June 27 and July 4, 2017. Lizards were kept in large outdoor enclosures at SWRS. We randomly assigned animals to treatment group for a study originally designed to compare the effect of reduced eggshell bacterial load on hatch success and hatchling phenotype. Here, we only present data on the effectiveness of our antibiotic treatment on the cloacal microbiome. Antibiotic-treated females ($n = 8$) received 0.03 ml of compounded 2 mg/mL enrofloxacin orally for 3 d, July 5-7. Two other groups of females (Control and Dissected; $n = 7$ for each) received no treatment and differed only by the means of egg acquisition, not relevant to data presented here.

On July 8, each animal had their cloaca swabbed by gently inserting a sterile swab (BD ESwab™) into the cloaca and slowly rotating it. Microbes were eluted into an Amies solution, half of which was stored at -80°C for high-throughput sequencing. (The remaining half was used for other work.) Samples were extracted, amplified, and sequenced, and the sequence results were processed in tandem with other samples in this manuscript, following the same methods. Differences in alpha diversity between groups was compared with a one-way ANOVA.

Dispersion of communities was tested with betadisper and beta diversity was compared with a PERMANOVA, both from the vegan package [1].

Results

The cloacal microbiome of Antibiotic-treated, Dissected, and Control females did not differ in richness (F = 2.04, df = 2,19, p = 0.158, Fig. S1a), nor alpha diversity (F = 1.54, df = 2,19, p = 0.241, Fig. 1b), though trends suggested antibiotic treatment may have increased colonization of the cloaca from environmental bacteria. The cloacal microbial communities of the three groups of females did not differ in community dispersion (F = 1.51, df = 2,19, p = 0.246), but did differ slightly in beta diversity (F = 2.28, df = 2,19, p = 0.036; Fig. S1c). The same patterns were found when the two untreated groups (Dissected and Control) were combined.

The cloacae of untreated animals were largely populated by *Enterobacteriaceae*, making up 82.0 ± 6.0% of the community on average. The next most abundant taxa was *Helicobacteraceae*, which made up 9.5 ± 4.2% on average. No other families were greater than 1% of the community on average. *Enterobacteriaceae* was also the most abundant taxa in the antibiotic treated animals, but comprised a smaller percent of the community at 44.7 ± 16.7%. The next most abundant taxon was *Lachnospiraceae* (18.5% ± 7.8%), closely followed by *Bacteroidaceae* (13.1% ± 5.6%). There were also several families that made up between 1-10% of the community, including *Helicobacteraceae, Enterococcaceae* and *Tannerellaceae.*

References

1. Oksanen J, Blanchet FG, Friendly M, Kindt R, Legendre P, McGlinn D, et al. vegan: Community Ecology Package. 2019. https://CRAN.R-project.org/package=vegan. Accessed 17 Sep 2020.

Figure

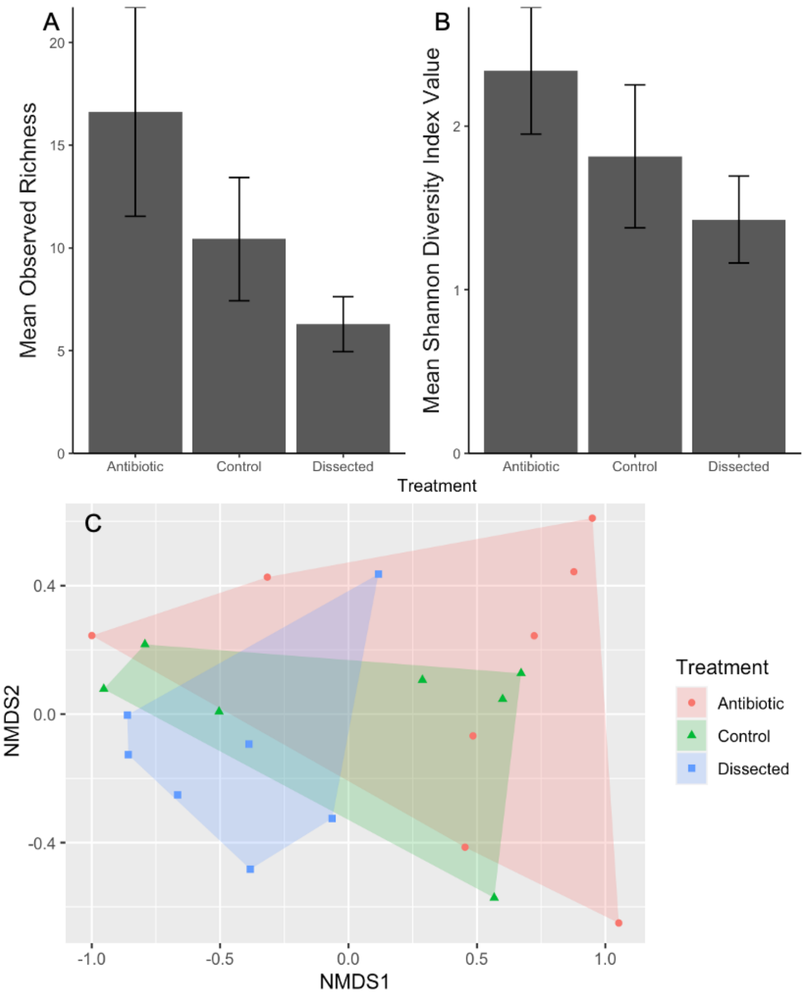

Figure 1. A) Mean observed richness (±SE) and B) Mean Shannon diversity index values (±SE)

from cloacal swabs of three groups of *S. virgatus* females. Females in the Antibiotic group had received 3 d of treatment with 0.03 ml of compounded 2 mg/mL enrofloxacin prior to sampling whereas the other two groups were unmanipulated. C) Non-metric multidimensional scaling plots were created by using Bray Curtis distance to calculate pairwise distances based on community composition.
