# Supplementary material for: Vertically transmitted microbiome protects eggs from fungal infection and egg failure
Source: Anim Microbiome. 2021 Jun 16;3:43. doi: 10.1186/s42523-021-00104-5 (PMC8207602; doi:10.1186/s42523-021-00104-5)
Supplement: Supplementary file 2 — Additional file 2. R script used to process sequences from 2017 samples discussed in Additional file 1 [file 42523_2021_104_MOESM2_ESM.pdf]

2017 HTS Data Prep

M.E. Bunker and S. L. Weiss

12/15/2020

Packages Used

```
library(dada2)
```

```
## Loading required package: Rcpp
```

```
library(tidyverse)
```

```
## ── Attaching packages ─────────────────────────────────────── tidyverse 1.3.0 ──
```

```
## ✓ ggplot2 3.3.3     ✓ purrr   0.3.4
## ✓ tibble  3.1.0     ✓ dplyr   1.0.5
## ✓ tidyr   1.1.3     ✓ stringr 1.4.0
## ✓ readr   1.4.0     ✓ forcats 0.5.1
```

```
## ── Conflicts ────────────────────────────────────────── tidyverse_conflicts() ──
## x dplyr::filter() masks stats::filter()
## x dplyr::lag()    masks stats::lag()
```

```
library(dplyr)
library(phyloseq)
library(corncob)
library(lme4)
```

```
## Loading required package: Matrix
```

```
##
## Attaching package: 'Matrix'
```

```
## The following objects are masked from 'package:tidyr':
##
##     expand, pack, unpack
```

```
library(decontam)
library(ggplot2)
library(vegan)
```

```
## Loading required package: permute
```

```
## Loading required package: lattice
```

```
## This is vegan 2.5-7
```

Raw Data Prep

Samples

```
path <- "2017_Raw/"

head(list.files(path))
```

```
## [1] "103_R1.fastq.gz" "103_R2.fastq.gz" "107_R1.fastq.gz" "107_R2.fastq.gz"
## [5] "69_R1.fastq.gz"  "69_R2.fastq.gz"
```

```
for.names <- sort(list.files(path, pattern = "_R1.fastq", full.names = TRUE))
rev.names <- sort(list.files(path, pattern = "_R2.fastq", full.names = TRUE))
sample.names <- sapply(strsplit(basename(for.names), "_R"), '[', 1)
sample.names
```

```
##  [1] "103"           "107"           "69"            "85"
##  [5] "89"            "95"            "EE101"         "EE105"
##  [9] "EE109"         "EE111"         "EE113"         "EE71"
## [13] "EE73"          "EE75"          "EE77"          "EE79"
## [17] "EE81"          "EE83"          "EE87"          "EE91"
## [21] "EE97"          "EE99"          "PCR1Negative1" "PCR1Negative2"
## [25] "PCR1Negative3" "PCR1Negative4" "PCR1Negative5" "PCR1Negative6"
## [29] "PCR2Negative1" "PCR2Negative3" "PCR2Negative5" "PCR2Negative7"
## [33] "X1"            "X11AS"         "X16"           "X4"
## [37] "X7A"
```

```
length(sample.names)
```

```
## [1] 37
```

```
for.filt <- file.path(path, "filtered", paste0(sample.names, "_F_filt.fastq"))
rev.filt <- file.path(path, "filtered", paste0(sample.names, "_R_filt.fastq"))
filt.out <- filterAndTrim(for.names, for.filt, rev.names, rev.filt,
                          truncLen = c(265,185), maxEE = c(2,2),
                          compress = TRUE,
                          multithread = TRUE)
```

```
## Creating output directory: 2017_Raw//filtered
```

```
err.f <- learnErrors(for.filt, multithread = TRUE)
```

```
## 56147140 total bases in 211876 reads from 37 samples will be used for learning the error rate
s.
```

```
err.r <- learnErrors(rev.filt, multithread = TRUE)
```

```
## 39197060 total bases in 211876 reads from 37 samples will be used for learning the error rate
s.
```

```
plotErrors(err.f, nominalQ = TRUE)
```

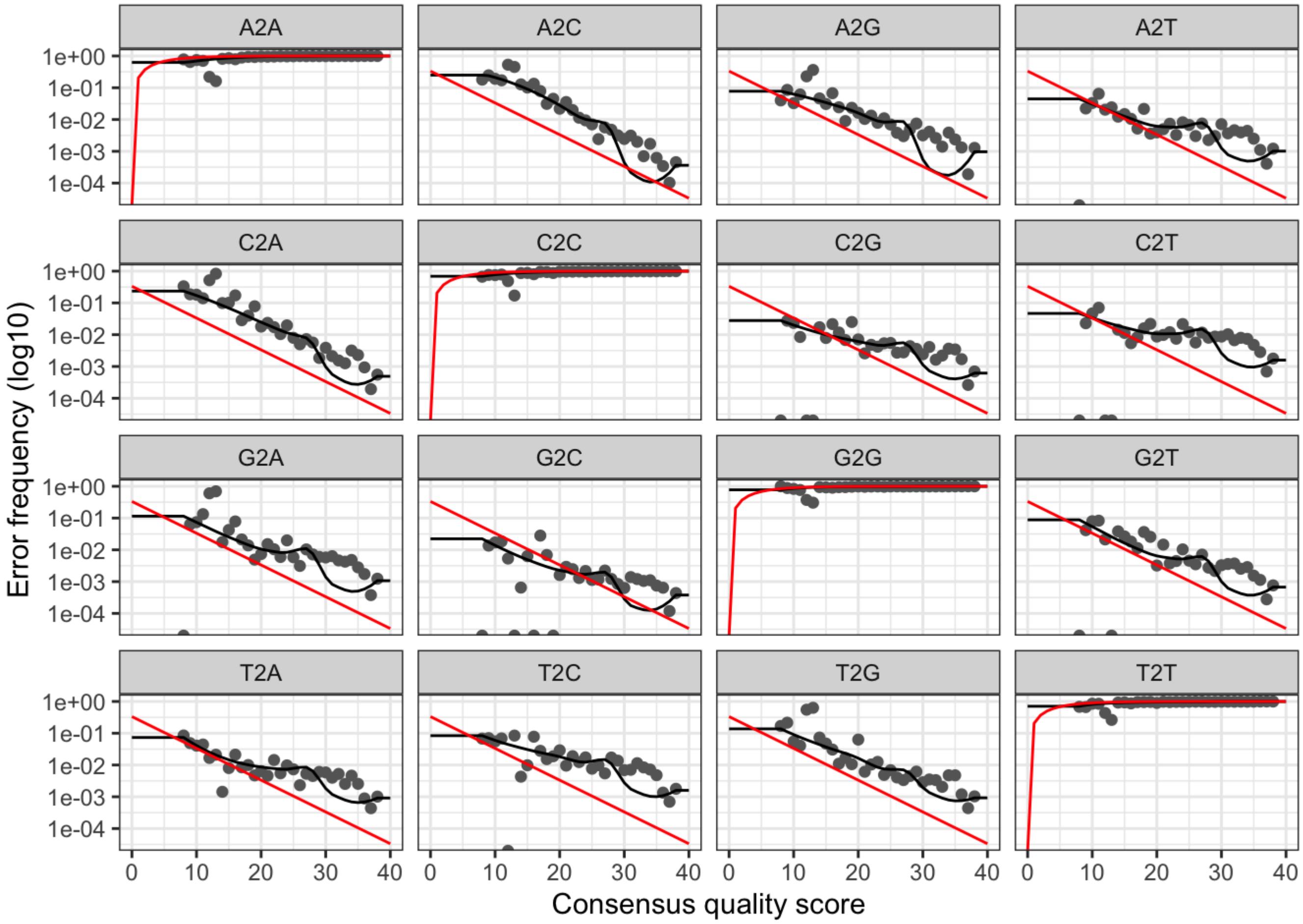


```
plotErrors(err.r, nominalQ = TRUE)
```

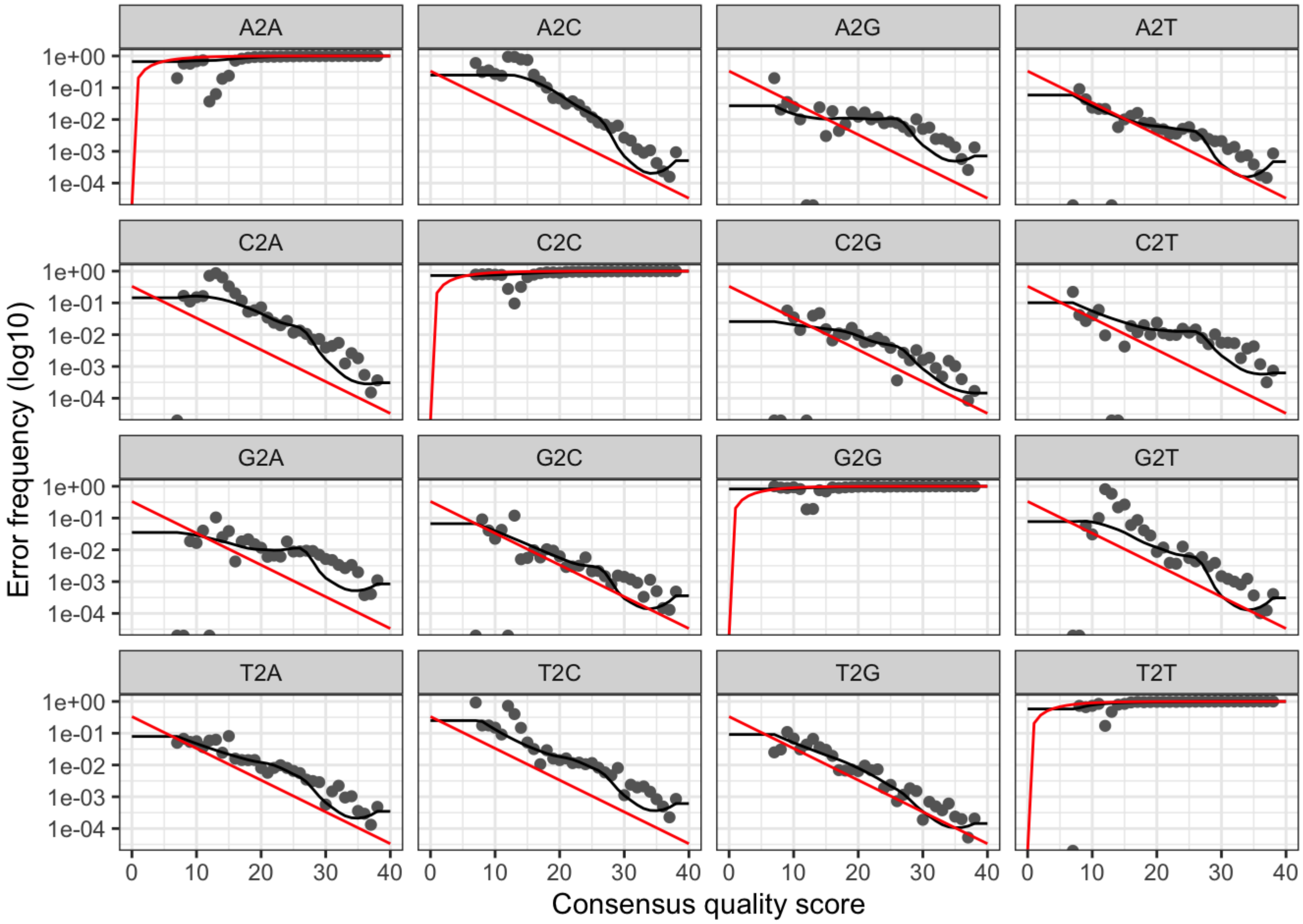


```
for.derep <- derepFastq(for.filt)
rev.derep <- derepFastq(rev.filt)
names(for.derep) <- sample.names
names(rev.derep) <- sample.names
```

```
for.dada <- dada(for.derep, err = err.f, multithread = TRUE,verbose = TRUE)
```

```
## Sample 1 - 3666 reads in 1590 unique sequences.
## Sample 2 - 2567 reads in 658 unique sequences.
## Sample 3 - 1164 reads in 341 unique sequences.
## Sample 4 - 2399 reads in 805 unique sequences.
## Sample 5 - 4093 reads in 889 unique sequences.
## Sample 6 - 3800 reads in 1061 unique sequences.
## Sample 7 - 9377 reads in 1232 unique sequences.
## Sample 8 - 10825 reads in 1393 unique sequences.
## Sample 9 - 2085 reads in 757 unique sequences.
## Sample 10 - 3820 reads in 646 unique sequences.
## Sample 11 - 47729 reads in 5089 unique sequences.
## Sample 12 - 11573 reads in 1690 unique sequences.
## Sample 13 - 6560 reads in 1641 unique sequences.
## Sample 14 - 20073 reads in 2431 unique sequences.
## Sample 15 - 9901 reads in 2410 unique sequences.
## Sample 16 - 8829 reads in 1162 unique sequences.
## Sample 17 - 5379 reads in 1391 unique sequences.
## Sample 18 - 1247 reads in 336 unique sequences.
## Sample 19 - 13515 reads in 1724 unique sequences.
## Sample 20 - 6462 reads in 992 unique sequences.
## Sample 21 - 8291 reads in 1588 unique sequences.
## Sample 22 - 26523 reads in 4214 unique sequences.
## Sample 23 - 376 reads in 96 unique sequences.
## Sample 24 - 63 reads in 32 unique sequences.
## Sample 25 - 193 reads in 60 unique sequences.
## Sample 26 - 129 reads in 43 unique sequences.
## Sample 27 - 90 reads in 39 unique sequences.
## Sample 28 - 19 reads in 17 unique sequences.
## Sample 29 - 3 reads in 3 unique sequences.
## Sample 30 - 6 reads in 6 unique sequences.
## Sample 31 - 10 reads in 10 unique sequences.
## Sample 32 - 7 reads in 7 unique sequences.
## Sample 33 - 347 reads in 89 unique sequences.
## Sample 34 - 194 reads in 75 unique sequences.
## Sample 35 - 38 reads in 36 unique sequences.
## Sample 36 - 278 reads in 77 unique sequences.
## Sample 37 - 245 reads in 121 unique sequences.
```

```
rev.dada <- dada(rev.derep, err = err.r, multithread = TRUE, verbose = TRUE)
```

```
## Sample 1 - 3666 reads in 1063 unique sequences.
## Sample 2 - 2567 reads in 465 unique sequences.
## Sample 3 - 1164 reads in 271 unique sequences.
## Sample 4 - 2399 reads in 629 unique sequences.
## Sample 5 - 4093 reads in 580 unique sequences.
## Sample 6 - 3800 reads in 696 unique sequences.
## Sample 7 - 9377 reads in 756 unique sequences.
## Sample 8 - 10825 reads in 874 unique sequences.
## Sample 9 - 2085 reads in 428 unique sequences.
## Sample 10 - 3820 reads in 460 unique sequences.
## Sample 11 - 47729 reads in 3329 unique sequences.
## Sample 12 - 11573 reads in 1215 unique sequences.
## Sample 13 - 6560 reads in 1052 unique sequences.
## Sample 14 - 20073 reads in 1533 unique sequences.
## Sample 15 - 9901 reads in 1532 unique sequences.
## Sample 16 - 8829 reads in 794 unique sequences.
## Sample 17 - 5379 reads in 904 unique sequences.
## Sample 18 - 1247 reads in 222 unique sequences.
## Sample 19 - 13515 reads in 1037 unique sequences.
## Sample 20 - 6462 reads in 680 unique sequences.
## Sample 21 - 8291 reads in 1072 unique sequences.
## Sample 22 - 26523 reads in 2677 unique sequences.
## Sample 23 - 376 reads in 83 unique sequences.
## Sample 24 - 63 reads in 24 unique sequences.
## Sample 25 - 193 reads in 54 unique sequences.
## Sample 26 - 129 reads in 35 unique sequences.
## Sample 27 - 90 reads in 29 unique sequences.
## Sample 28 - 19 reads in 12 unique sequences.
## Sample 29 - 3 reads in 3 unique sequences.
## Sample 30 - 6 reads in 6 unique sequences.
## Sample 31 - 10 reads in 10 unique sequences.
## Sample 32 - 7 reads in 5 unique sequences.
## Sample 33 - 347 reads in 84 unique sequences.
## Sample 34 - 194 reads in 73 unique sequences.
## Sample 35 - 38 reads in 30 unique sequences.
## Sample 36 - 278 reads in 74 unique sequences.
## Sample 37 - 245 reads in 94 unique sequences.
```

```
ee.merged <- mergePairs(for.dada, for.derep, rev.dada, rev.derep, verbose = FALSE)
ee.seqtab <- makeSequenceTable(ee.merged)

ee.seq.nochim <- removeBimeraDenovo(ee.seqtab, method = "per-sample",
                                     multithread = TRUE)
```

```
ee.taxa <- assignTaxonomy(ee.seq.nochim, "~/Desktop/silva_nr_v132_train_set.fa.gz")
head(unname(ee.taxa))
```

```
##      [,1]       [,2]             [,3]                  [,4]
## [1,] "Bacteria" "Proteobacteria" "Gammaproteobacteria" "Enterobacteriales"
## [2,] "Bacteria" "Proteobacteria" "Gammaproteobacteria" "Enterobacteriales"
## [3,] "Bacteria" "Proteobacteria" "Gammaproteobacteria" "Enterobacteriales"
## [4,] "Bacteria" "Proteobacteria" "Gammaproteobacteria" "Enterobacteriales"
## [5,] "Bacteria" "Proteobacteria" "Gammaproteobacteria" "Enterobacteriales"
## [6,] "Bacteria" "Proteobacteria" "Gammaproteobacteria" "Enterobacteriales"
##      [,5]                 [,6]
## [1,] "Enterobacteriaceae" NA
## [2,] "Enterobacteriaceae" NA
## [3,] "Enterobacteriaceae" NA
## [4,] "Enterobacteriaceae" "Izhakiella"
## [5,] "Enterobacteriaceae" "Izhakiella"
## [6,] "Enterobacteriaceae" "Izhakiella"
```

```
write.csv(ee.taxa, "R_files/taxa_with_seqs.csv")
ee.asv <- colnames(ee.seq.nochim)
asv.headers <- vector(dim(ee.seq.nochim)[2], mode = "character")
for(i in 1:dim(ee.seq.nochim)[2]){
  asv.headers[i] <- paste(">ASV", i, sep =  "_")
}
head(asv.headers)
```

```
## [1] ">ASV_1" ">ASV_2" ">ASV_3" ">ASV_4" ">ASV_5" ">ASV_6"
```

```
ee.asv.fasta <- c(rbind(asv.headers, ee.asv))

asv.tab <- t(ee.seq.nochim)
row.names(asv.tab) <- sub(">", "", asv.headers)

asv.tax <- ee.taxa
row.names(asv.tax) <- sub(">", "", asv.headers)
```

Decontaminate

```
ee.meta <- read.csv("R_files/ee_meta_all.csv", row.names = 1)
ee.meta <- ee.meta[order(rownames(ee.meta)),]
control.vector <- ee.meta$is.control
contam.df <- isContaminant(t(asv.tab), neg = control.vector, threshold = 0.1)
contam.asv <- row.names(contam.df[contam.df$contaminant == TRUE,])
length(contam.asv)
```

```
## [1] 6
```

```
dim(asv.tab)
```

```
## [1] 763  37
```

```
contam.index <- which(ee.asv.fasta %in% paste0(">", contam.asv))
no <- sort(c(contam.index, contam.index +1))
ee.asv.decontam <- ee.asv.fasta[- no]
ee.counts.decontam <- asv.tab[!row.names(asv.tab) %in% contam.asv, ]
ee.tax.decontam <- asv.tax[!row.names(asv.tax) %in% contam.asv, ]
```

```
ee.meta <- ee.meta[ee.meta$is.control == "FALSE",]

write.csv(ee.meta, "R_files/ee_meta_samples.csv")
write(ee.asv.decontam, "R_files/ee_asv_decontam.fa")
write.csv(ee.counts.decontam, "R_files/ee_counts_decontam.csv")
write.csv(ee.tax.decontam, "R_files/ee_tax_decontam.csv")
```

Move to analysis document
