# Supplementary material for: Vertically transmitted microbiome protects eggs from fungal infection and egg failure
Source: Anim Microbiome. 2021 Jun 16;3:43. doi: 10.1186/s42523-021-00104-5 (PMC8207602; doi:10.1186/s42523-021-00104-5)
Supplement: Supplementary file 3 — Additional file 3. R script used to process all sequences from 2019 samples discussed in the main text. [file 42523_2021_104_MOESM3_ESM.pdf]

2019 HTS Data Prep

M.E. Bunker and S. L. Weiss

12/15/2020

Packages used

```
library(dada2)
library(tidyverse)
library(dplyr)
library(phyloseq)
library(corncob)
library(lme4)
library(decontam)
library(ggplot2)
library(vegan)
```

Raw Data Prep

Samples

```
path <- "2019_Raw/"
head(list.files(path))
```

```
## [1] "filtered"         "GH01_R1.fastq.gz" "GH01_R2.fastq.gz" "GH02_R1.fastq.gz"
## [5] "GH02_R2.fastq.gz" "GH03_R1.fastq.gz"
```

```
for.names <- sort(list.files(path, pattern = "_R1.fastq", full.names = TRUE))
rev.names <- sort(list.files(path, pattern = "_R2.fastq", full.names = TRUE))
sample.names <- sapply(strsplit(basename(for.names), "_R"), '[', 1)
sample.names
```

```
##   [1] "GH01"         "GH02"         "GH03"         "GH04"         "GH05"
##   [6] "GH06"         "GH07"         "GH08"         "GH09"         "GH10"
##  [11] "GH11"         "GH12"         "GH13"         "GH14"         "GH15"
##  [16] "GH16"         "GH17"         "GH18"         "GH19"         "GH19control"
##  [21] "GH19control1" "GH19control2" "GH19control3" "GH20"         "GH21"
##  [26] "GH22"         "GH23"         "GH24"         "GH25"         "GH26"
##  [31] "GH27"         "GH28"         "GH29"         "GH30"         "GH31"
##  [36] "GH32"         "GH33"         "GH34"         "GH35"         "GH36"
##  [41] "GH37"         "GH38"         "GH39"         "GH41"         "GH42"
##  [46] "GH43"         "GH45"         "GH46"         "GH47"         "GH48"
##  [51] "GH49"         "GH50"         "GH53"         "IES_02"       "IES_06"
##  [56] "IES_08"       "IES_12"       "IES_13"       "IES_18"       "IES_20"
##  [61] "IES_26"       "IES_28"       "IES_32"       "IES_36"       "IES_38"
##  [66] "IES_40"       "IES_41"       "IES_44"       "IES_46"       "IES_50"
##  [71] "IES_52"       "IES_54"       "IES_56"       "IES_58"       "IES_60"
##  [76] "IES_64"       "IES_66"       "IES_68"       "IES_70"       "IES_72"
##  [81] "IES_74"       "IES_76"       "IES_78"       "MBPBS4"       "MBPBS5"
##  [86] "MBXPool1"     "MBXPool2"     "MBXPool3"     "PCR1neg1"     "PCR1neg10"
##  [91] "PCR1neg11"    "PCR1neg12"    "PCR1neg13"    "PCR1neg14"    "PCR1neg2"
##  [96] "PCR1neg3"     "PCR1neg4"     "PCR1neg43"    "PCR1neg5"     "PCR1neg6"
## [101] "PCR1neg7"     "PCR1neg8"     "PCR1neg9"
```

```
length(sample.names)
```

```
## [1] 103
```

DADA2

```
for.filt <- file.path(path, "filtered", paste0(sample.names, "_F_filt.fastq"))
rev.filt <- file.path(path, "filtered", paste0(sample.names, "_R_filt.fastq"))
filt.out <- filterAndTrim(for.names, for.filt, rev.names, rev.filt,
                          truncLen = c(270,175), maxEE = c(2,2),
                          compress = TRUE,
                          multithread = TRUE)
```

There are 103 samples total, 79 swab and shell samples, and 4 experimental controls, and 20 lab controls.

```
err.f <- learnErrors(for.filt, multithread = TRUE)
```

```
## 117387630 total bases in 434769 reads from 20 samples will be used for learning the error rat
es.
```

```
err.r <- learnErrors(rev.filt, multithread = TRUE)
```

```
## 100100350 total bases in 572002 reads from 23 samples will be used for learning the error rat
es.
```

```
plotErrors(err.f, nominalQ = TRUE)
```

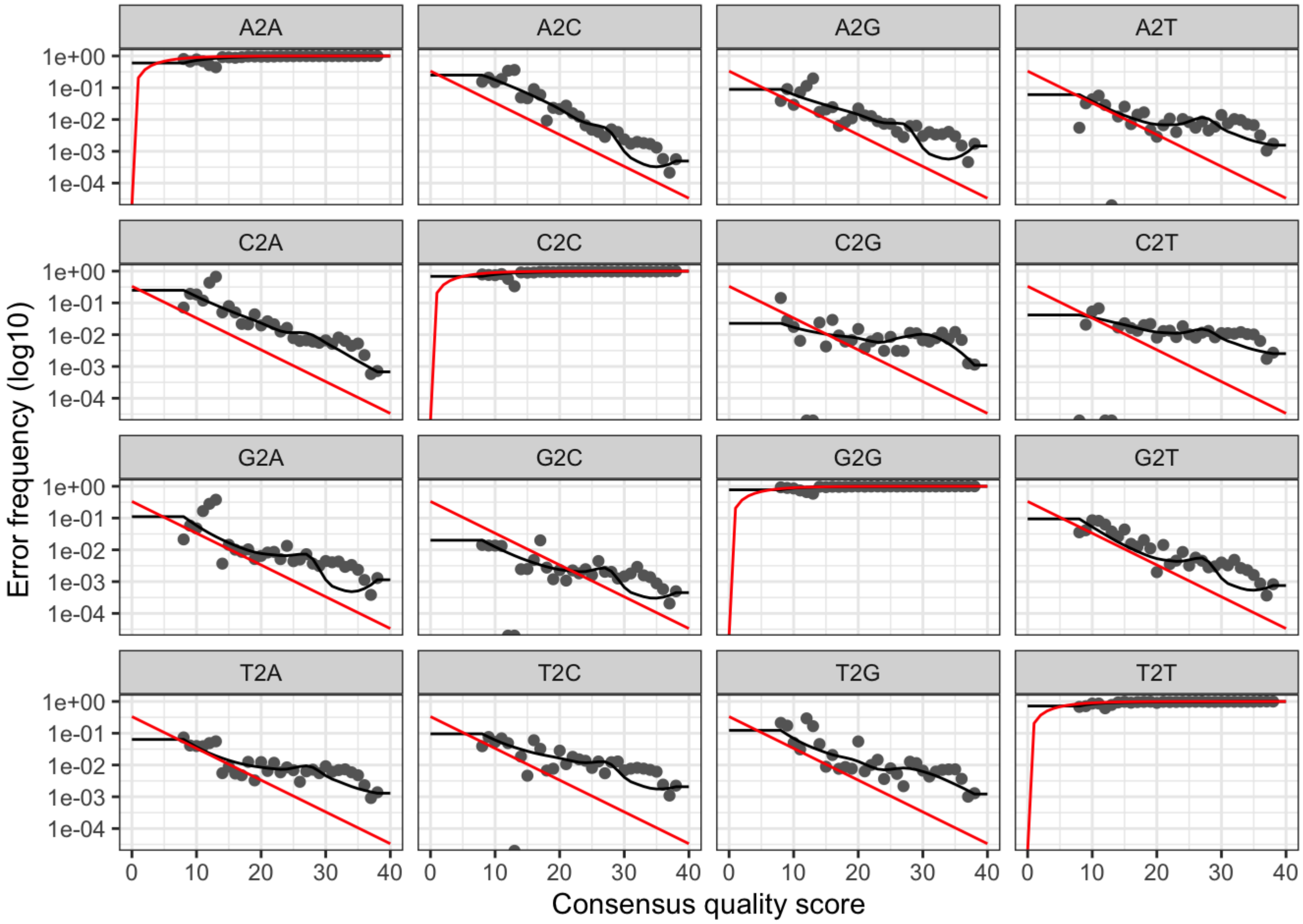


```
plotErrors(err.r, nominalQ = TRUE)
```

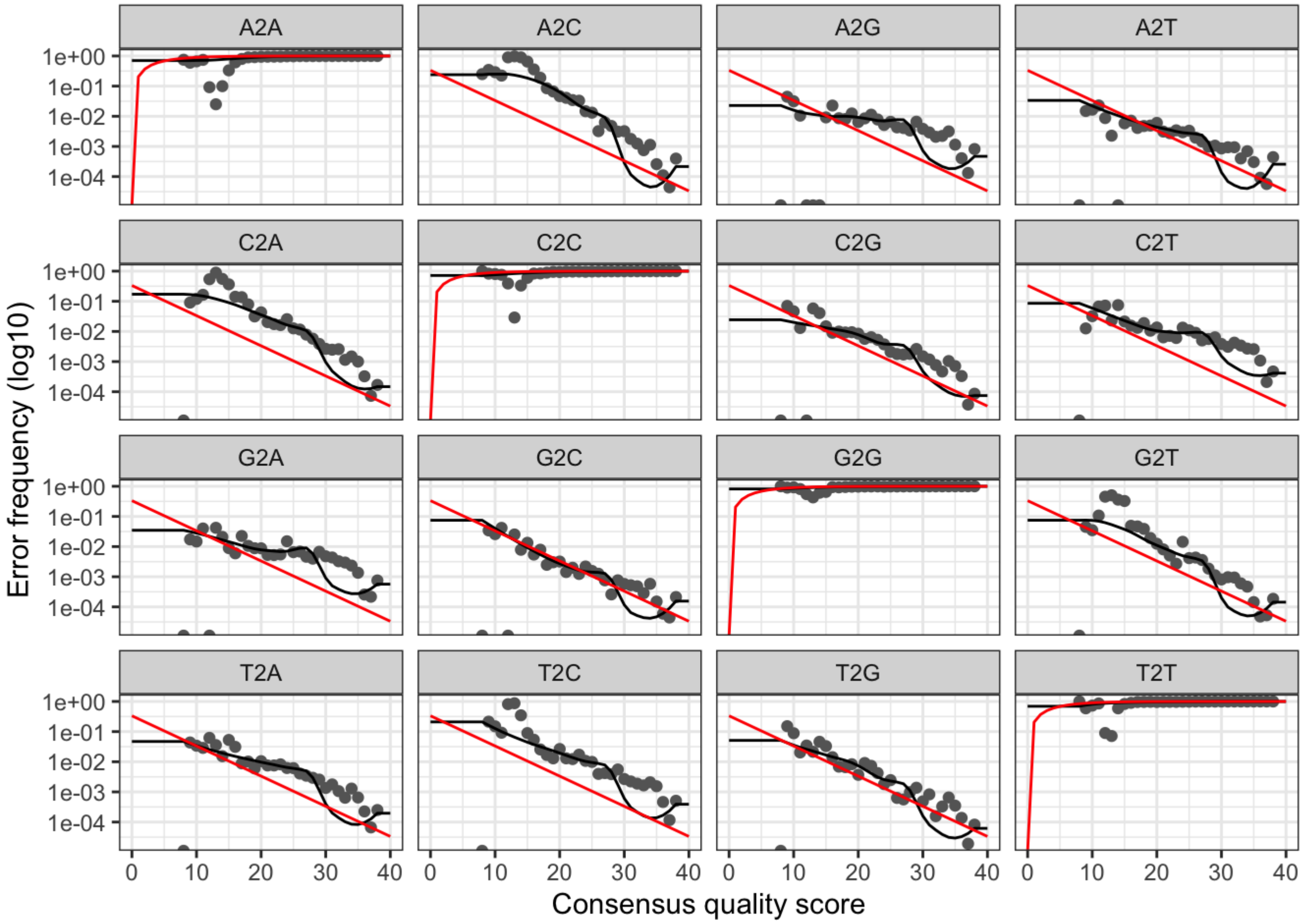


```
for.derep <- derepFastq(for.filt)
rev.derep <- derepFastq(rev.filt)
names(for.derep) <- sample.names
names(rev.derep) <- sample.names
```

```
for.dada <- dada(for.derep, err = err.f, multithread = TRUE, verbose = FALSE)
rev.dada <- dada(rev.derep, err = err.r, multithread = TRUE, verbose = FALSE)
```

```
gh.merged <- mergePairs(for.dada, for.derep, rev.dada, rev.derep, verbose = FALSE)
gh.seqtab <- makeSequenceTable(gh.merged)
```

```
gh.seq.nochim <- removeBimeraDenovo(gh.seqtab, method = "per-sample",
                                    multithread = TRUE,
                                    verbose = FALSE)
```

```
gh.taxa <- assignTaxonomy(gh.seq.nochim, "silva_nr_v132_train_set.fa.gz")
```

```
write.csv(gh.seq.nochim,"counts_with_seqs.csv")
gh.asv <- colnames(gh.seq.nochim)
asv.headers <- vector(dim(gh.seq.nochim)[2], mode = "character")
for(i in 1:dim(gh.seq.nochim)[2]){
  asv.headers[i] <- paste(">ASV", i, sep =  "_")
}
head(asv.headers)
```

```
## [1] ">ASV_1" ">ASV_2" ">ASV_3" ">ASV_4" ">ASV_5" ">ASV_6"
```

```
gh.asv.fasta <- c(rbind(asv.headers, gh.asv))

asv.tab <- t(gh.seq.nochim)
row.names(asv.tab) <- sub(">", "", asv.headers)

asv.tax <- gh.taxa
row.names(asv.tax) <- sub(">", "", asv.headers)
```

Decontaminate

```
gh.meta <- read.csv("R_files/gh19_meta.csv", row.names = 1)
gh.meta <- gh.meta[order(rownames(gh.meta)),]
```

```
control.vector <- gh.meta$is.control
```

```
contam.df <- isContaminant(t(asv.tab), neg = control.vector, threshold = 0.1)
```

```
## Warning in isContaminant(t(asv.tab), neg = control.vector, threshold = 0.1):
## Removed 8 samples with zero total counts (or frequency).
```

```
contam.asv <- row.names(contam.df[contam.df$contaminant == TRUE,])
length(contam.asv)
```

```
## [1] 158
```

```
dim(asv.tab)
```

```
## [1] 4853  103
```

```
contam.index <- which(gh.asv.fasta %in% paste0(">", contam.asv))
no <- sort(c(contam.index, contam.index +1))
gh.asv.decontam <- gh.asv.fasta[- no]
gh.counts.decontam <- asv.tab[!row.names(asv.tab) %in% contam.asv, ]
gh.tax.decontam <- asv.tax[!row.names(asv.tax) %in% contam.asv, ]
```

```
gh.meta <- gh.meta[gh.meta$is.control == "FALSE",]
gh.counts.decontam <- gh.counts.decontam[,colnames(gh.counts.decontam) %in% rownames(gh.meta)]

write.csv(gh.meta, "R_files/gh_meta_samples.csv")
write(gh.asv.decontam, "R_files/gh_asv_decontam.fa")
write.csv(gh.counts.decontam, "R_files/gh_counts_decontam.csv")
write.csv(gh.tax.decontam, "R_files/gh_tax_decontam.csv")
```

Move on to analysis document
