# Supplementary material for: Vertically transmitted microbiome protects eggs from fungal infection and egg failure
Source: Anim Microbiome. 2021 Jun 16;3:43. doi: 10.1186/s42523-021-00104-5 (PMC8207602; doi:10.1186/s42523-021-00104-5)
Supplement: Supplementary file 4 — Additional file 4. R script for statistical analyses of all data derived from sequencing: richness, Shannon diversity, beta diversity, and differential abundance analyses. [file 42523_2021_104_MOESM4_ESM.pdf]

Vertically transmitted microbiome protects eggs from fungal infection and egg failure: HTS Data Analysis

M.E. Bunker, G. Elliott, H. Heyer-Gray, M. O. Martin, A. E. Arnold, and S. L. Weiss

12/15/2020

Packagess

```
library(tidyverse)
```

```
## ── Attaching packages ─────────────────────── tidyverse 1.3.0 ──
```

```
## ✓ ggplot2 3.3.2     ✓ purrr   0.3.4
## ✓ tibble  3.0.3     ✓ dplyr   1.0.2
## ✓ tidyr   1.1.2     ✓ stringr 1.4.0
## ✓ readr   1.3.1     ✓ forcats 0.5.0
```

```
## ── Conflicts ────────────────────────── tidyverse_conflicts() ──
## x dplyr::filter() masks stats::filter()
## x dplyr::lag()    masks stats::lag()
```

```
library(dplyr)
library(phyloseq)
library(corncob)
library(ggplot2)
library(vegan)
```

```
## Loading required package: permute
```

```
## Loading required package: lattice
```

```
## This is vegan 2.5-6
```

```
library(lmerTest)
```

```
## Loading required package: lme4
```

```
## Loading required package: Matrix
```

```
##
## Attaching package: 'Matrix'
```

```
## The following objects are masked from 'package:tidyr':
##
##     expand, pack, unpack
```

```
##
## Attaching package: 'lmerTest'
```

```
## The following object is masked from 'package:lme4':
##
##     lmer
```

```
## The following object is masked from 'package:stats':
##
##     step
```

Data organization

```
gh.meta <- read.csv("R_files/gh_meta_samples.csv", row.names = 1)
gh.counts <- read.csv("R_files/gh_counts_decontam.csv", row.names = 1)
gh.tax <- read.csv("R_files/gh_tax_decontam.csv", row.names = 1)

gh.counts <- as.data.frame(t(gh.counts))
```

```
str(gh.meta)
```

```
## 'data.frame':    79 obs. of  8 variables:
##  $ type      : chr  "field_swab" "field_swab" "field_swab" "field_swab" ...
##  $ form      : chr  "swab" "swab" "swab" "swab" ...
##  $ note1     : chr  "" "" "urea" "" ...
##  $ toe.clip  : int  101 2 3 4 5 NA 7 8 9 10 ...
##  $ treatment : chr  "Dissected" "Dissected" "Induced" "Dissected" ...
##  $ qubit     : chr  "4.76" "3.88" "0.892" "5.49" ...
##  $ location  : chr  "" "" "" "North Fork" ...
##  $ is.control: logi  FALSE FALSE FALSE FALSE FALSE FALSE ...
```

```
gh.meta$toe.clip <- as.factor(gh.meta$toe.clip)
gh.meta <- gh.meta[order(rownames(gh.meta)),]
gh.counts <- gh.counts[order(rownames(gh.counts)),]

str(gh.meta)
```

```
## 'data.frame':    79 obs. of  8 variables:
##  $ type      : chr  "field_swab" "field_swab" "field_swab" "field_swab" ...
##  $ form      : chr  "swab" "swab" "swab" "swab" ...
##  $ note1     : chr  "" "" "urea" "" ...
##  $ toe.clip  : Factor w/ 28 levels "2","3","4","5",..: 28 1 2 3 4 NA 5 6 7 8 ...
##  $ treatment : chr  "Dissected" "Dissected" "Induced" "Dissected" ...
##  $ qubit     : chr  "4.76" "3.88" "0.892" "5.49" ...
##  $ location  : chr  "" "" "" "North Fork" ...
##  $ is.control: logi  FALSE FALSE FALSE FALSE FALSE FALSE ...
```

Remove females that did not contribute eggs to the study

```
remove <- c("8", "10", "12","14","15","16" ,"27", NA)
gh.meta <- gh.meta[!(gh.meta$toe.clip %in% remove),]
gh.counts <- gh.counts[rownames(gh.counts) %in% rownames(gh.meta),]
```

```
gh.counts <- gh.counts[,colSums(gh.counts) > 10]
gh.tax <- gh.tax[rownames(gh.tax) %in% colnames(gh.counts),]

gh.counts.ps <- otu_table(gh.counts, taxa_are_rows = FALSE)
colnames(gh.counts.ps) <- colnames(gh.counts)

gh.tax.ps <- tax_table(gh.tax)
```

```
## Warning in .local(object): Coercing from data.frame class to character matrix
## prior to building taxonomyTable.
## This could introduce artifacts.
## Check your taxonomyTable, or coerce to matrix manually.
```

```
taxa_names(gh.tax.ps) <- taxa_names(gh.counts.ps)
colnames(gh.tax.ps) <- c("Kingdom","Phylum","Class","Order","Family","Genus")
gh.meta.ps <- sample_data(gh.meta)


gh.ps <- phyloseq(gh.counts.ps, gh.tax.ps, gh.meta.ps)
```

```
richness <- estimate_richness(gh.ps, measures = "Observed")
gh.meta <- cbind(gh.meta, richness)
```

```
gh.ps@otu_table <- transform_sample_counts(gh.ps@otu_table,
                                           function(x)  log10(x + 1))
```

```
shannon <- estimate_richness(gh.ps, measures = "Shannon")
gh.meta <- cbind(gh.meta, shannon)
gh.meta.ps <- sample_data(gh.meta)
gh.ps@sam_data <- gh.meta.ps
```

Cloacal Swab Analysis

Narrow down to just swabs, and just animal for which we have field and lab swabs (some lab swabs were opened for a different project before they could be sequenced)

```
swab.meta <- gh.meta[gh.meta$form == "swab",]
lab.tc <- swab.meta$toe.clip[swab.meta$type == "lab_swab"]
swab.pair.meta <- swab.meta[swab.meta$toe.clip %in% lab.tc,]
```

Alpha Diversity

Did cloacal diversity change due to shipment to the lab?

Assumption check – Shannon diversity

```
shipping.lm <- lm(Shannon ~ type, data = swab.pair.meta)

densityplot(~residuals(shipping.lm), group = type, data = swab.pair.meta, auto.key = TRUE)
```

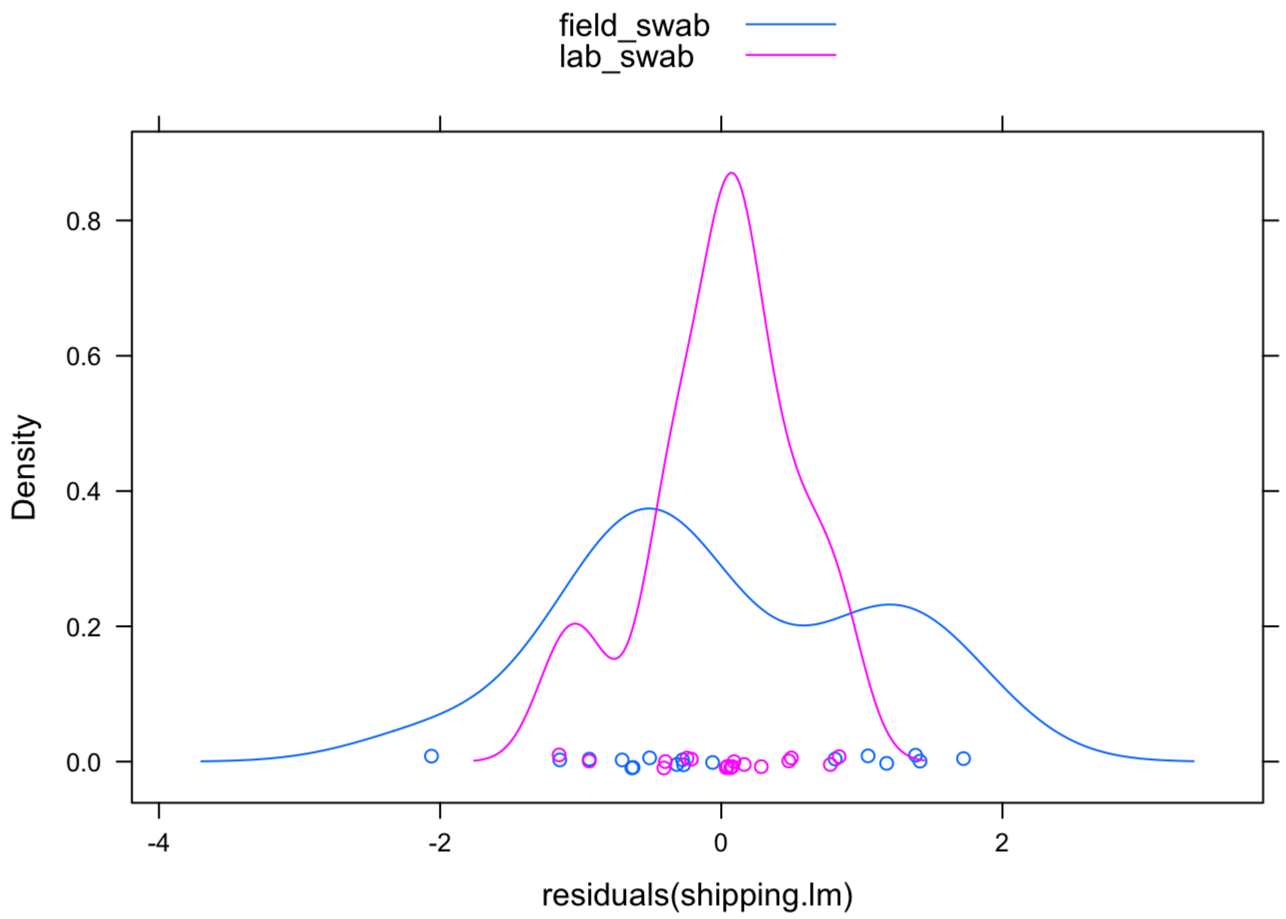


```
with(swab.pair.meta, shapiro.test(Shannon[type == "field_swab"]))
```

```
##
##  Shapiro-Wilk normality test
##
## data:  Shannon[type == "field_swab"]
## W = 0.93469, p-value = 0.2605
```

```
with(swab.pair.meta, shapiro.test(Shannon[type ==  "lab_swab"]))
```

```
##
##  Shapiro-Wilk normality test
##
## data:  Shannon[type == "lab_swab"]
## W = 0.95272, p-value = 0.5009
```

```
shipping.t <- t.test(Shannon ~ type, data = swab.pair.meta, var.equal = TRUE, paired = TRUE)
shipping.t
```

```
##
##  Paired t-test
##
## data:  Shannon by type
## t = 1.4904, df = 16, p-value = 0.1556
## alternative hypothesis: true difference in means is not equal to 0
## 95 percent confidence interval:
##  -0.1590803  0.9124330
## sample estimates:
## mean of the differences
##               0.3766764
```

Check with var.equal = false just in case it affects conclusions

```
shipping.t.nov <- t.test(Shannon ~ type, data = swab.pair.meta, var.equal = FALSE, paired = TRUE
)
shipping.t.nov
```

```
##
##  Paired t-test
##
## data:  Shannon by type
## t = 1.4904, df = 16, p-value = 0.1556
## alternative hypothesis: true difference in means is not equal to 0
## 95 percent confidence interval:
##  -0.1590803  0.9124330
## sample estimates:
## mean of the differences
##               0.3766764
```

Assumption Check – Richness

```
shipping.lm.rich <- lm(Observed ~ type, data = swab.pair.meta)

densityplot(~residuals(shipping.lm.rich), group = type, data = swab.pair.meta, auto.key = TRUE)
```

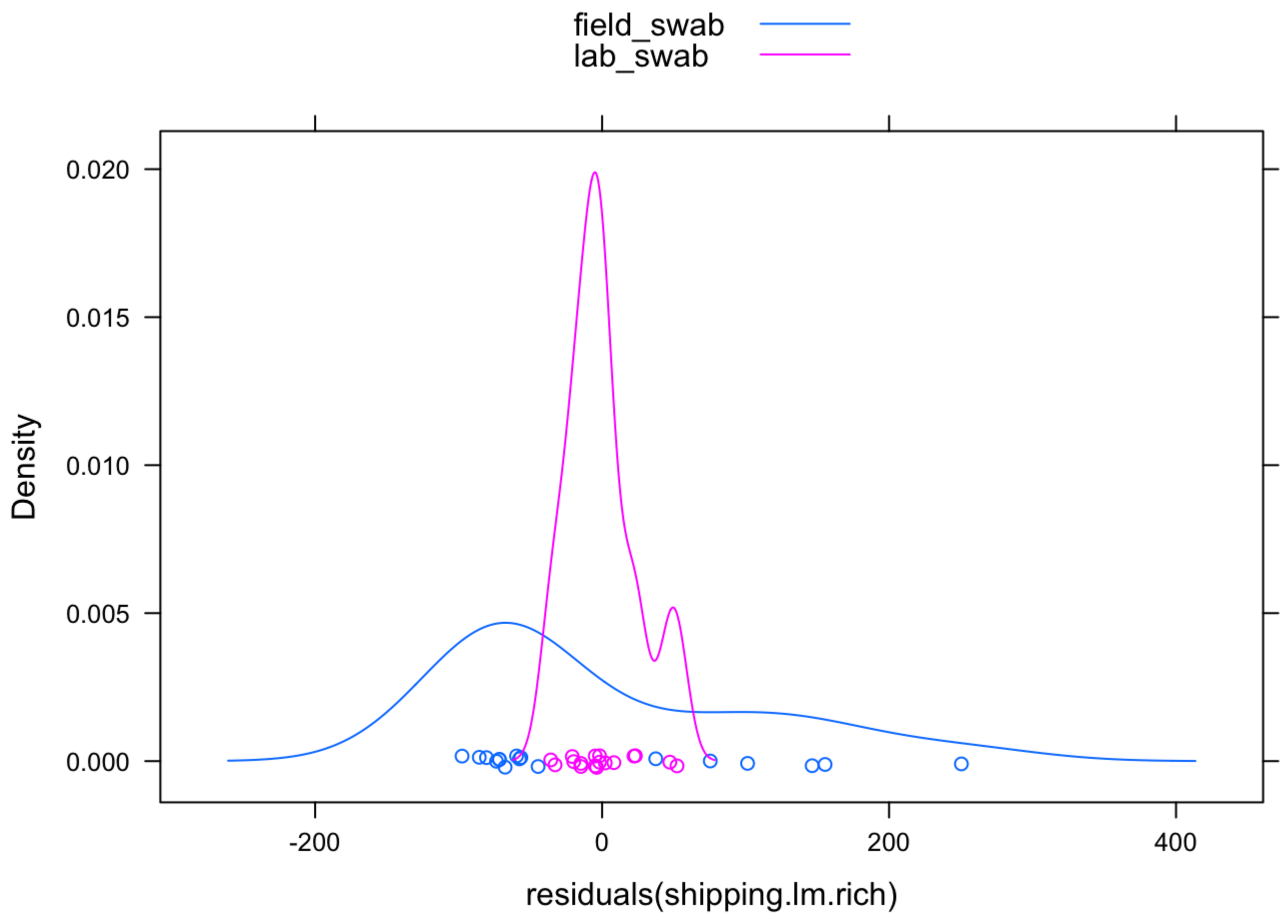


```
with(swab.pair.meta, shapiro.test(Observed[type == "field_swab"]))
```

```
##
##  Shapiro-Wilk normality test
##
## data:  Observed[type == "field_swab"]
## W = 0.79828, p-value = 0.001922
```

```
with(swab.pair.meta, shapiro.test(Observed[type ==  "lab_swab"]))
```

```
##
##  Shapiro-Wilk normality test
##
## data:  Observed[type == "lab_swab"]
## W = 0.92916, p-value = 0.2107
```

Log transform

```
shipping.lm.rich.log <- lm(log10(Observed) ~ type, data = swab.pair.meta)

densityplot(~residuals(shipping.lm.rich.log), group = type, data = swab.pair.meta, auto.key = TR
UE)
```

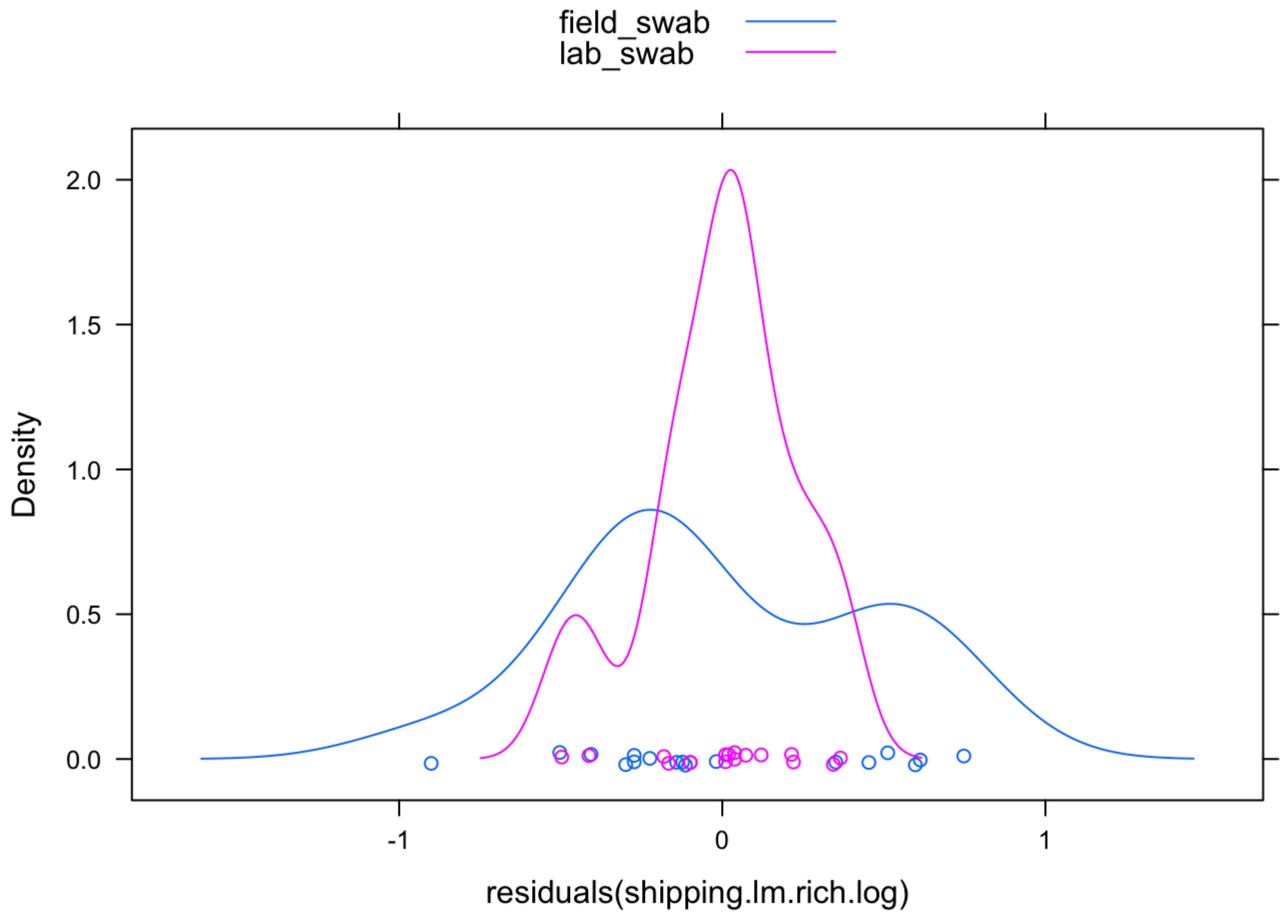


```
with(swab.pair.meta, shapiro.test(log10(Observed)[type == "field_swab"]))
```

```
##
##  Shapiro-Wilk normality test
##
## data:  log10(Observed)[type == "field_swab"]
## W = 0.93516, p-value = 0.2652
```

```
with(swab.pair.meta, shapiro.test(log10(Observed)[type ==  "lab_swab"]))
```

```
##
##  Shapiro-Wilk normality test
##
## data:  log10(Observed)[type == "lab_swab"]
## W = 0.95367, p-value = 0.517
```

```
shipping.rich.t <- t.test(log10(Observed) ~ type, paired = TRUE, var.equal = TRUE, data = swab.p
air.meta)
shipping.rich.t
```

```
##
##  Paired t-test
##
## data:  log10(Observed) by type
## t = 1.4649, df = 16, p-value = 0.1623
## alternative hypothesis: true difference in means is not equal to 0
## 95 percent confidence interval:
##  -0.07197165  0.39392414
## sample estimates:
## mean of the differences
##               0.1609762
```

Is there a difference between treatment groups on egg acquisition day?

We will only use the lab swabs for this analysis because they represent the community on day of egg acquisition

```
lab.swabs <- swab.meta[swab.meta$type == "lab_swab",]
```

Assumption Chek – Shannon Diverstiy

```
swab.treat.lm <- lm(Shannon ~ treatment, data = lab.swabs)

densityplot(~residuals(swab.treat.lm), group = treatment, data = lab.swabs, auto.key = TRUE)
```

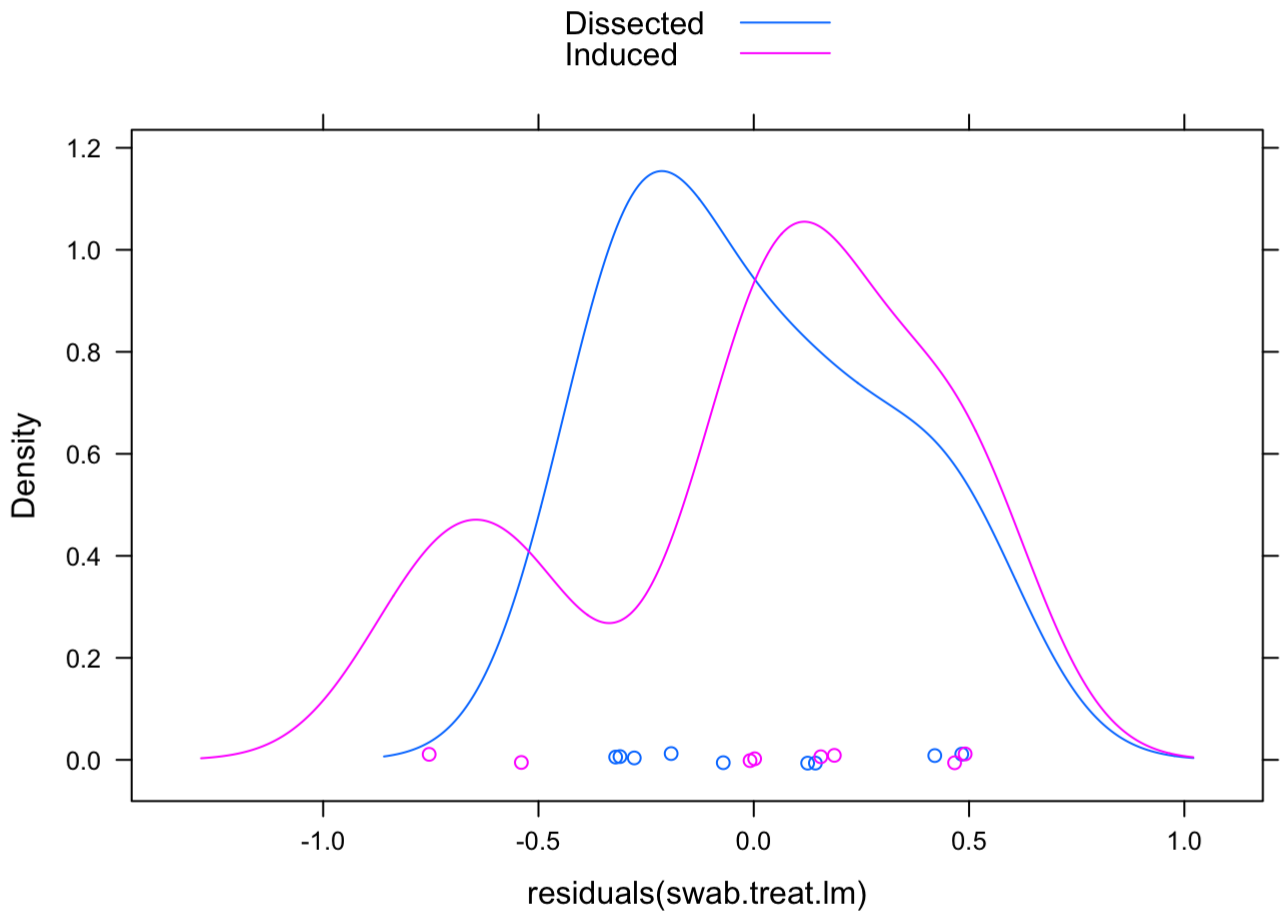


```
with(lab.swabs, shapiro.test(Shannon[treatment == "Dissected"]))
```

```
##
##  Shapiro-Wilk normality test
##
## data:  Shannon[treatment == "Dissected"]
## W = 0.88766, p-value = 0.1888
```

```
with(lab.swabs, shapiro.test(Shannon[treatment == "Induced"]))
```

```
##
##  Shapiro-Wilk normality test
##
## data:  Shannon[treatment == "Induced"]
## W = 0.90151, p-value = 0.2981
```

```
swab.treat.t <- t.test(Shannon ~ treatment, var.equal = TRUE, data = lab.swabs)
swab.treat.t
```

```
##
##  Two Sample t-test
##
## data:  Shannon by treatment
## t = 4.1107, df = 15, p-value = 0.000926
## alternative hypothesis: true difference in means is not equal to 0
## 95 percent confidence interval:
##  0.3634408 1.1462195
## sample estimates:
## mean in group Dissected   mean in group Induced
##                4.116234                3.361404
```

Assumption Check – Richness

```
swab.treat.rich.lm <- lm(Observed ~ treatment, data = lab.swabs)

densityplot(~residuals(swab.treat.rich.lm), group = treatment, data = lab.swabs, auto.key = TRUE
)
```

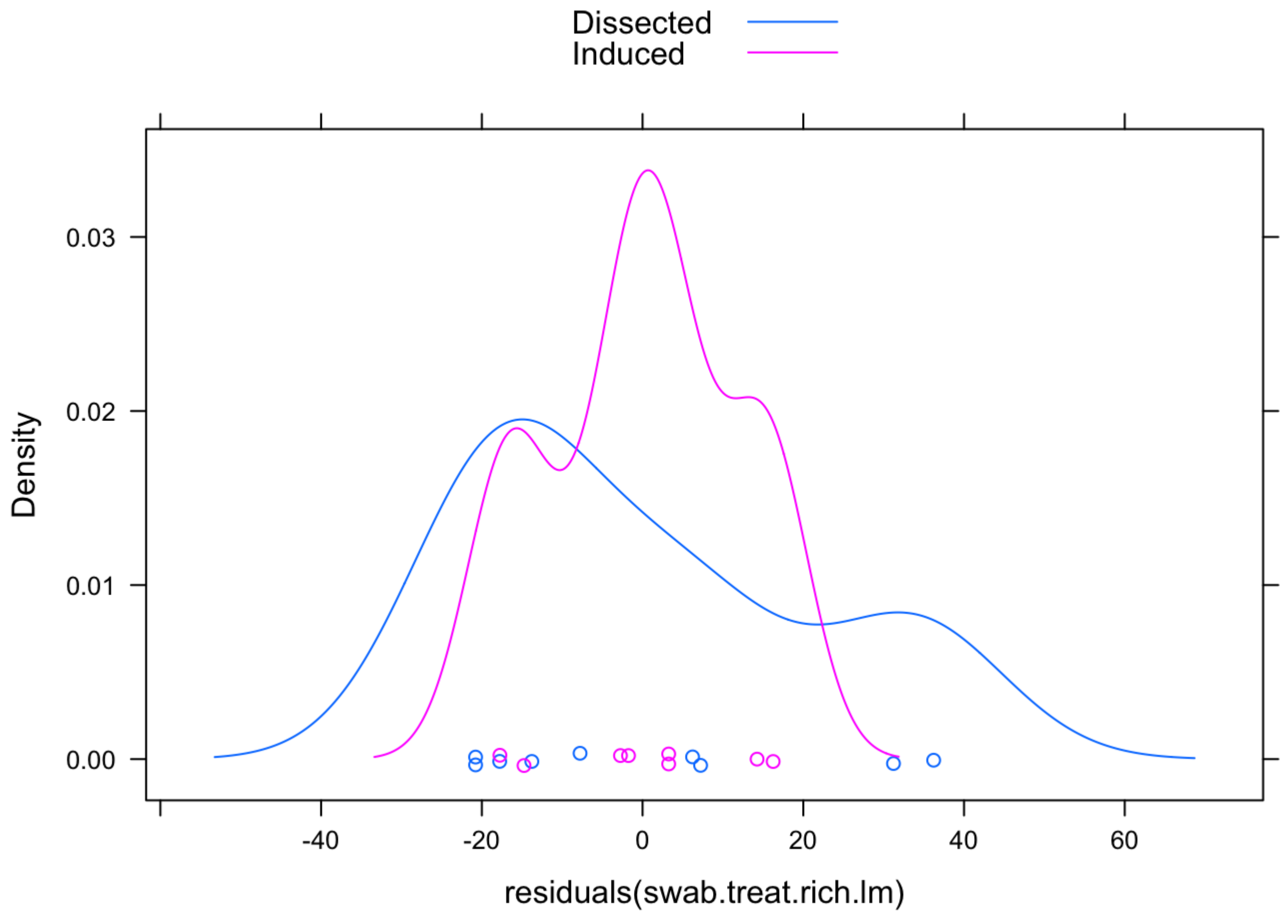


```
with(lab.swabs, shapiro.test(Observed[treatment == "Dissected"]))
```

```
##
##  Shapiro-Wilk normality test
##
## data:  Observed[treatment == "Dissected"]
## W = 0.8615, p-value = 0.09954
```

```
with(lab.swabs, shapiro.test(Observed[treatment == "Induced"]))
```

```
##
##  Shapiro-Wilk normality test
##
## data:  Observed[treatment == "Induced"]
## W = 0.93634, p-value = 0.5755
```

```
swab.treat.rich.t <- t.test(Observed ~ treatment, var.equal = TRUE, data = lab.swabs)
swab.treat.rich.t
```

```
##
##  Two Sample t-test
##
## data:  Observed by treatment
## t = 3.8989, df = 15, p-value = 0.001424
## alternative hypothesis: true difference in means is not equal to 0
## 95 percent confidence interval:
##  15.42542 52.63013
## sample estimates:
## mean in group Dissected   mean in group Induced
##                65.77778                31.75000
```

Diversity is higher in both metrics for dissected samples

Beta Diversity

Is there a difference in composition due to shipment?

```
swab.ps <- subset_samples(gh.ps, sample_names(gh.ps) %in% rownames(swab.meta))
```

```
swab.dist <- vegdist(swab.ps@otu_table, method = "bray")

anova(betadisper(swab.dist, swab.ps@sam_data[["type"]]))
```

```
## Analysis of Variance Table
##
## Response: Distances
##           Df  Sum Sq  Mean Sq F value Pr(>F)
## Groups     1 0.02745 0.027454  1.3931 0.2456
## Residuals 36 0.70946 0.019707
```

```
set.seed(1)
swab.perma <- adonis(swab.dist ~ type, data = swab.meta)
swab.perma
```

```
##
## Call:
## adonis(formula = swab.dist ~ type, data = swab.meta)
##
## Permutation: free
## Number of permutations: 999
##
## Terms added sequentially (first to last)
##
##           Df SumsOfSqs MeanSqs F.Model      R2 Pr(>F)
## type       1    0.3135 0.31345 0.90705 0.02458  0.472
## Residuals 36   12.4406 0.34557         0.97542
## Total     37   12.7541                 1.00000
```

Is there a difference in composition between treatment groups on day of egg acquisition?

Subset down to just the lab swabs, as that was the composition at the time of treatment

```
lab.swab.ps <- subset_samples(swab.ps, type == "lab_swab")
```

```
lab.swab.dist <- vegdist(lab.swab.ps@otu_table, method = "bray")

anova(betadisper(lab.swab.dist, lab.swab.ps@sam_data[["treatment"]]))
```

```
## Analysis of Variance Table
##
## Response: Distances
##           Df   Sum Sq   Mean Sq F value Pr(>F)
## Groups     1 0.002711 0.0027111   0.137 0.7164
## Residuals 15 0.296760 0.0197840
```

```
set.seed(1)
lab.swab.perma <- adonis(lab.swab.dist ~ treatment, data = lab.swabs)
lab.swab.perma
```

```
##
## Call:
## adonis(formula = lab.swab.dist ~ treatment, data = lab.swabs)
##
## Permutation: free
## Number of permutations: 999
##
## Terms added sequentially (first to last)
##
##           Df SumsOfSqs MeanSqs F.Model      R2 Pr(>F)
## treatment  1    0.5026 0.50259  1.6922 0.10138  0.161
## Residuals 15    4.4551 0.29701         0.89862
## Total     16    4.9577                 1.00000
```

Shell Analysis

Narrow down to only shell samples

```
shell.meta <- gh.meta[gh.meta$form == "shell",]
shell.counts <- gh.counts[rownames(gh.counts) %in% rownames(shell.meta),]
shell.ps <- subset_samples(gh.ps, form == "shell")
```

We generally don’t care about the effect of time, because we expected bacterial communities to grow during incubation. Samples were split up by day, which also simplifies analyses.

```
d0.meta <- shell.meta[shell.meta$type == "Day0",]

d25.meta <- shell.meta[shell.meta$type == "Day25",]
```

Alpha Diversity

Day 0

Assumption Check – Richness

```
d0.r <- lm(Observed ~ treatment, data = d0.meta)

densityplot(~ residuals(d0.r), group = treatment, data = d0.meta, auto.key=TRUE)
```

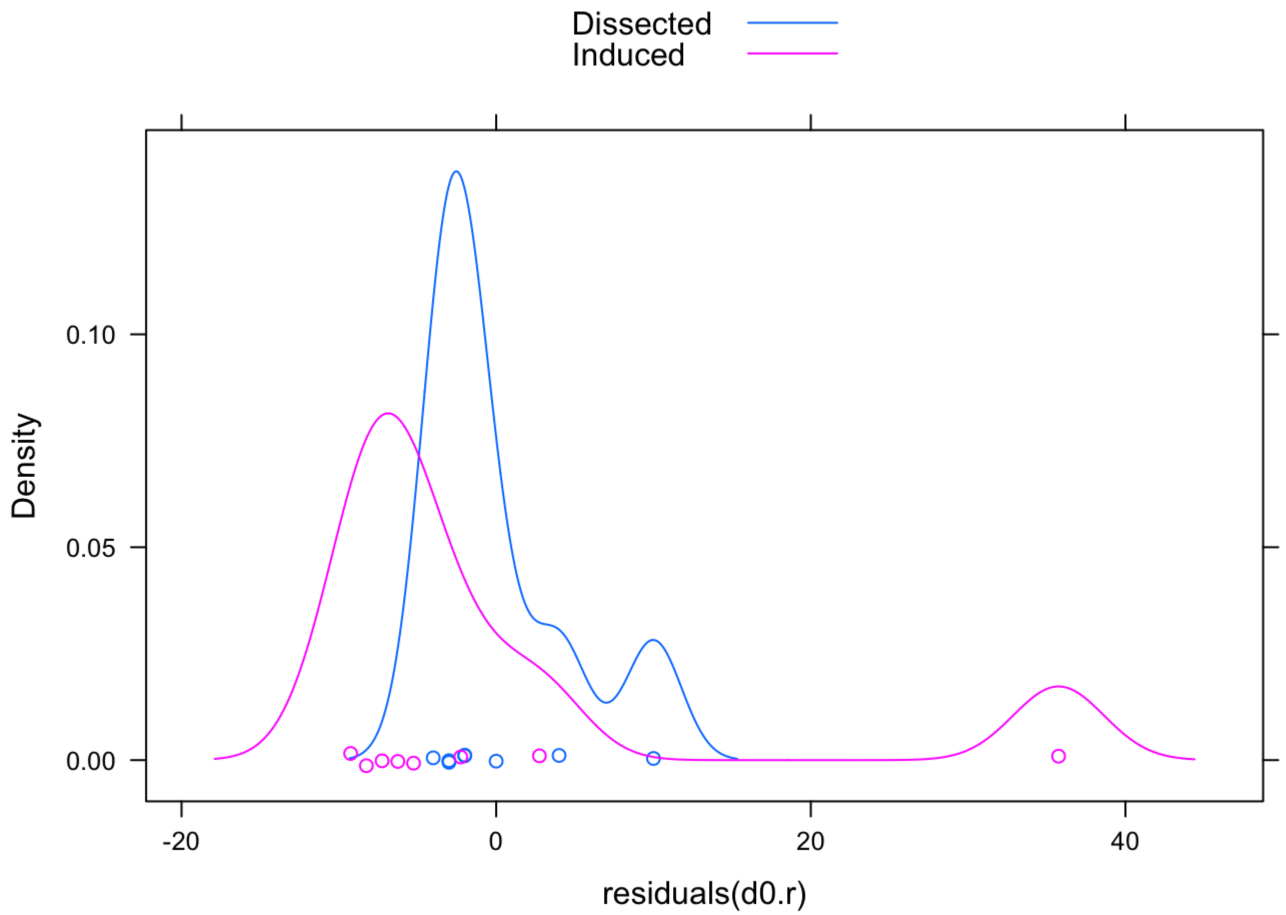


```
with(d0.meta, shapiro.test(Observed[treatment == "Dissected"]))
```

```
##
##  Shapiro-Wilk normality test
##
## data:  Observed[treatment == "Dissected"]
## W = 0.79539, p-value = 0.02554
```

```
with(d0.meta, shapiro.test(Observed[treatment == "Induced"]))
```

```
##
##  Shapiro-Wilk normality test
##
## data:  Observed[treatment == "Induced"]
## W = 0.64025, p-value = 0.0004671
```

Log transform

```
d0.rL <- lm(log10(Observed) ~ treatment, data = d0.meta)

densityplot(~ residuals(d0.rL), group = treatment, data = d0.meta, auto.key=TRUE)
```

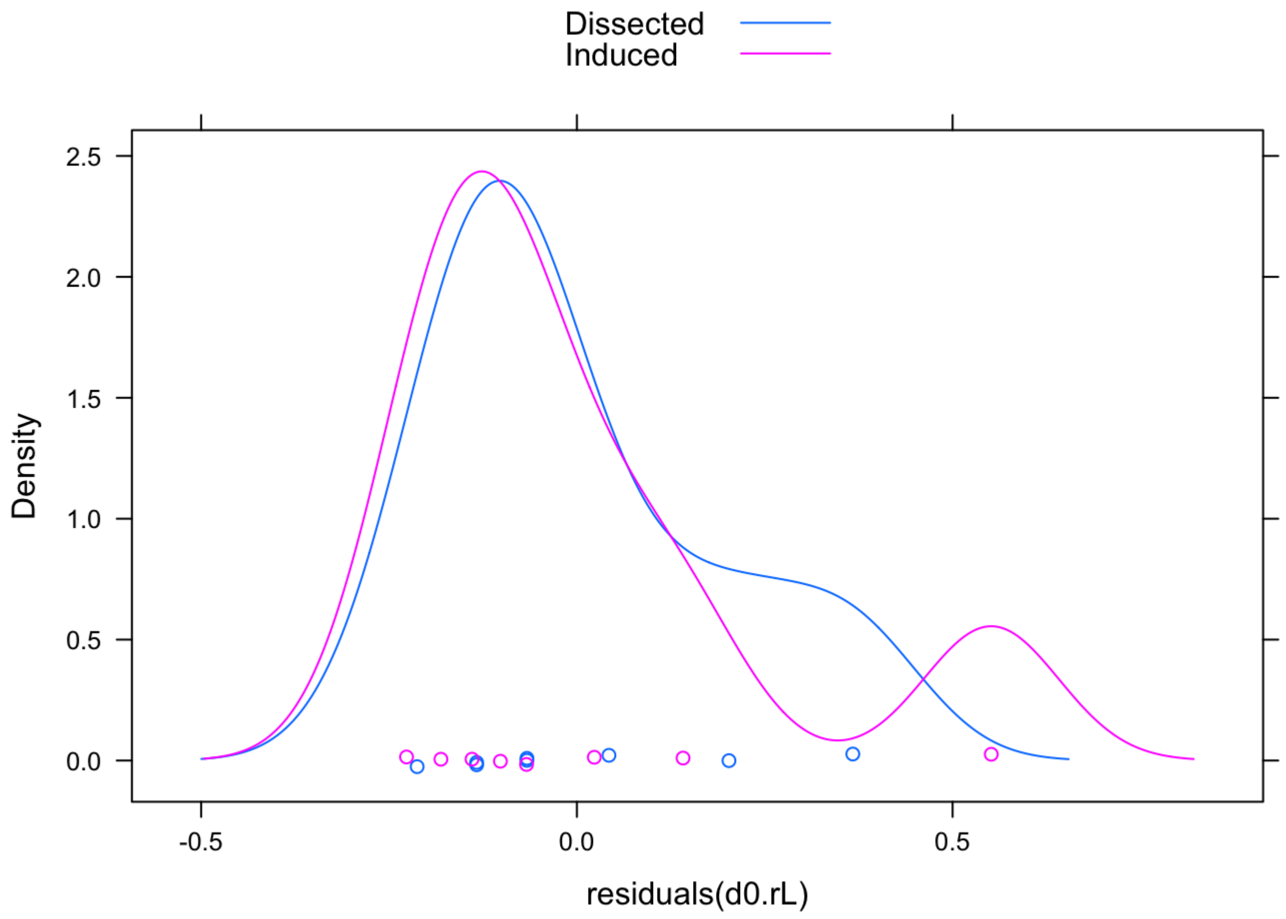


```
with(d0.meta, shapiro.test(log10(Observed)[treatment == "Dissected"]))
```

```
##
##  Shapiro-Wilk normality test
##
## data:  log10(Observed)[treatment == "Dissected"]
## W = 0.89059, p-value = 0.237
```

```
with(d0.meta, shapiro.test(log10(Observed)[treatment == "Induced"]))
```

```
##
##  Shapiro-Wilk normality test
##
## data:  log10(Observed)[treatment == "Induced"]
## W = 0.82126, p-value = 0.04811
```

```
d0.r.t <- t.test(log10(Observed) ~ treatment, data = d0.meta, var.equal = TRUE)
d0.r.t
```

```
##
##  Two Sample t-test
##
## data:  log10(Observed) by treatment
## t = -2.392, df = 14, p-value = 0.03135
## alternative hypothesis: true difference in means is not equal to 0
## 95 percent confidence interval:
##  -0.5109349 -0.0278363
## sample estimates:
## mean in group Dissected   mean in group Induced
##                0.911551                1.180937
```

Assumption check – Shannon Diversity

```
d0.shan <- lm(Shannon ~ treatment, data = d0.meta)

densityplot(~ residuals(d0.shan), group = treatment, data = d0.meta, auto.key=TRUE)
```

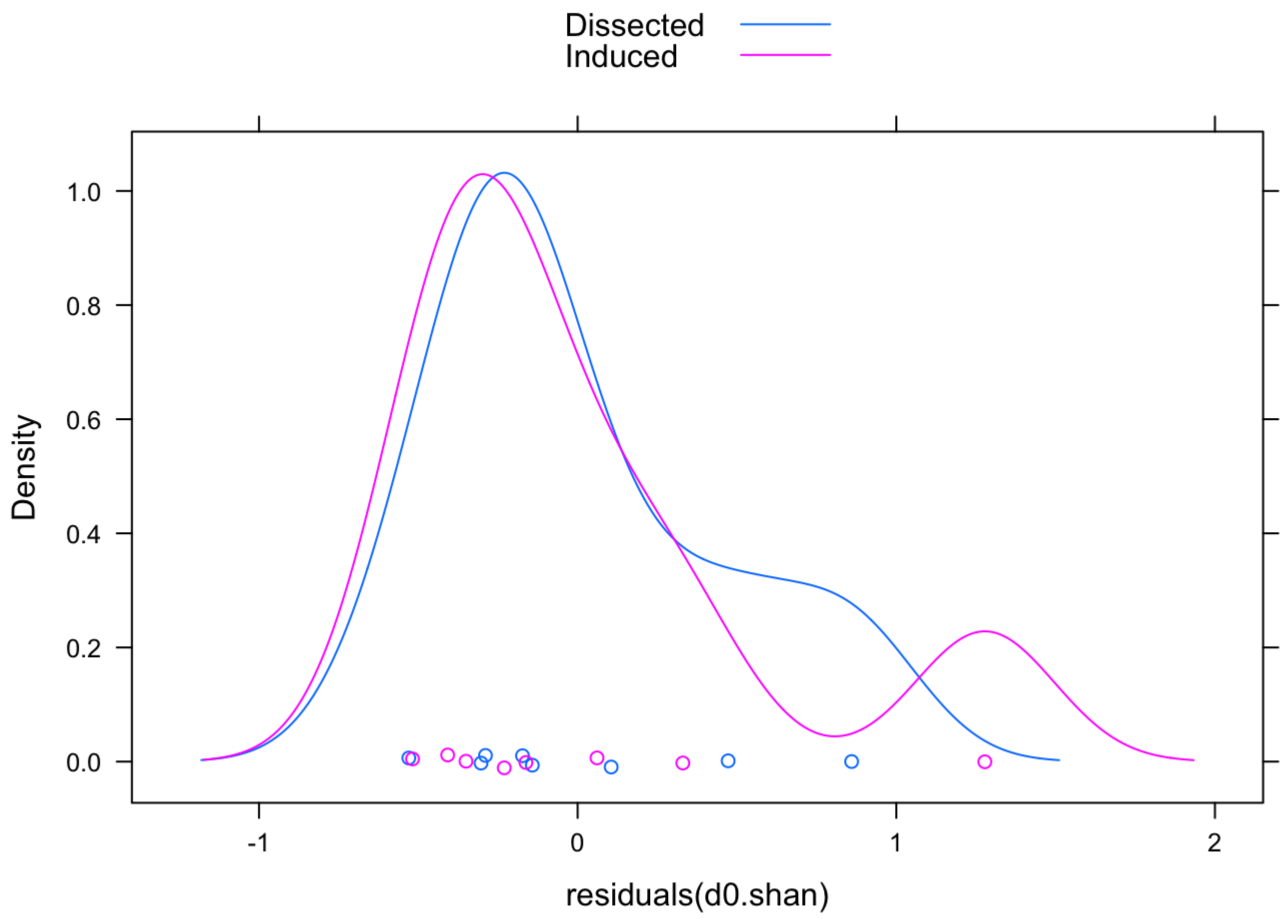


```
with(d0.meta, shapiro.test(Shannon[treatment == "Dissected"]))
```

```
##
##  Shapiro-Wilk normality test
##
## data:  Shannon[treatment == "Dissected"]
## W = 0.90443, p-value = 0.3165
```

```
with(d0.meta, shapiro.test(Shannon[treatment == "Induced"]))
```

```
##
##  Shapiro-Wilk normality test
##
## data:  Shannon[treatment == "Induced"]
## W = 0.81788, p-value = 0.04432
```

Log transform

```
d0.shanL <- lm(log10(Shannon) ~ treatment, data = d0.meta)

densityplot(~ residuals(d0.shanL), group = treatment, data = d0.meta, auto.key=TRUE)
```

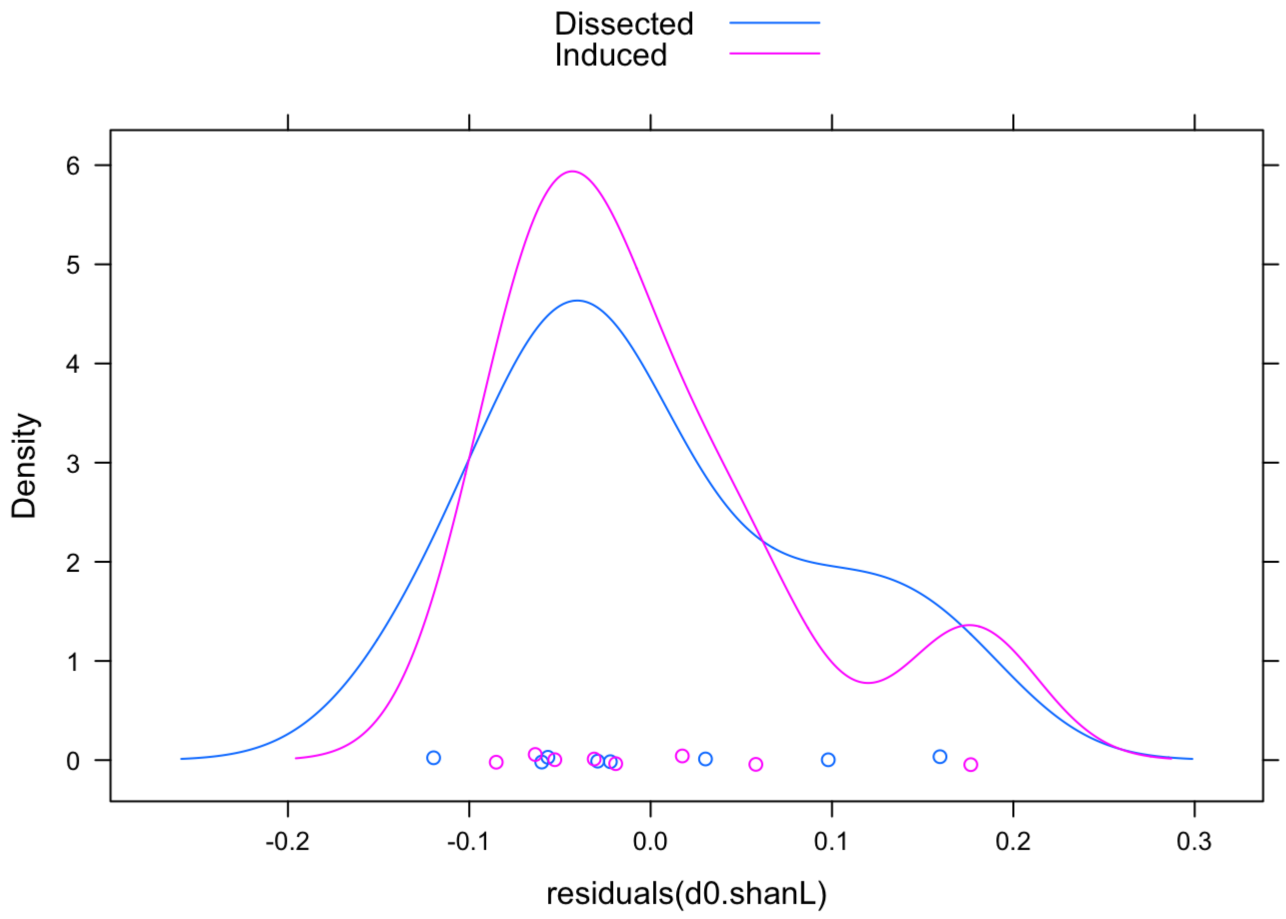


```
with(d0.meta, shapiro.test(log10(Shannon)[treatment == "Dissected"]))
```

```
##
##  Shapiro-Wilk normality test
##
## data:  log10(Shannon)[treatment == "Dissected"]
## W = 0.94111, p-value = 0.6221
```

```
with(d0.meta, shapiro.test(log10(Shannon)[treatment == "Induced"]))
```

```
##
##  Shapiro-Wilk normality test
##
## data:  log10(Shannon)[treatment == "Induced"]
## W = 0.87558, p-value = 0.1708
```

```
d0.S.t <- t.test(log10(Shannon) ~ treatment, data = d0.meta, var.equal = TRUE)
d0.S.t
```

```
##
##  Two Sample t-test
##
## data:  log10(Shannon) by treatment
## t = -2.6036, df = 14, p-value = 0.02083
## alternative hypothesis: true difference in means is not equal to 0
## 95 percent confidence interval:
##  -0.20921453 -0.02021464
## sample estimates:
## mean in group Dissected   mean in group Induced
##               0.3073836               0.4220982
```

Day 25

Assumption Check – Richness

```
d25.r <- lm(Observed ~ treatment, data = d25.meta)

densityplot(~ residuals(d25.r), group = treatment, data = d25.meta, auto.key=TRUE)
```

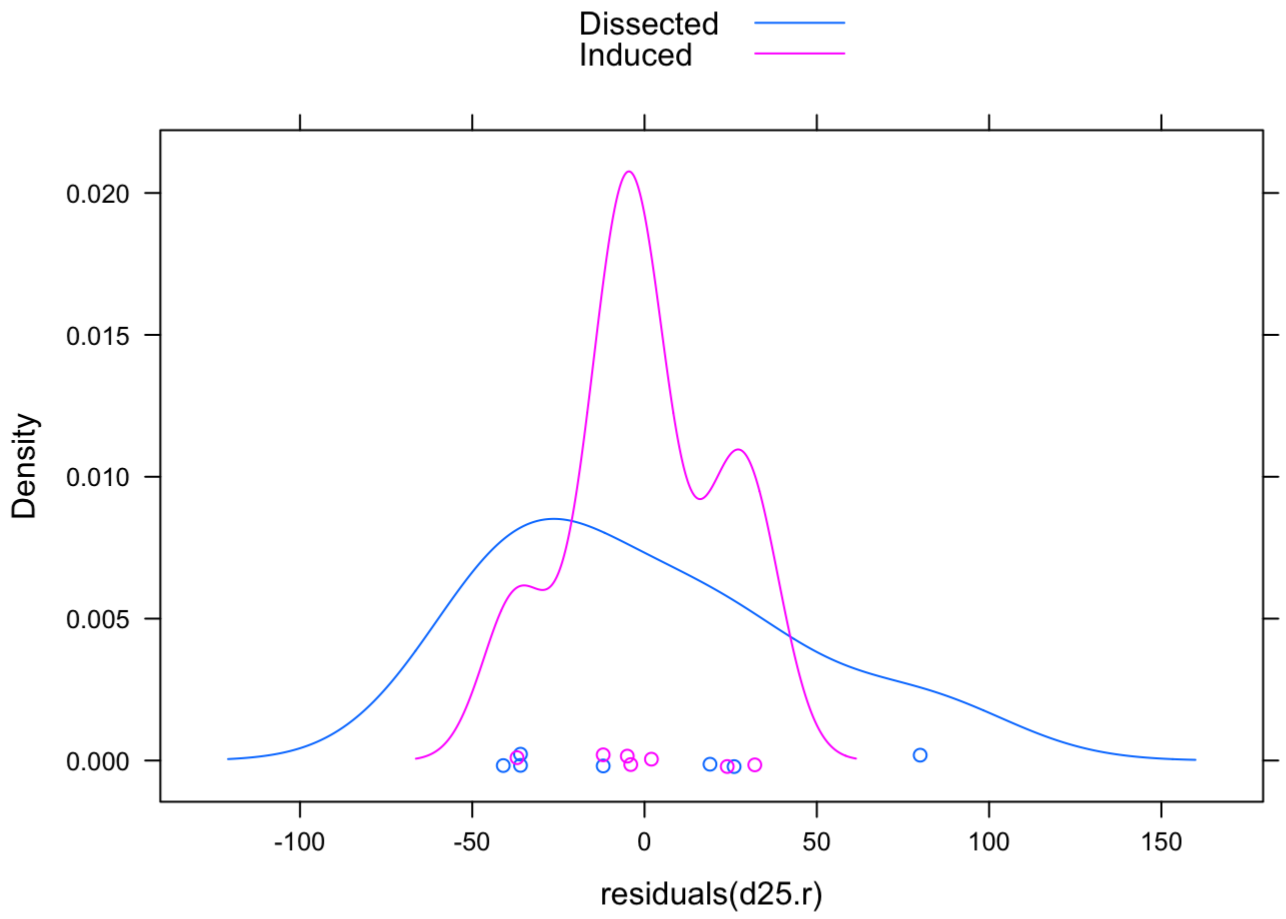


```
with(d25.meta, shapiro.test(Observed[treatment == "Dissected"]))
```

```
##
##  Shapiro-Wilk normality test
##
## data:  Observed[treatment == "Dissected"]
## W = 0.87673, p-value = 0.2123
```

```
with(d25.meta, shapiro.test(Observed[treatment == "Induced"]))
```

```
##
##  Shapiro-Wilk normality test
##
## data:  Observed[treatment == "Induced"]
## W = 0.95409, p-value = 0.7667
```

```
d25.r.t <- t.test(Observed ~ treatment, data = d25.meta, var.equal = TRUE)
d25.r.t
```

```
##
##  Two Sample t-test
##
## data:  Observed by treatment
## t = -0.052894, df = 12, p-value = 0.9587
## alternative hypothesis: true difference in means is not equal to 0
## 95 percent confidence interval:
##  -42.19216  40.19216
## sample estimates:
## mean in group Dissected   mean in group Induced
##                      46                      47
```

Assumption Check – Shannon Diversity

```
d25.shan <- lm(Shannon ~ treatment, data = d25.meta)

densityplot(~ residuals(d25.shan), group = treatment, data = d25.meta, auto.key=TRUE)
```

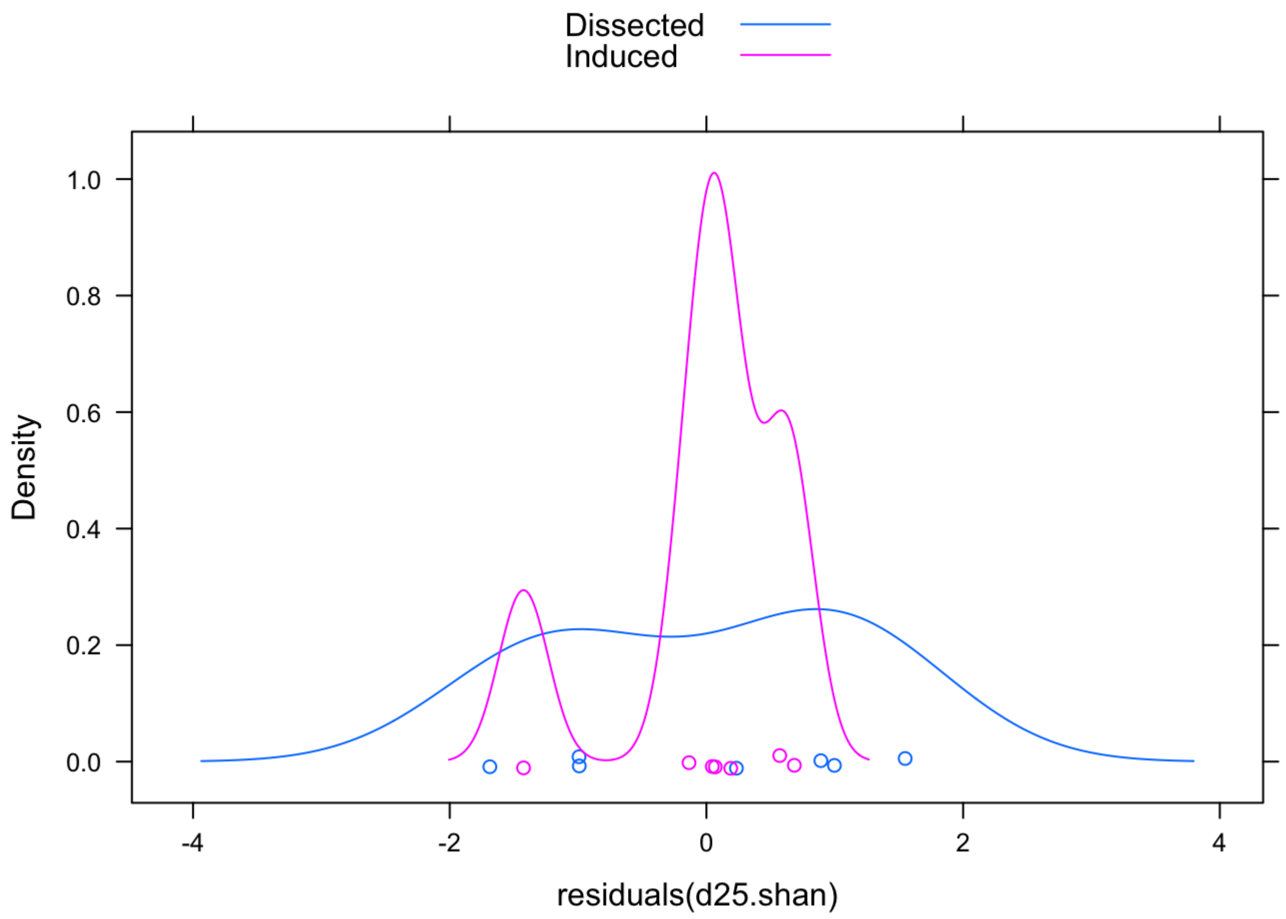


```
with(d25.meta, shapiro.test(Shannon[treatment == "Dissected"]))
```

```
##
##  Shapiro-Wilk normality test
##
## data:  Shannon[treatment == "Dissected"]
## W = 0.91905, p-value = 0.462
```

```
with(d25.meta, shapiro.test(Shannon[treatment == "Induced"]))
```

```
##
##  Shapiro-Wilk normality test
##
## data:  Shannon[treatment == "Induced"]
## W = 0.83126, p-value = 0.08224
```

Log transform

```
d25.shanL <- lm(log10(Shannon) ~ treatment, data = d25.meta)

densityplot(~ residuals(d25.shanL), group = treatment, data = d25.meta, auto.key=TRUE)
```

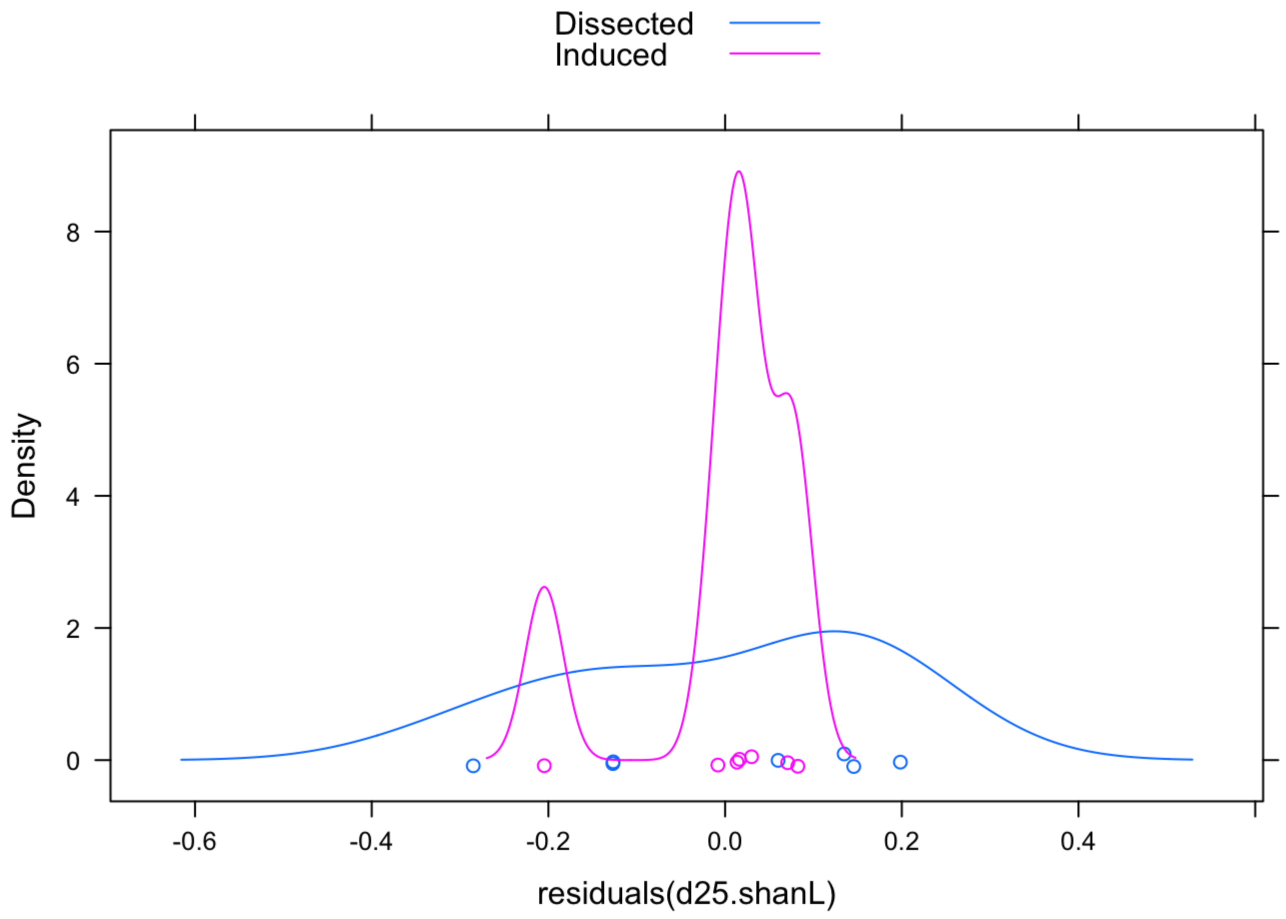


```
with(d25.meta, shapiro.test(log10(Shannon)[treatment == "Dissected"]))
```

```
##
##  Shapiro-Wilk normality test
##
## data:  log10(Shannon)[treatment == "Dissected"]
## W = 0.90432, p-value = 0.358
```

```
with(d25.meta, shapiro.test(log10(Shannon)[treatment == "Induced"]))
```

```
##
##  Shapiro-Wilk normality test
##
## data:  log10(Shannon)[treatment == "Induced"]
## W = 0.7634, p-value = 0.01748
```

Going to use a non-parametric test

```
d25.S.wilcox <- wilcox.test(Shannon ~ treatment, data = d25.meta)
d25.S.wilcox
```

```
##
##  Wilcoxon rank sum exact test
##
## data:  Shannon by treatment
## W = 21, p-value = 0.7104
## alternative hypothesis: true location shift is not equal to 0
```

Beta Diversity

```
d0.ps <- subset_samples(shell.ps, type == "Day0")
d25.ps <- subset_samples(shell.ps, type == "Day25")
```

Day 0

Dispersion

```
d0.dist <- vegdist(d0.ps@otu_table, method = "bray")

anova(betadisper(d0.dist, d0.ps@sam_data[["treatment"]]))
```

```
## Analysis of Variance Table
##
## Response: Distances
##           Df    Sum Sq    Mean Sq F value Pr(>F)
## Groups     1 0.0002765 0.00027651  0.2264 0.6416
## Residuals 14 0.0171024 0.00122160
```

Composition

```
set.seed(1)
d0.perma <- adonis(d0.dist ~ treatment, data = d0.meta)
d0.perma
```

```
##
## Call:
## adonis(formula = d0.dist ~ treatment, data = d0.meta)
##
## Permutation: free
## Number of permutations: 999
##
## Terms added sequentially (first to last)
##
##           Df SumsOfSqs MeanSqs F.Model    R2 Pr(>F)
## treatment  1    0.3571 0.35711 0.75244 0.051   0.95
## Residuals 14    6.6444 0.47460         0.949
## Total     15    7.0015                 1.000
```

Day 25

Dispersion

```
d25.dist <- vegdist(d25.ps@otu_table, method = "bray")

anova(betadisper(d25.dist, d25.ps@sam_data[["treatment"]]))
```

```
## Analysis of Variance Table
##
## Response: Distances
##           Df   Sum Sq   Mean Sq F value Pr(>F)
## Groups     1 0.006969 0.0069693  0.4859  0.499
## Residuals 12 0.172100 0.0143417
```

Composition

```
set.seed(1)
d25.perma <- adonis(d25.dist ~ treatment, data = d25.meta)
d25.perma
```

```
##
## Call:
## adonis(formula = d25.dist ~ treatment, data = d25.meta)
##
## Permutation: free
## Number of permutations: 999
##
## Terms added sequentially (first to last)
##
##           Df SumsOfSqs MeanSqs F.Model      R2 Pr(>F)
## treatment  1    0.5938 0.59382  1.4031 0.10468  0.067 .
## Residuals 12    5.0786 0.42322         0.89532
## Total     13    5.6724                 1.00000
## ---
## Signif. codes:  0 '***' 0.001 '**' 0.01 '*' 0.05 '.' 0.1 ' ' 1
```

Swab/Shell Correlation

Correlation of diversity of cloacal swabs to egg shells on Day 0, of dissected and induced females

Induced females

Organizing to just induced females for which we have swabs and viable eggs

```
d0.all <- gh.meta[gh.meta$type == "Day0" | gh.meta$type == "lab_swab",]
```

```
d0.shell.tc <- d0.all$toe.clip[d0.all$type == "Day0"]
d0.swab.tc <- d0.all$toe.clip[d0.all$type == "lab_swab"]

d0.pair <- d0.all[d0.all$toe.clip %in% d0.shell.tc,]
d0.pair <- d0.pair[d0.pair$toe.clip %in% d0.swab.tc,]

d0.ind.pair <- d0.pair[d0.pair$treatment == "Induced",]
```

Shannon Diversity

```
d0.tc<- select(d0.ind.pair, toe.clip, type, Shannon)

d0.tc <- d0.tc %>%
  spread(key = type, value = Shannon)%>%
  group_by(toe.clip)
```

We are also excluding toe clip 9, she died during inducing and she was an outlier in this analysis.

```
d0.tc <- d0.tc[d0.tc$toe.clip != "9",]
```

Assumption Check

```
plot(density(d0.tc$Day0))
```

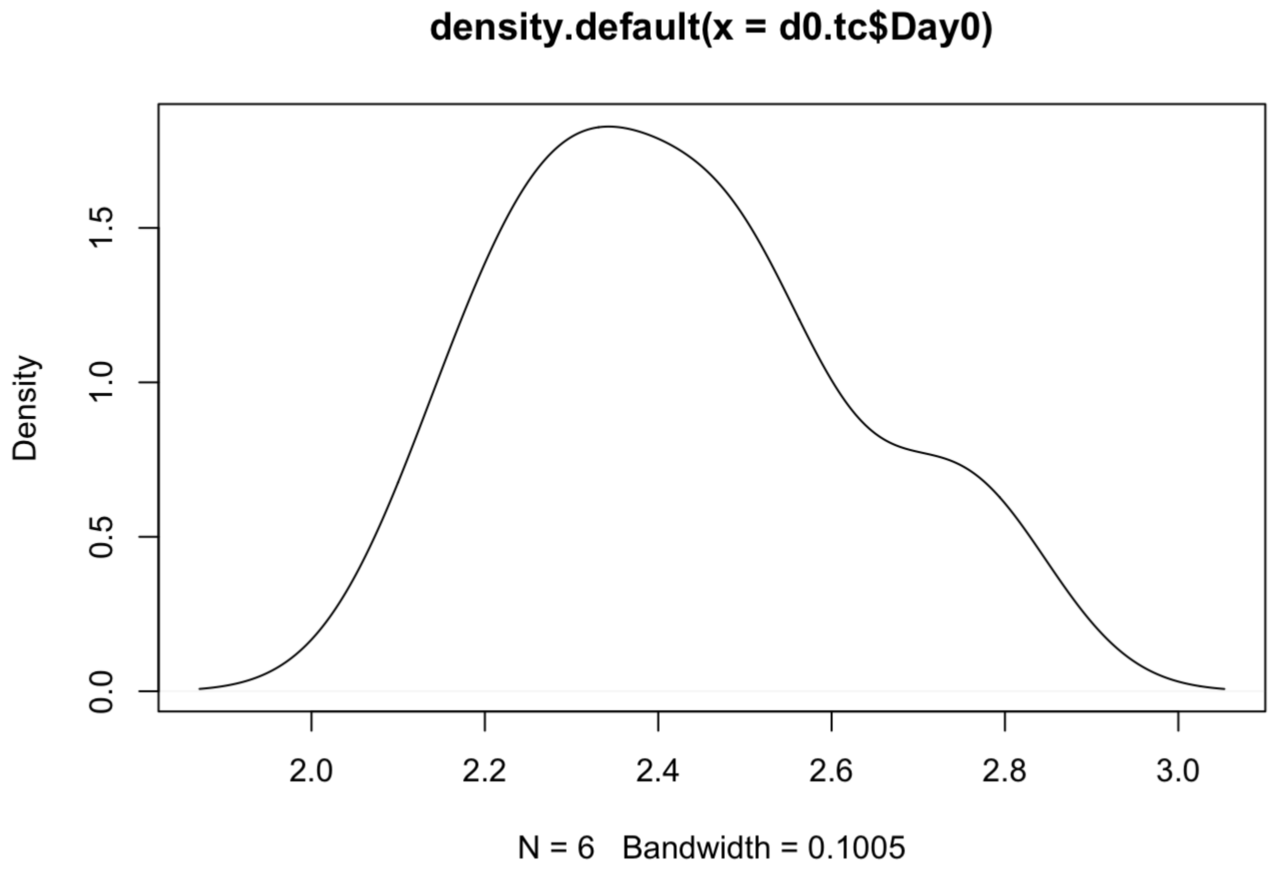


```
plot(density(d0.tc$lab_swab))
```

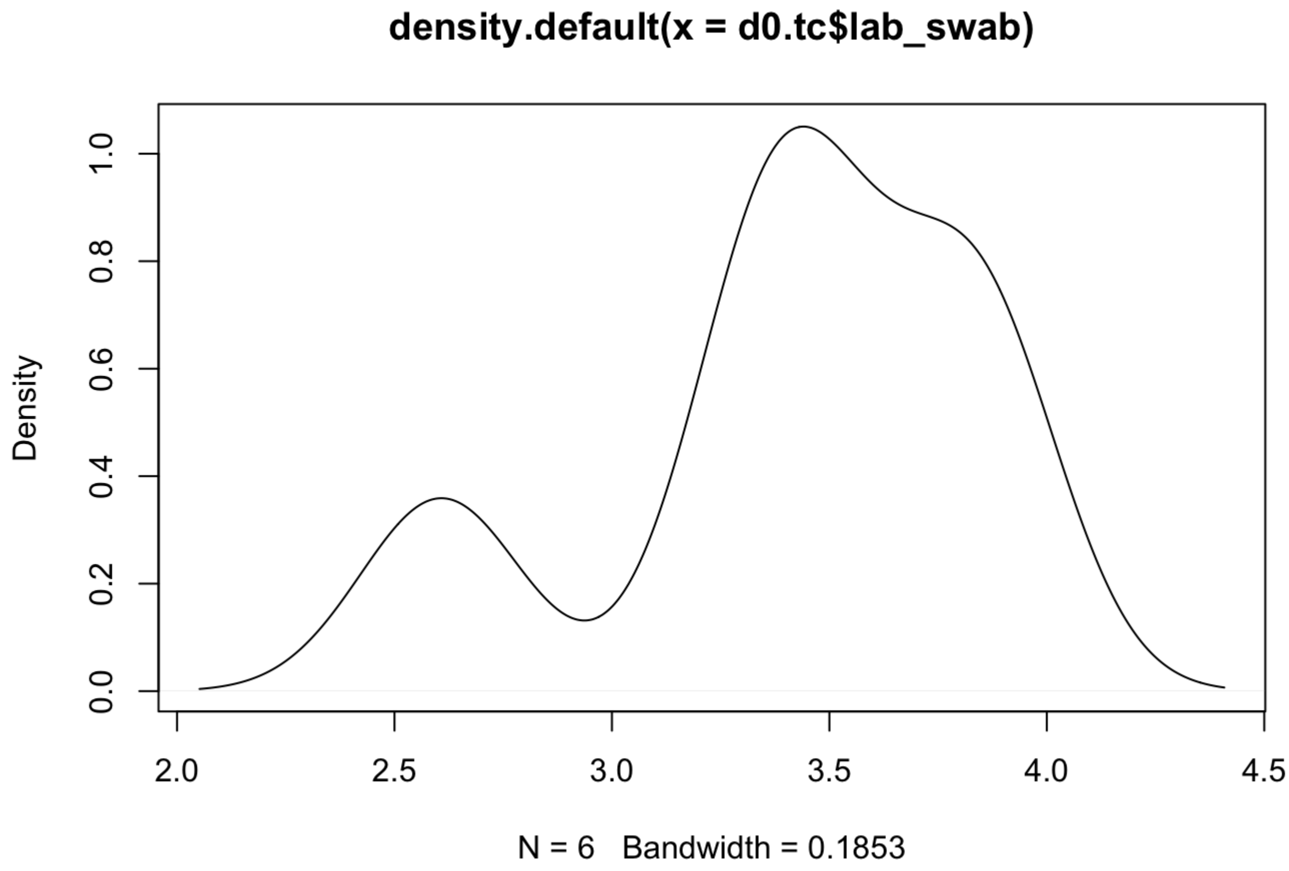

Correlation

```
d0.tc.cor <- cor.test(d0.tc$Day0, d0.tc$lab_swab, method = "pearson")
d0.tc.cor
```

```
##
##  Pearson's product-moment correlation
##
## data:  d0.tc$Day0 and d0.tc$lab_swab
## t = 2.2401, df = 4, p-value = 0.08861
## alternative hypothesis: true correlation is not equal to 0
## 95 percent confidence interval:
##  -0.1662469  0.9701810
## sample estimates:
##       cor
## 0.7459588
```

Richness

```
d0.tc.rich<- select(d0.ind.pair, toe.clip, type, Observed)

d0.tc.rich <- d0.tc.rich %>%
  spread(key = type, value = Observed)%>%
  group_by(toe.clip)
```

```
d0.tc.rich <- d0.tc.rich[d0.tc.rich$toe.clip != "9",]
```

Assumption check

```
plot(density(d0.tc.rich$Day0))
```

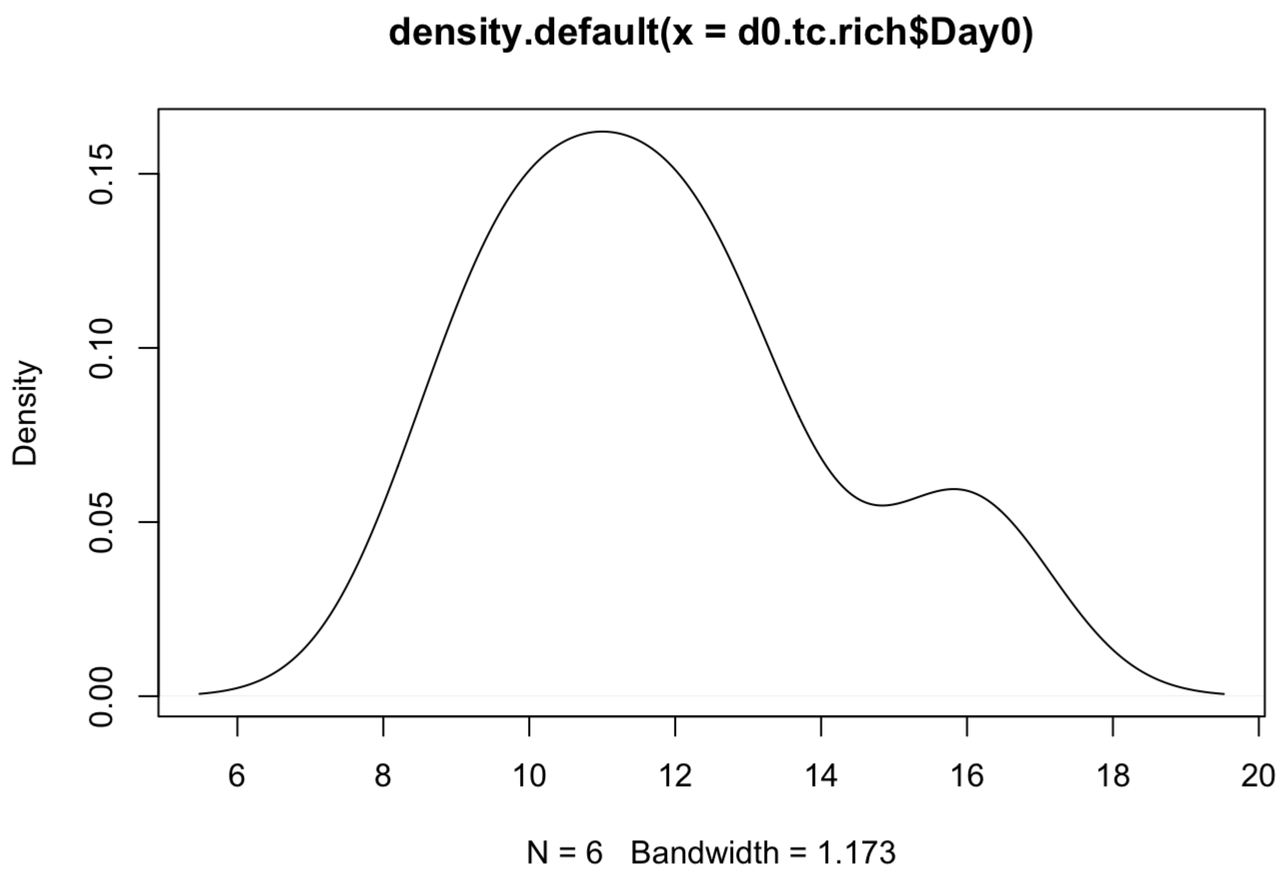


```
plot(density(d0.tc.rich$lab_swab))
```

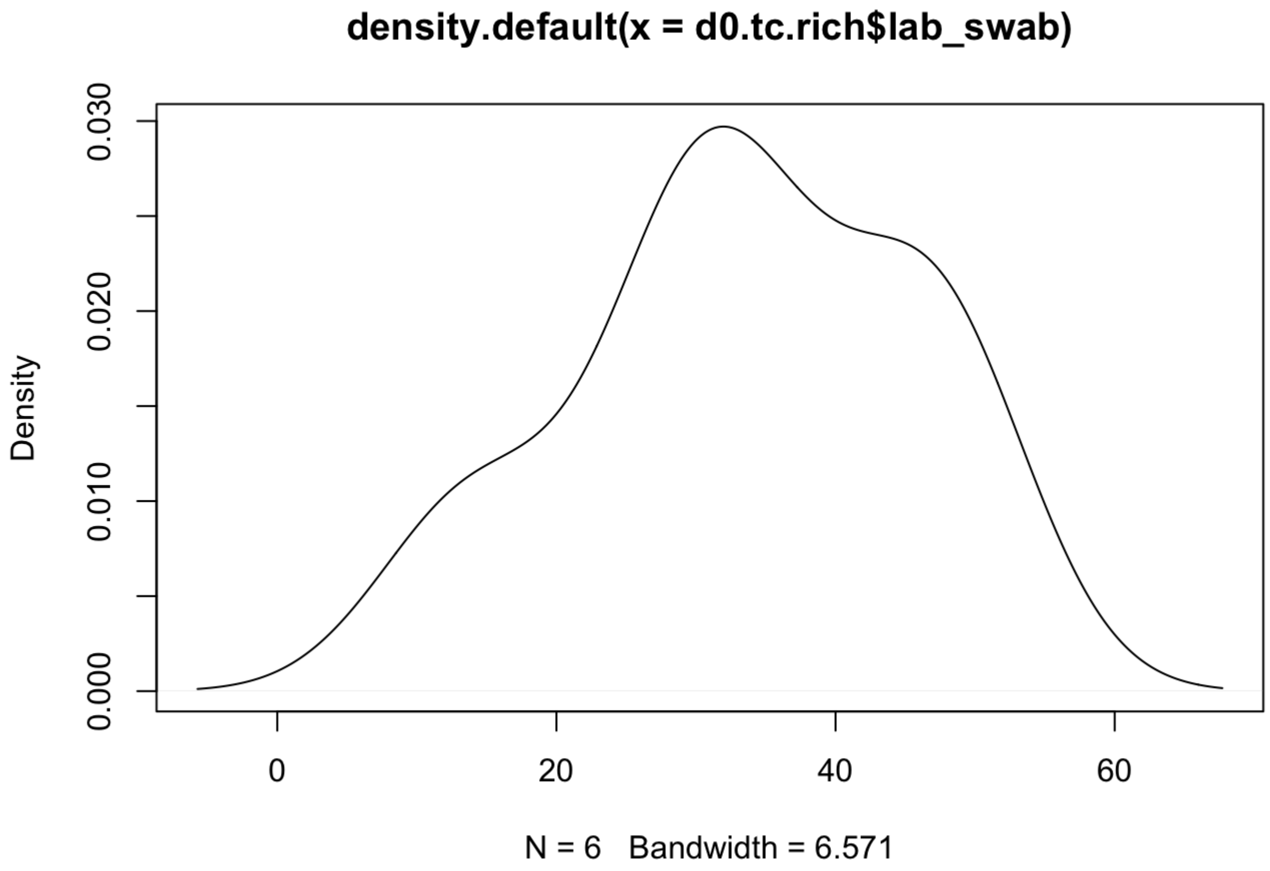

Correlation

```
d0.tc.rich.cor <- cor.test(d0.tc.rich$Day0, d0.tc.rich$lab_swab, method = "pearson")
d0.tc.rich.cor
```

```
##
##  Pearson's product-moment correlation
##
## data:  d0.tc.rich$Day0 and d0.tc.rich$lab_swab
## t = 2.1107, df = 4, p-value = 0.1024
## alternative hypothesis: true correlation is not equal to 0
## 95 percent confidence interval:
##  -0.2085116  0.9674947
## sample estimates:
##       cor
## 0.7258837
```

Dissected females

Data organization

Shannon Diversity

```
d0.diss.pair <- d0.pair[d0.pair$treatment == "Dissected",]

d0.tc.diss<- select(d0.diss.pair, toe.clip, type, Shannon)

d0.tc.diss <- d0.tc.diss %>%
  spread(key = type, value = Shannon)%>%
  group_by(toe.clip)
```

Assumption Check

```
plot(density(d0.tc.diss$Day0))
```

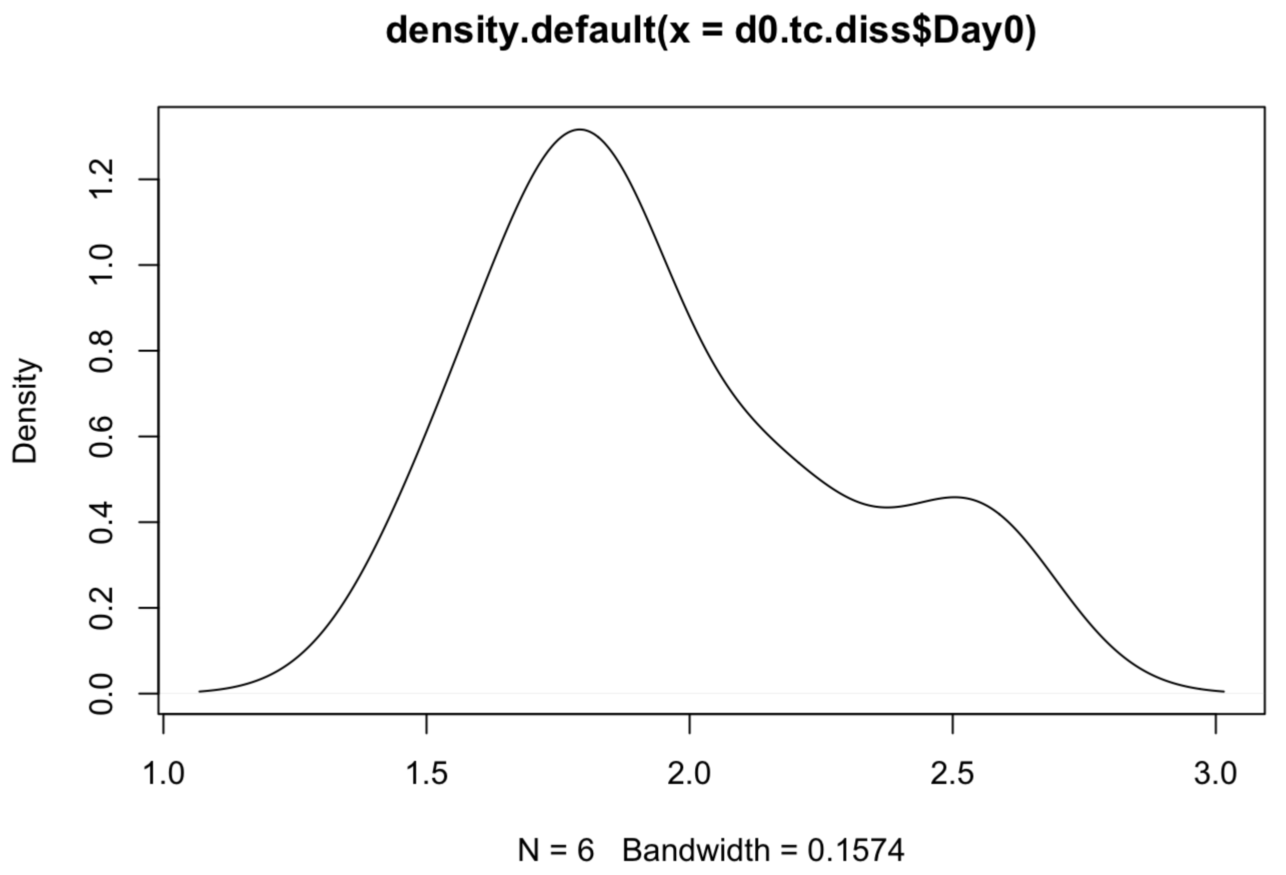


```
plot(density(d0.tc.diss$lab_swab))
```

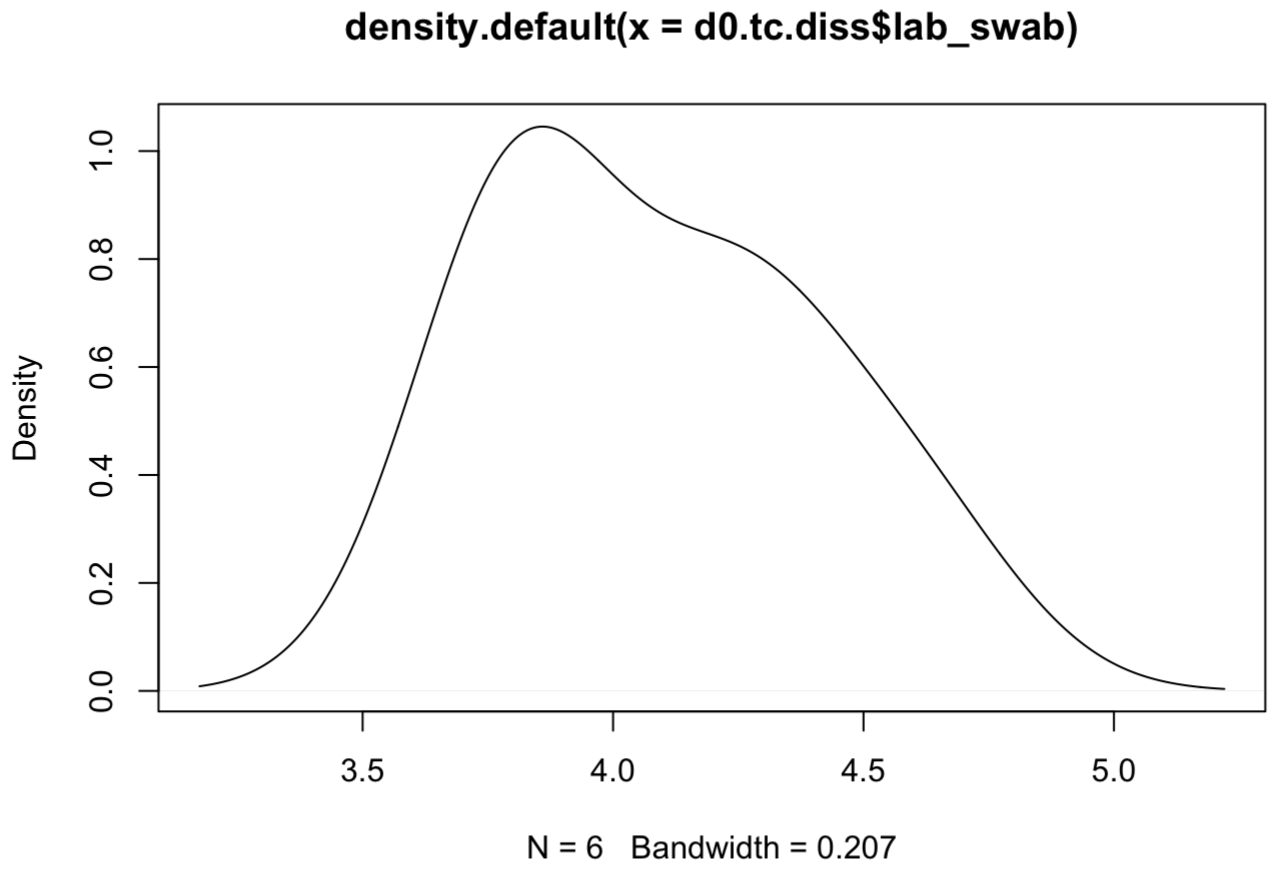

Correlation

```
d0.tc.diss.cor <- cor.test(d0.tc.diss$Day0, d0.tc.diss$lab_swab, method = "pearson")
d0.tc.diss.cor
```

```
##
##  Pearson's product-moment correlation
##
## data:  d0.tc.diss$Day0 and d0.tc.diss$lab_swab
## t = 0.16459, df = 4, p-value = 0.8773
## alternative hypothesis: true correlation is not equal to 0
## 95 percent confidence interval:
##  -0.7815672  0.8378117
## sample estimates:
##        cor
## 0.08201605
```

Richness

```
d0.tc.rich.diss<- select(d0.diss.pair, toe.clip, type, Observed)

d0.tc.rich.diss <- d0.tc.rich.diss %>%
  spread(key = type, value = Observed)%>%
  group_by(toe.clip)
```

Assumption Check

```
plot(density(d0.tc.rich.diss$Day0))
```

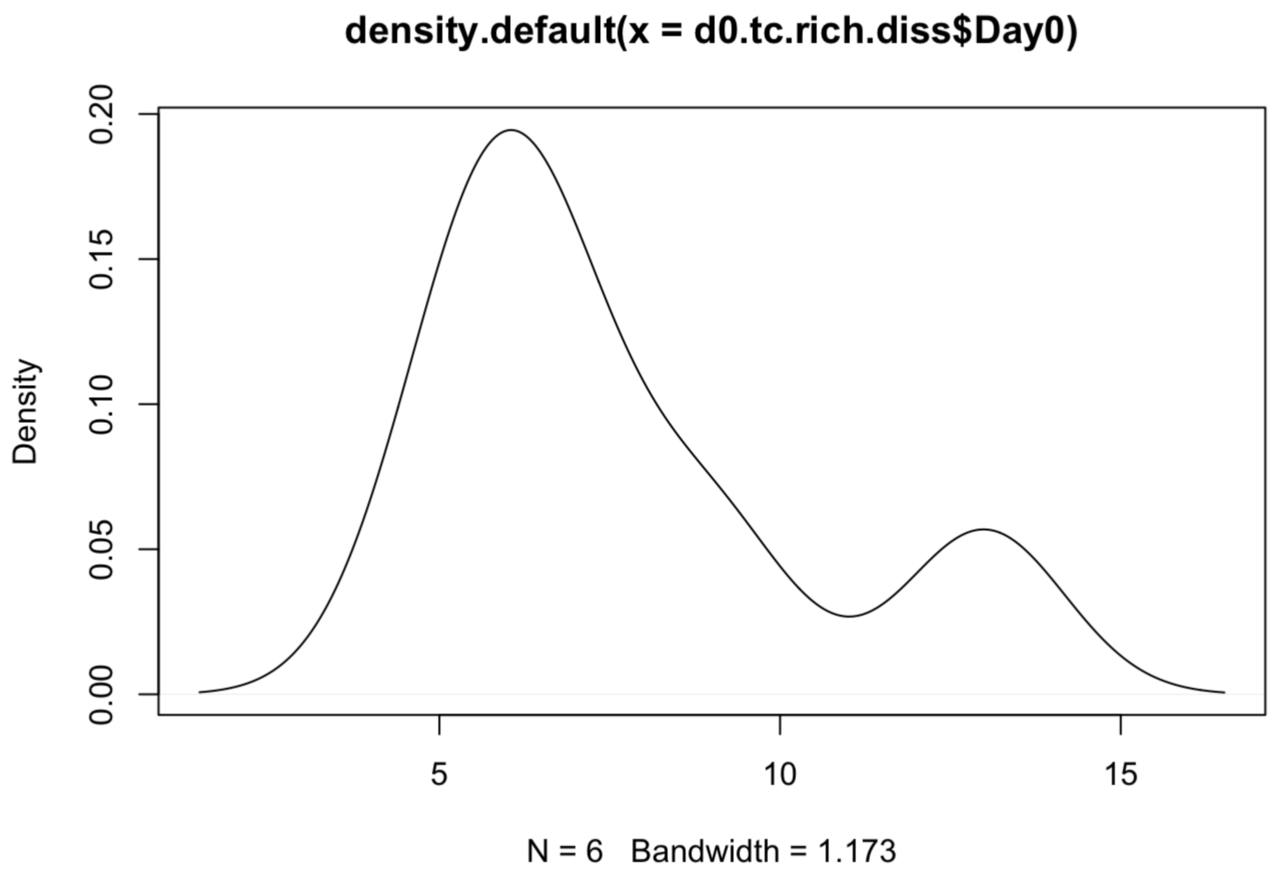


```
plot(density(d0.tc.rich.diss$lab_swab))
```

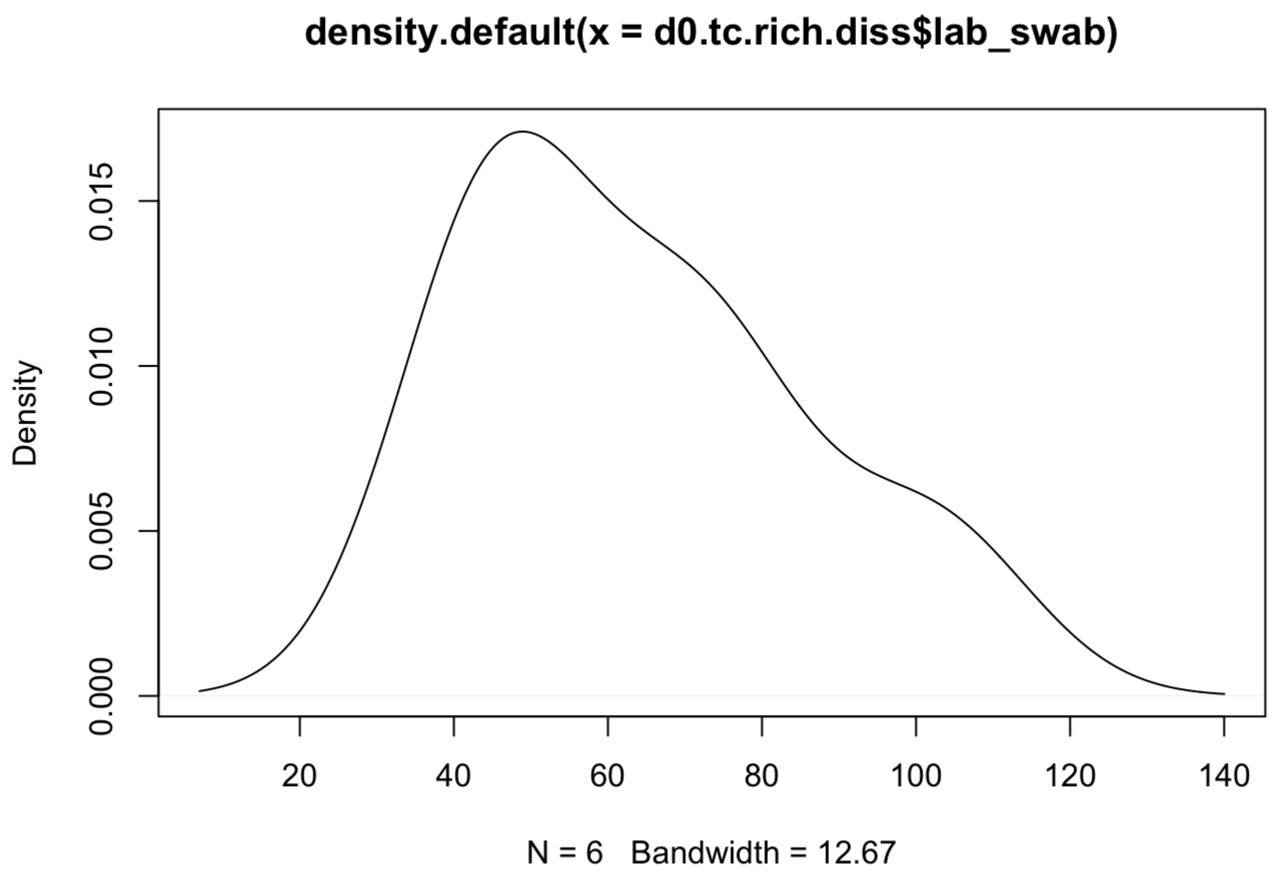

Correlation

```
d0.tc.rich.cor.diss <- cor.test(d0.tc.rich.diss$Day0, d0.tc.rich.diss$lab_swab, method = "pearso
n")
d0.tc.rich.cor.diss
```

```
##
##  Pearson's product-moment correlation
##
## data:  d0.tc.rich.diss$Day0 and d0.tc.rich.diss$lab_swab
## t = 0.080021, df = 4, p-value = 0.9401
## alternative hypothesis: true correlation is not equal to 0
## 95 percent confidence interval:
##  -0.7974562  0.8247799
## sample estimates:
##        cor
## 0.03997872
```

2017 HTS supplement

```
ee.meta <- read.csv("R_files/ee_meta_samples.csv", row.names = 1)
ee.counts <- read.csv("R_files/ee_counts_decontam.csv", row.names = 1)
ee.tax <- read.csv("R_files/ee_tax_decontam.csv", row.names = 1)

ee.counts <- as.data.frame(t(ee.counts))
```

```
str(ee.meta)
```

```
## 'data.frame':    22 obs. of  7 variables:
##  $ date        : chr  "9-Jul" "9-Jul" "9-Jul" "9-Jul" ...
##  $ form        : chr  "cloacal swab" "cloacal swab" "cloacal swab" "cloacal swab" ...
##  $ type        : chr  "d0" "d0" "d0" "d0" ...
##  $ toe.clip    : int  1144 1121 1135 1122 1312 1131 2323 1124 8504 1142 ...
##  $ treatment   : chr  "D" "A" "C" "D" ...
##  $ is.control  : logi  FALSE FALSE FALSE FALSE FALSE FALSE ...
##  $ illumina.run: chr  "dec" "march" "dec" "dec" ...
```

```
ee.meta$toe.clip <- as.factor(ee.meta$toe.clip)
ee.meta <- ee.meta[order(rownames(ee.meta)),]
ee.counts <- ee.counts[order(rownames(ee.counts)),]
```

```
ee.counts <- ee.counts[,colSums(ee.counts) > 10]
ee.tax <- ee.tax[rownames(ee.tax) %in% colnames(ee.counts),]

ee.counts.ps <- otu_table(ee.counts, taxa_are_rows = FALSE)
colnames(ee.counts.ps) <- colnames(ee.counts)

ee.tax.ps <- tax_table(ee.tax)
```

```
## Warning in .local(object): Coercing from data.frame class to character matrix
## prior to building taxonomyTable.
## This could introduce artifacts.
## Check your taxonomyTable, or coerce to matrix manually.
```

```
taxa_names(ee.tax.ps) <- taxa_names(ee.counts.ps)
colnames(ee.tax.ps) <- c("Kingdom","Phylum","Class","Order","Family","Genus")
ee.meta.ps <- sample_data(ee.meta)


ee.ps <- phyloseq(ee.counts.ps, ee.tax.ps, ee.meta.ps)

ee.fam.ps <- tax_glom(ee.ps, "Family", NArm = FALSE)
ee.ps <- tax_glom(ee.ps, "Family", NArm = FALSE)
```

```
richness <- estimate_richness(ee.ps, measures = "Observed")
```

```
## Warning in estimate_richness(ee.ps, measures = "Observed"): The data you have provided does n
ot have
## any singletons. This is highly suspicious. Results of richness
## estimates (for example) are probably unreliable, or wrong, if you have already
## trimmed low-abundance taxa from the data.
##
## We recommended that you find the un-trimmed data and retry.
```

```
ee.meta <- cbind(ee.meta, richness)
```

```
ee.ps@otu_table <- transform_sample_counts(ee.ps@otu_table,
                                                  function(x)  log10(x + 1))
```

```
shannon <- estimate_richness(ee.ps, measures = "Shannon")

ee.meta <- cbind(ee.meta, shannon)
ee.meta.ps <- sample_data(ee.meta)
ee.ps@sam_data <- ee.meta.ps
```

Alpha Diversity

Check Assumptions – Richness

```
ee.aov.rich <- aov(Observed ~ treatment, data = ee.meta)

plot(density(ee.aov.rich$residuals))
```

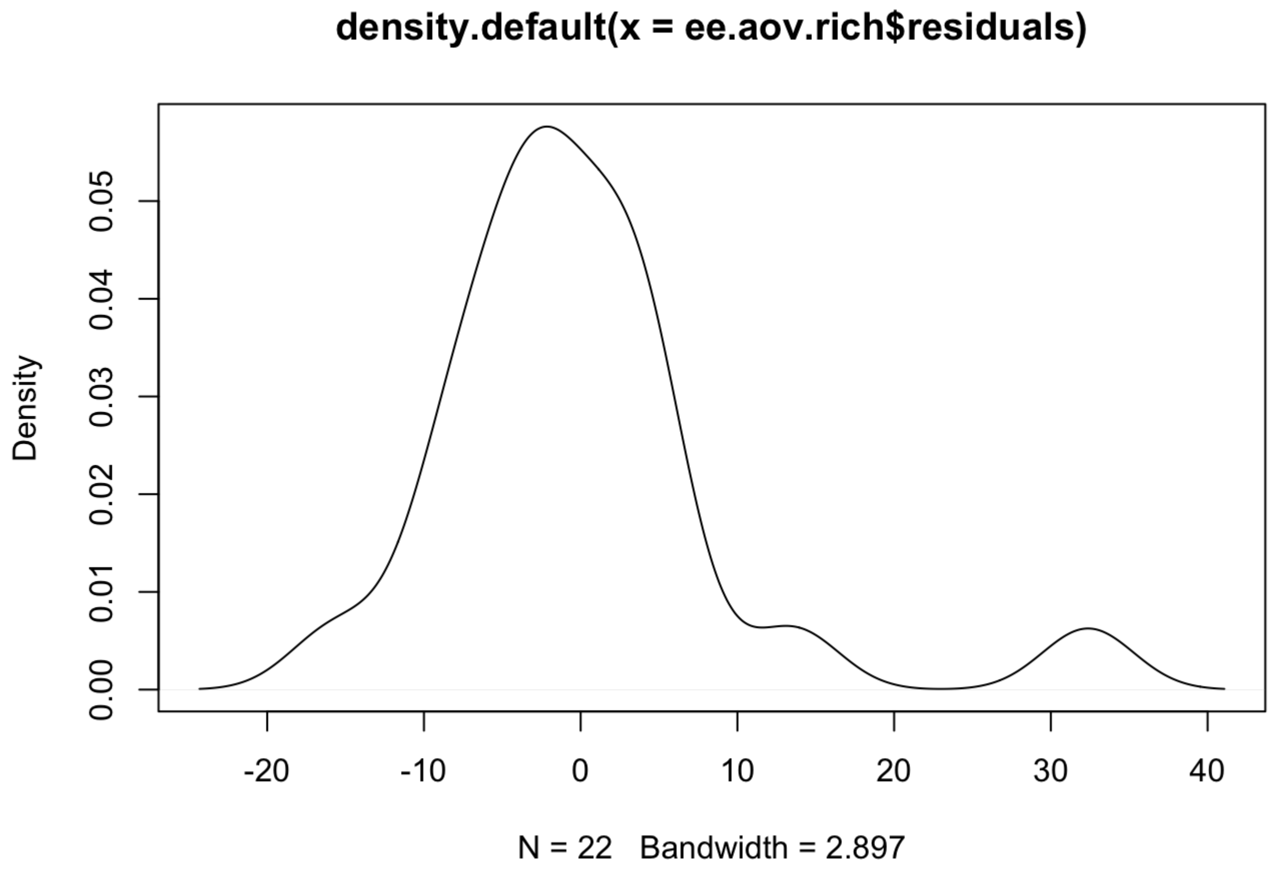


```
qqnorm(ee.aov.rich$residuals)
qqline(ee.aov.rich$residuals, datax = FALSE, distribution = qnorm, probs = c(0.25, 0.75))
```

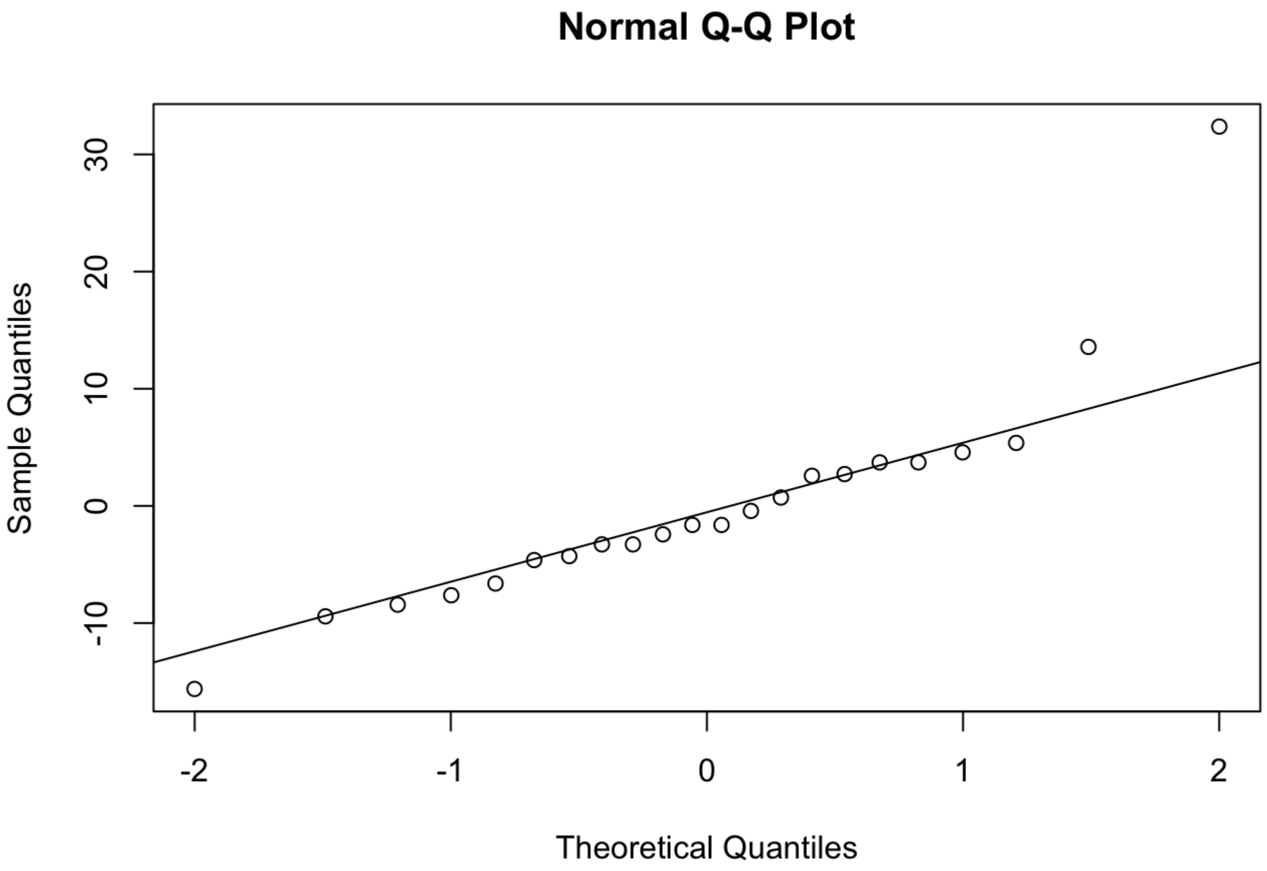


```
plot(ee.aov.rich$residuals~ee.aov.rich$fitted.values)
lines(lowess(ee.aov.rich$fitted.values,ee.aov.rich$residuals), col="blue")
```

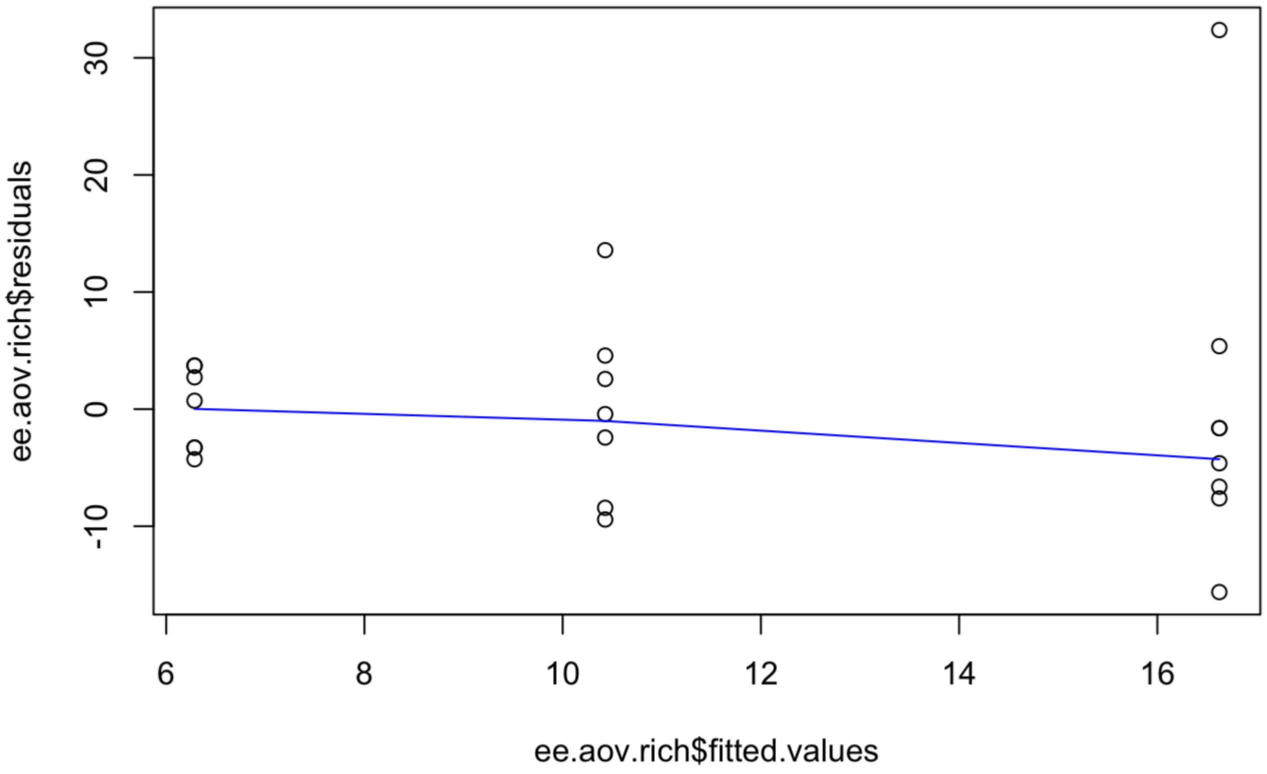

Model Results

```
summary(ee.aov.rich)
```

```
##             Df Sum Sq Mean Sq F value Pr(>F)
## treatment    2  408.1   204.0   2.037  0.158
## Residuals   19 1903.0   100.2
```

Check assumptions – Shannon

```
ee.aov <- aov(Shannon ~ treatment, data = ee.meta)

plot(density(ee.aov$residuals))
```

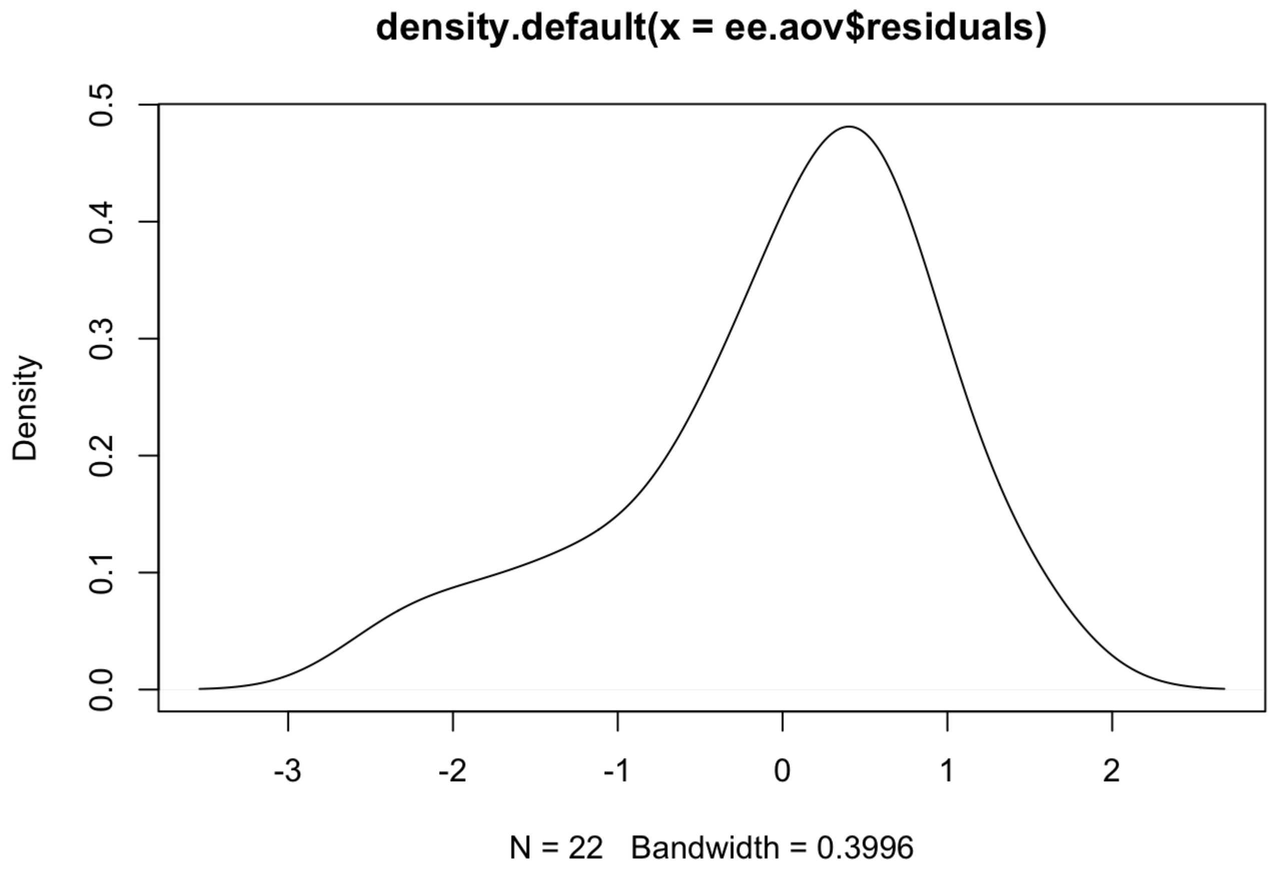


```
qqnorm(ee.aov$residuals)
qqline(ee.aov$residuals, datax = FALSE, distribution = qnorm, probs = c(0.25, 0.75))
```

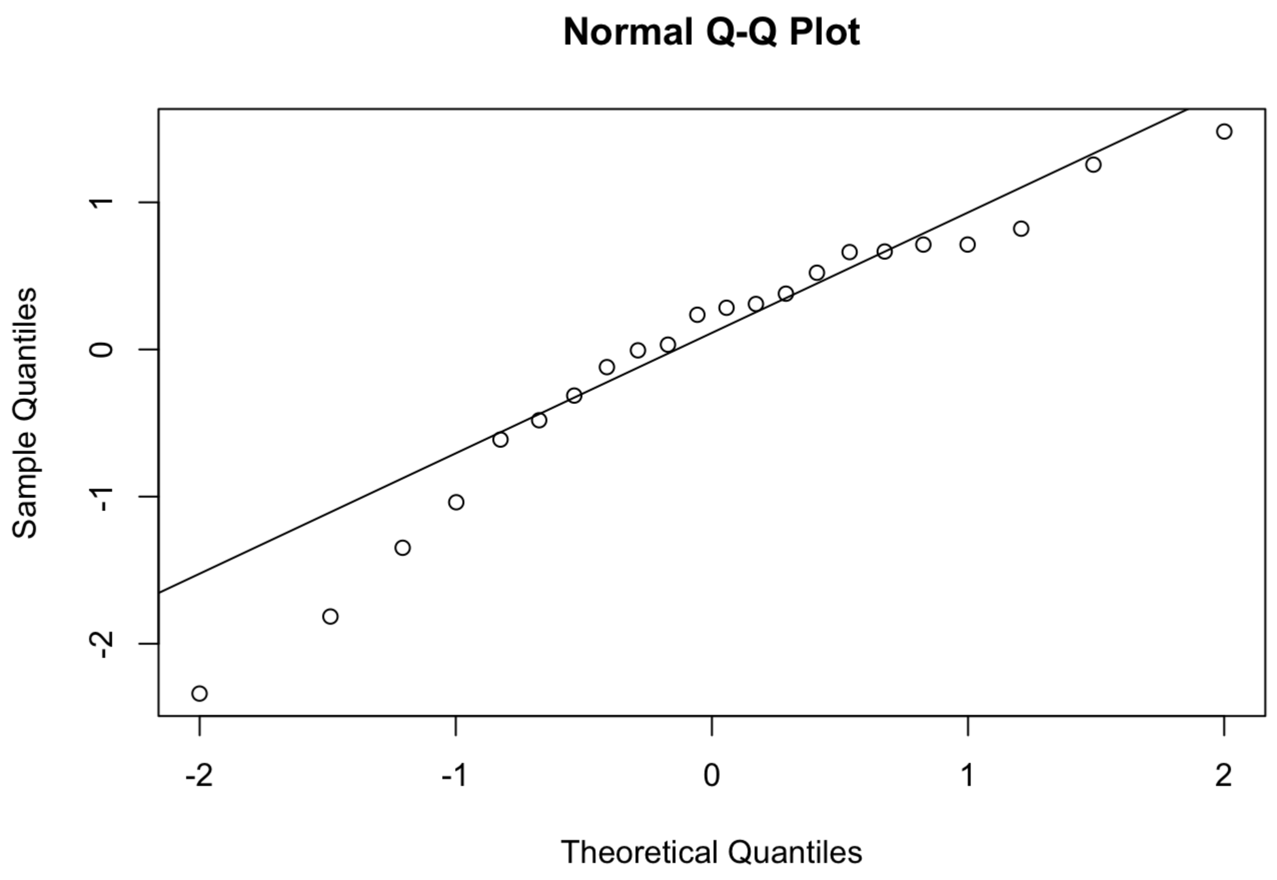


```
plot(ee.aov$residuals~ee.aov$fitted.values)
lines(lowess(ee.aov$fitted.values,ee.aov$residuals), col="blue")
```

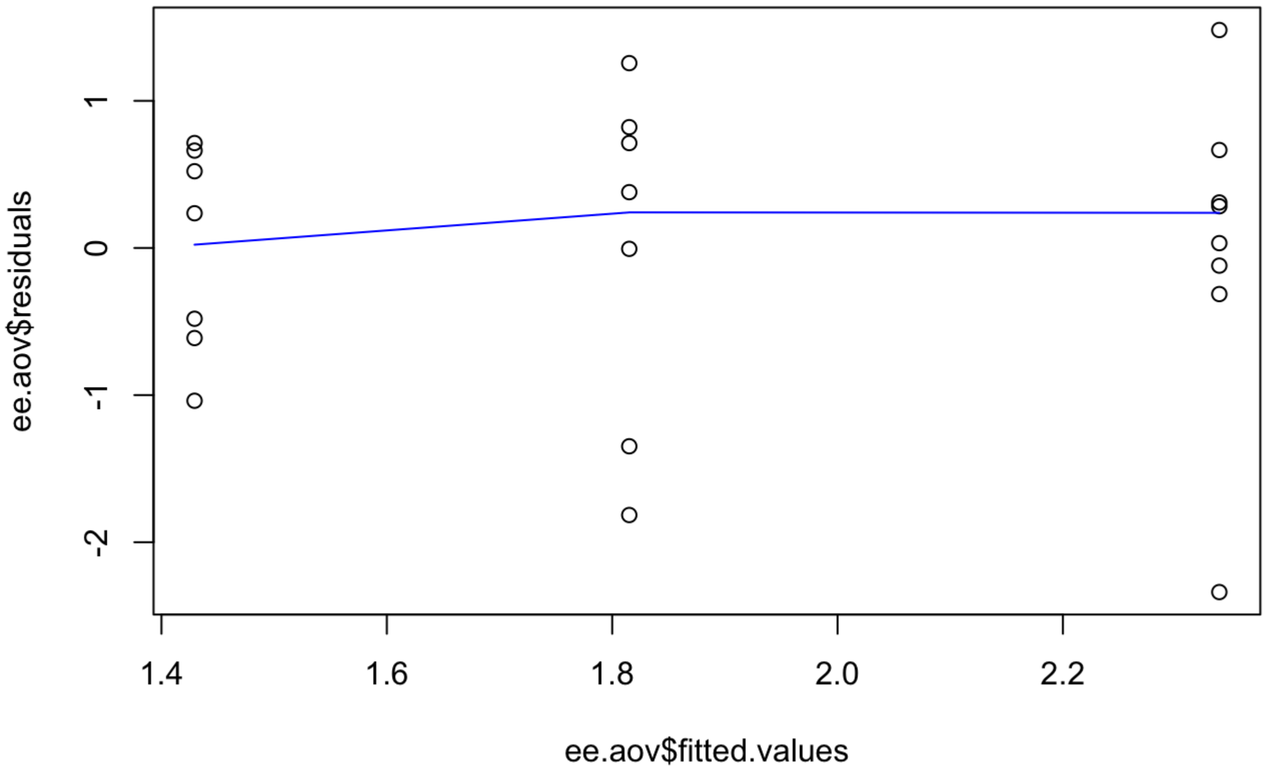


```
summary(ee.aov)
```

```
##             Df Sum Sq Mean Sq F value Pr(>F)
## treatment    2  3.135   1.567   1.537  0.241
## Residuals   19 19.371   1.020
```

Beta Diversity

```
ee.dist <- vegdist(ee.ps@otu_table, method = "bray")

anova(betadisper(ee.dist, ee.ps@sam_data[["treatment"]]))
```

```
## Analysis of Variance Table
##
## Response: Distances
##           Df  Sum Sq  Mean Sq F value Pr(>F)
## Groups     2 0.05248 0.026238  1.5116 0.2459
## Residuals 19 0.32980 0.017358
```

ok

```
set.seed(1)
ee.perma <- adonis(ee.dist ~ treatment, data = ee.meta)
ee.perma
```

```
##
## Call:
## adonis(formula = ee.dist ~ treatment, data = ee.meta)
##
## Permutation: free
## Number of permutations: 999
##
## Terms added sequentially (first to last)
##
##           Df SumsOfSqs MeanSqs F.Model      R2 Pr(>F)
## treatment  2    0.7789 0.38945  2.2827 0.19373  0.036 *
## Residuals 19    3.2416 0.17061         0.80627
## Total     21    4.0205                 1.00000
## ---
## Signif. codes:  0 '***' 0.001 '**' 0.01 '*' 0.05 '.' 0.1 ' ' 1
```
