# Supplementary material for: Vertically transmitted microbiome protects eggs from fungal infection and egg failure
Source: Anim Microbiome. 2021 Jun 16;3:43. doi: 10.1186/s42523-021-00104-5 (PMC8207602; doi:10.1186/s42523-021-00104-5)
Supplement: Supplementary file 5 — Additional file 5. R script for statistical analyses of all experimental data: SEM analyses, fungal attachment assays, hatch success, and hatchling phenotype. [file 42523_2021_104_MOESM5_ESM.pdf]

# Vertically transmitted microbiome protects eggs from fungal infection and egg failure: Experimental Data Analysis

M.E. Bunker, G. Elliott, H. Heyer-Gray, M. O. Martin, A. E. Arnold, and S. L. Weiss

12/16/2020

# Packages Used

```
library(tidyverse)
```

```
## ── Attaching packages ─────────────────────────────────────── tidyverse 1.3.0 ──
```

```
## ✓ ggplot2 3.3.2     ✓ purrr   0.3.4
## ✓ tibble  3.0.3     ✓ dplyr   1.0.2
## ✓ tidyr   1.1.2     ✓ stringr 1.4.0
## ✓ readr   1.3.1     ✓ forcats 0.5.0
```

```
## ── Conflicts ────────────────────────────────────────── tidyverse_conflicts() ──
## x dplyr::filter() masks stats::filter()
## x dplyr::lag()    masks stats::lag()
```

```
library(dplyr)
library(ggplot2)
library(vegan)
```

```
## Loading required package: permute
```

```
## Loading required package: lattice
```

```
## This is vegan 2.5-6
```

```
library(gridExtra)
```

```
##
## Attaching package: 'gridExtra'
```

```
## The following object is masked from 'package:dplyr':
##
##     combine
```

```r
library(outliers)
```

```
##
## Attaching package: 'outliers'
```

```
## The following object is masked from 'package:vegan':
##
##     scores
```

```r
library(lmerTest)
```

```
## Loading required package: lme4
```

```
## Loading required package: Matrix
```

```
##
## Attaching package: 'Matrix'
```

```
## The following objects are masked from 'package:tidyr':
##
##     expand, pack, unpack
```

```
##
## Attaching package: 'lmerTest'
```

```
## The following object is masked from 'package:lme4':
##
##     lmer
```

```
## The following object is masked from 'package:stats':
##
##     step
```

```r
library(lme4)
library(readr)
```

# Data

SEM data and fungal attachment assay

```r
sem.b <- read.csv("R_files/sem_all.csv")

fa <- read.csv("R_files/HMFE.csv")
```

Hatch success and hatchling demographics

```
gh <- read.csv("R_files/gh_hatchsuccess.csv")

gh.long <- read.csv("R_files/gh_by_egg_meta.csv")
gh.long$MomTC<-factor(gh.long$MomTC)

babes <-gh.long[complete.cases(gh.long),]

babes<- babes[babes$MomTC != "3",]
babes<- babes[babes$MomTC != "21",]
```

## Notes on animals removed from “babes” dataframe

“babes” dataframe only has data for eggs that hatched. Mothers that only had eggs survive in the sterile environment were removed as the effect of incubation environment could not be compared for those animals.

# SEM Imaging Results

## Bacteria

Split data by day because we expected bacterial communities to grow during incubation and were uninterested in that effect. As well, the methods to quantify bacteria were different on day 25 than day 0.

```
sem.b.d0 <- sem.b[sem.b$Day == "Day0",]
sem.b.d25 <- sem.b[sem.b$Day == "Day25",]
```

Assumptions Check

```
mean.b.d0 <- lm(AvgB ~ MomTrmt, data = sem.b.d0)

densityplot(~ residuals(mean.b.d0), group = MomTrmt, data = sem.b.d0, auto.key=TRUE)
```

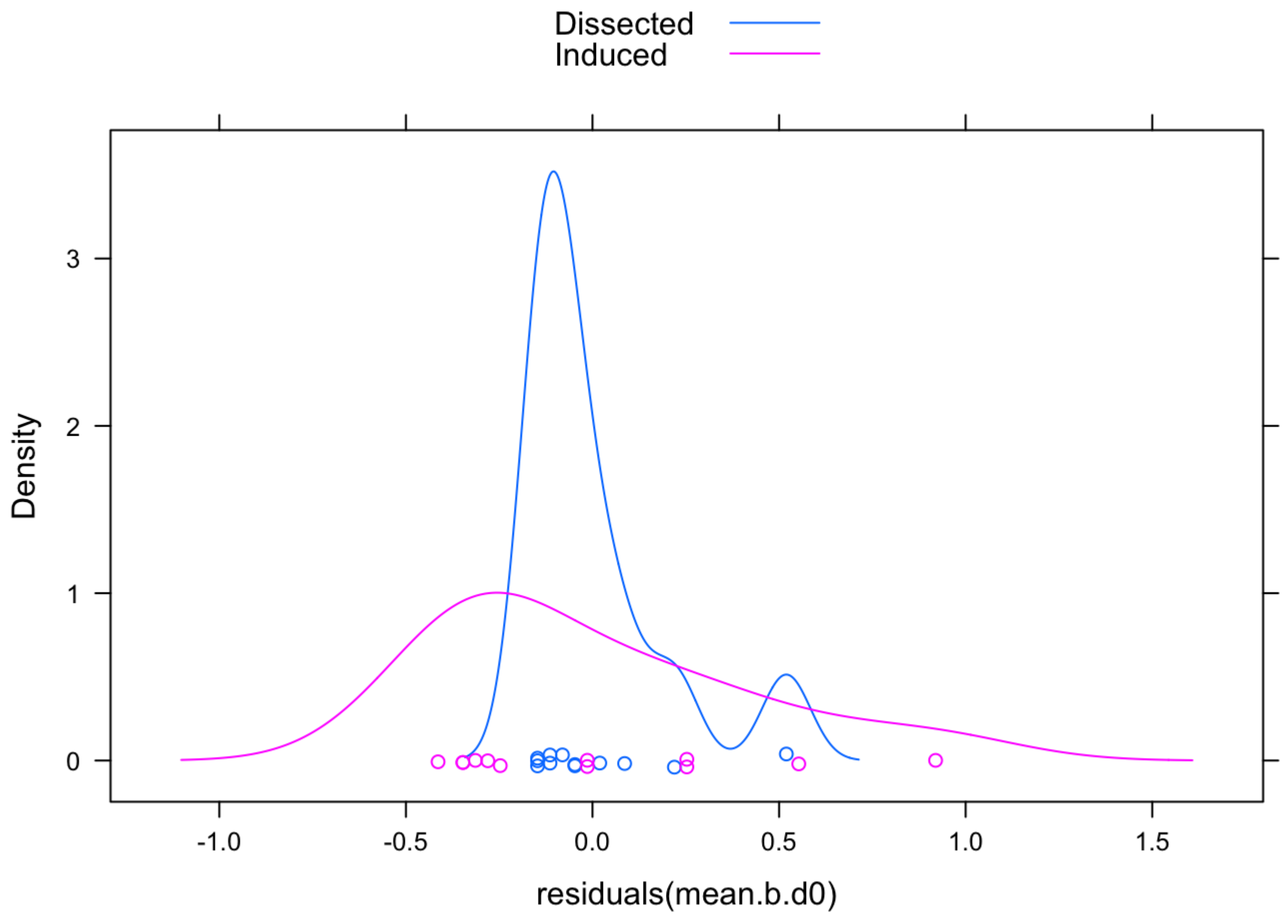


```
with(sem.b.d0, shapiro.test(AvgB[MomTrmt == "Dissected"]))
```

```
##
##  Shapiro-Wilk normality test
##
## data:  AvgB[MomTrmt == "Dissected"]
## W = 0.75962, p-value = 0.003374
```

```
with(sem.b.d0, shapiro.test(AvgB[MomTrmt == "Induced"]))
```

```
##
##  Shapiro-Wilk normality test
##
## data:  AvgB[MomTrmt == "Induced"]
## W = 0.86455, p-value = 0.05576
```

```
sem.b.d0$logB <- log10(sem.b.d0$AvgB)
mean.b.d0.log <- lm(logB ~ MomTrmt, data = sem.b.d0)

densityplot(~ residuals(mean.b.d0.log), group = MomTrmt, data = sem.b.d0, auto.key=TRUE)
```

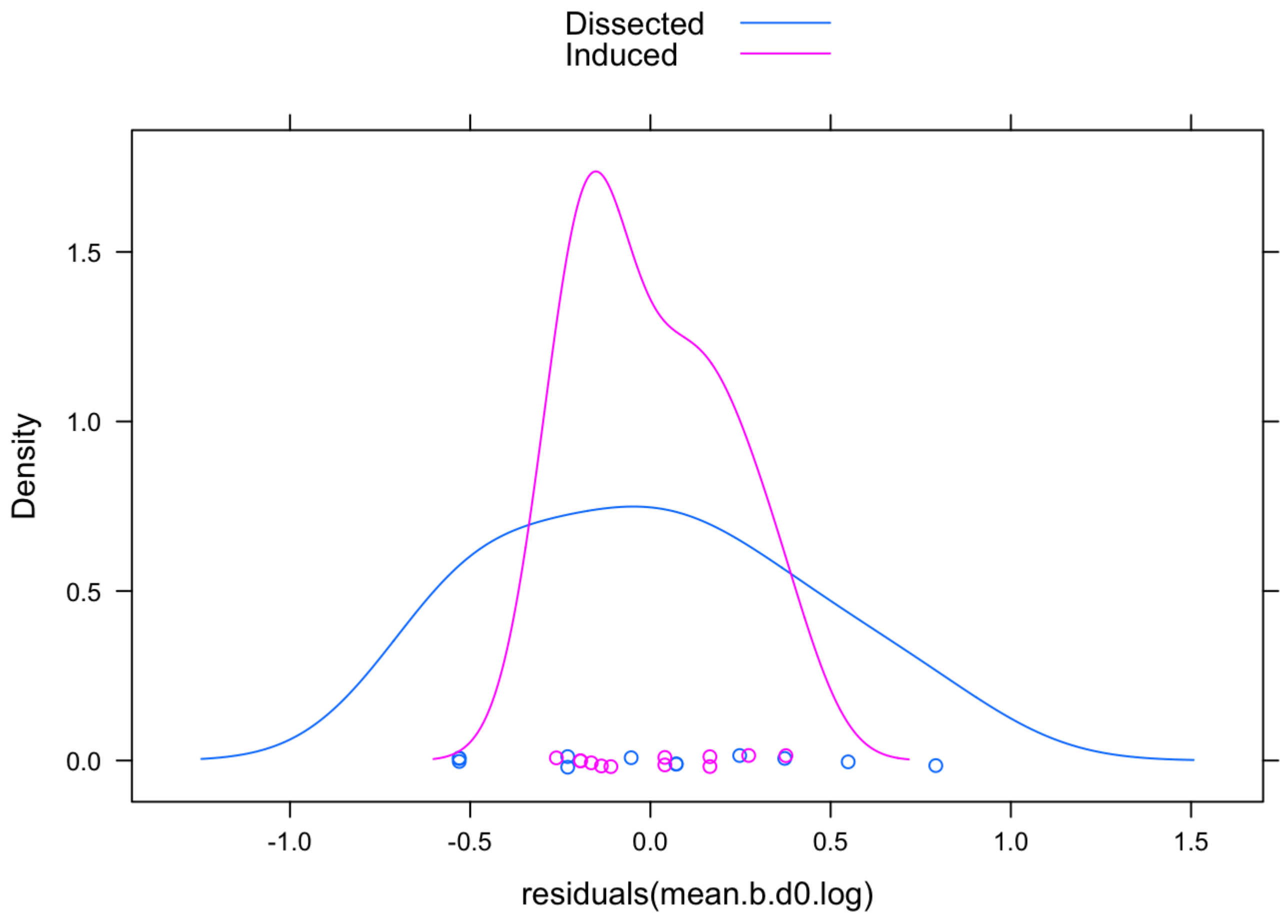


```
with(sem.b.d0, shapiro.test(logB[MomTrmt == "Dissected"]))
```

```
##
##  Shapiro-Wilk normality test
##
## data:  logB[MomTrmt == "Dissected"]
## W = 0.9401, p-value = 0.4994
```

```
with(sem.b.d0, shapiro.test(logB[MomTrmt == "Induced"]))
```

```
##
##  Shapiro-Wilk normality test
##
## data:  logB[MomTrmt == "Induced"]
## W = 0.9219, p-value = 0.3021
```

```
d0.b.t <- t.test(logB ~ MomTrmt, data = sem.b.d0, var.equal = TRUE)
d0.b.t
```

```
##
##  Two Sample t-test
##
## data:  logB by MomTrmt
## t = -5.8083, df = 22, p-value = 7.635e-06
## alternative hypothesis: true difference in means is not equal to 0
## 95 percent confidence interval:
##  -1.0987225 -0.5205548
## sample estimates:
## mean in group Dissected   mean in group Induced
##              -0.9466260              -0.1369873
```

```
mean.b.d25 <- lm(AvgB ~ MomTrmt, data = sem.b.d25)

densityplot(~ residuals(mean.b.d25), group = MomTrmt, data = sem.b.d25, auto.key=TRUE)
```

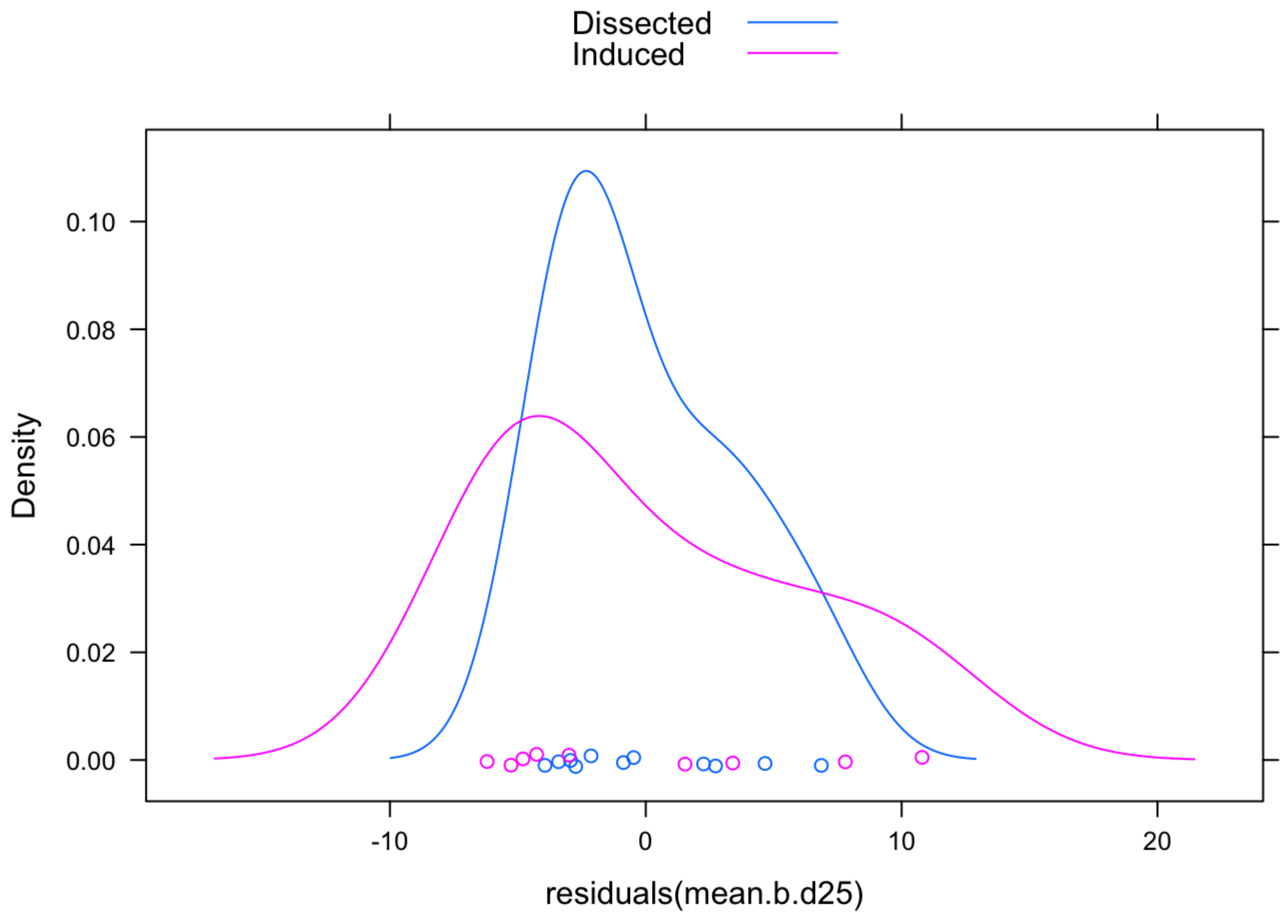


```
with(sem.b.d25, shapiro.test(AvgB[MomTrmt == "Dissected"]))
```

```
##
##  Shapiro-Wilk normality test
##
## data:  AvgB[MomTrmt == "Dissected"]
## W = 0.90205, p-value = 0.1958
```

```
with(sem.b.d25, shapiro.test(AvgB[MomTrmt == "Induced"]))
```

```
##
##  Shapiro-Wilk normality test
##
## data:  AvgB[MomTrmt == "Induced"]
## W = 0.87603, p-value = 0.1426
```

```
d25.b.test <- t.test(AvgB ~ MomTrmt, data = sem.b.d25, var.equal = TRUE)
d25.b.test
```

```
##
##  Two Sample t-test
##
## data:  AvgB by MomTrmt
## t = -6.823, df = 18, p-value = 2.185e-06
## alternative hypothesis: true difference in means is not equal to 0
## 95 percent confidence interval:
##  -19.78525 -10.46930
## sample estimates:
## mean in group Dissected   mean in group Induced
##                4.806061               19.933333
```

# Fungus

```
f.d25 <- lm(fungus ~ MomTrmt, data = sem.b.d25)

densityplot(~ residuals(f.d25), group = MomTrmt, data = sem.b.d25, auto.key=TRUE)
```

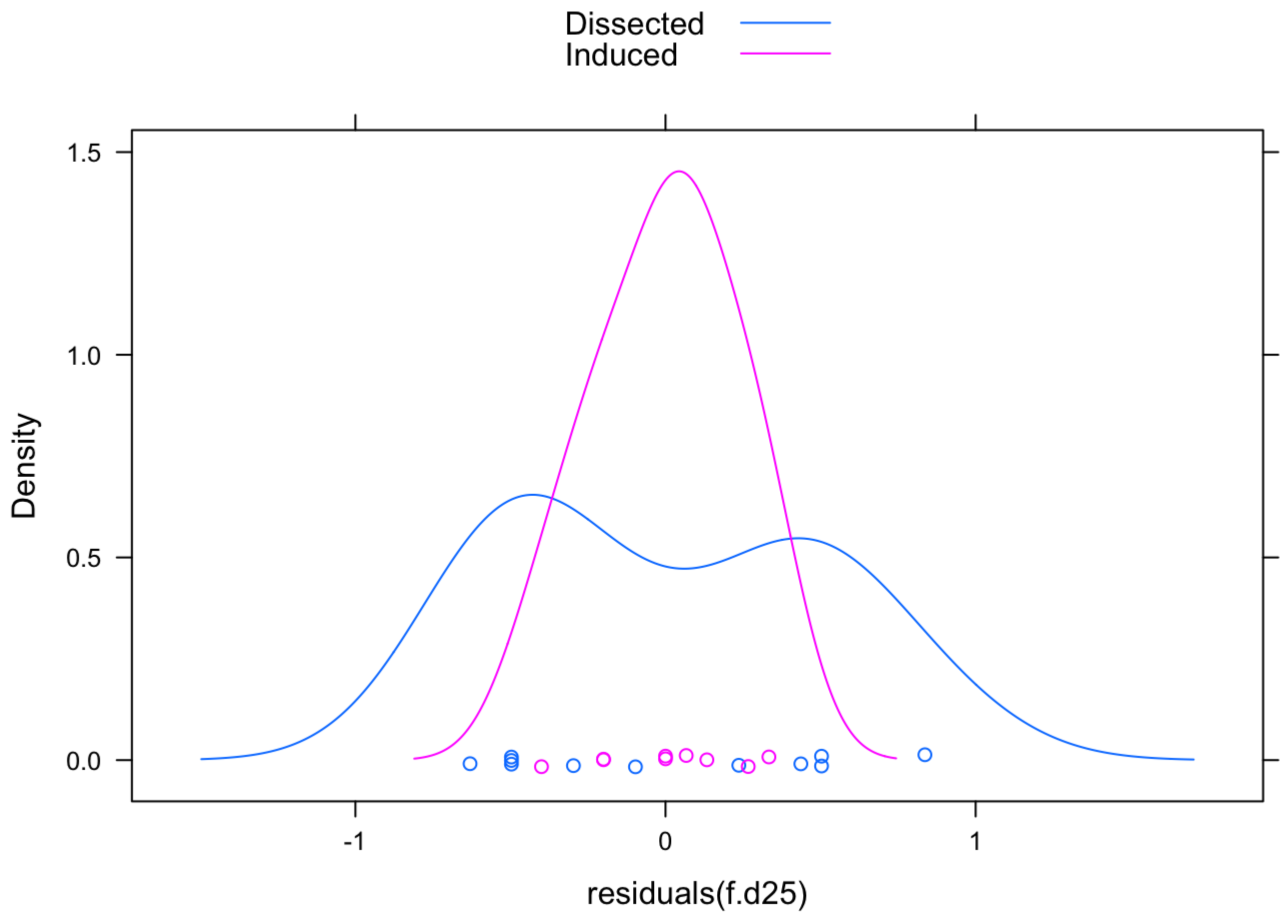


```
with(sem.b.d25, shapiro.test(fungus[MomTrmt == "Dissected"]))
```

```
##
##  Shapiro-Wilk normality test
##
## data:  fungus[MomTrmt == "Dissected"]
## W = 0.89369, p-value = 0.1546
```

```
with(sem.b.d25, shapiro.test(fungus[MomTrmt == "Induced"]))
```

```
##
##  Shapiro-Wilk normality test
##
## data:  fungus[MomTrmt == "Induced"]
## W = 0.96653, p-value = 0.8635
```

```
fungal.t <- t.test(fungus ~ MomTrmt, data =  sem.b.d25, var.equal = TRUE)
fungal.t
```

```
##
##  Two Sample t-test
##
## data:  fungus by MomTrmt
## t = 3.0062, df = 18, p-value = 0.007582
## alternative hypothesis: true difference in means is not equal to 0
## 95 percent confidence interval:
##  0.1697324 0.9575403
## sample estimates:
## mean in group Dissected   mean in group Induced
##               0.9636364               0.4000000
```

# Fungal Attachment Assay

We are not comparing attachment rates between fungal types because they were administered in different concentrations. Thus, separately for each fungal species, we analyze the effect of egg type on attachment of fungi.

In data file HM is Neocosmospora, FE is Aspergillus

## Aspergillus

```
fa.FE <- fa[fa$Fungus == "FE",]

fa.fe.lm <- lm(AvFungus ~ MomTrmt, data = fa.FE)

densityplot(~ residuals(fa.fe.lm), group = MomTrmt, data = fa.FE, auto.key=TRUE)
```

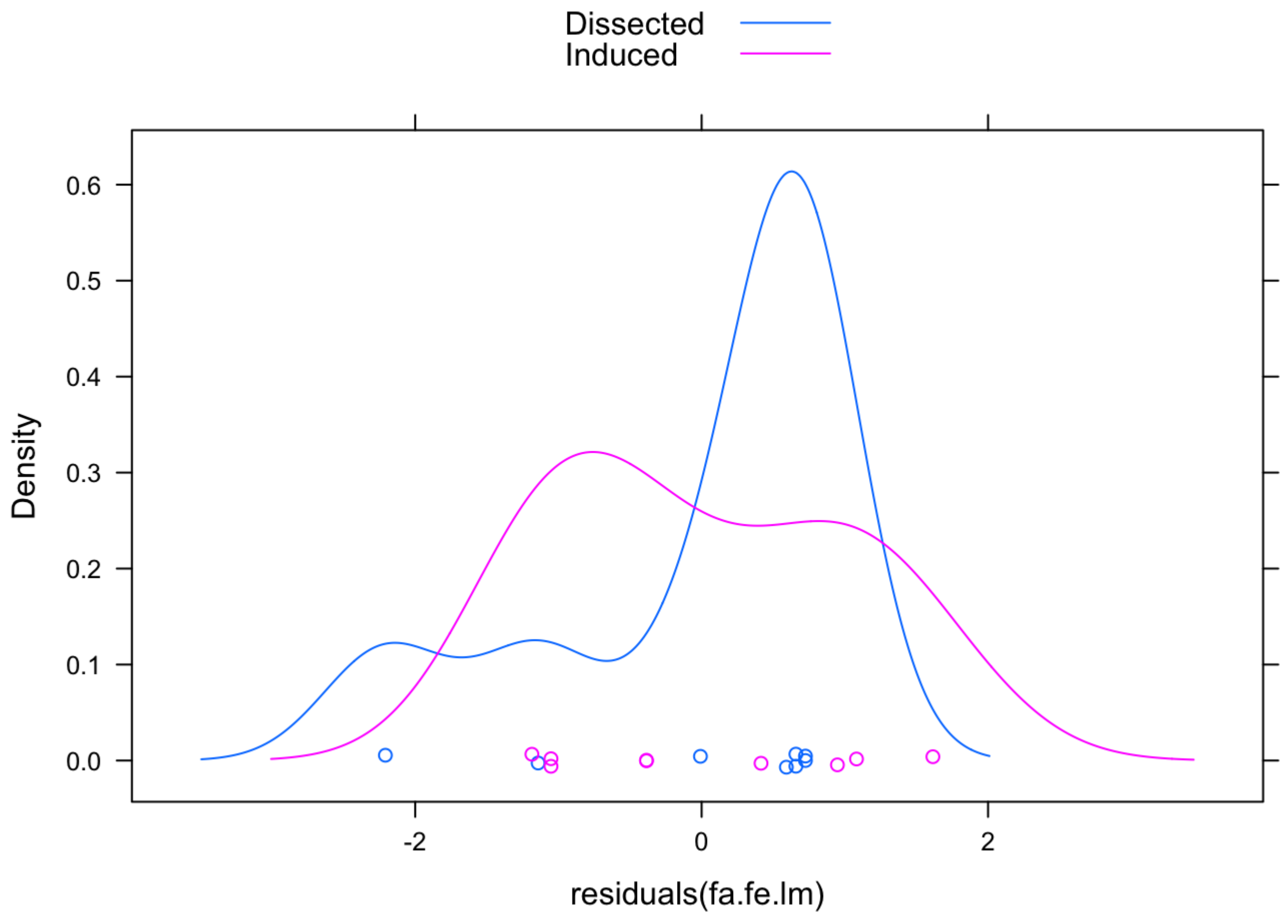


```
with(fa.FE, shapiro.test(AvFungus[MomTrmt == "Dissected"]))
```

```
##
##  Shapiro-Wilk normality test
##
## data:  AvFungus[MomTrmt == "Dissected"]
## W = 0.7314, p-value = 0.005076
```

```
with(fa.FE, shapiro.test(AvFungus[MomTrmt == "Induced"]))
```

```
##
##  Shapiro-Wilk normality test
##
## data:  AvFungus[MomTrmt == "Induced"]
## W = 0.90271, p-value = 0.2682
```

Log transformed

```
fa.fe.lm.log <- lm(LogFungus ~ MomTrmt, data = fa.FE)

densityplot(~ residuals(fa.fe.lm.log), group = MomTrmt, data = fa.FE, auto.key=TRUE)
```

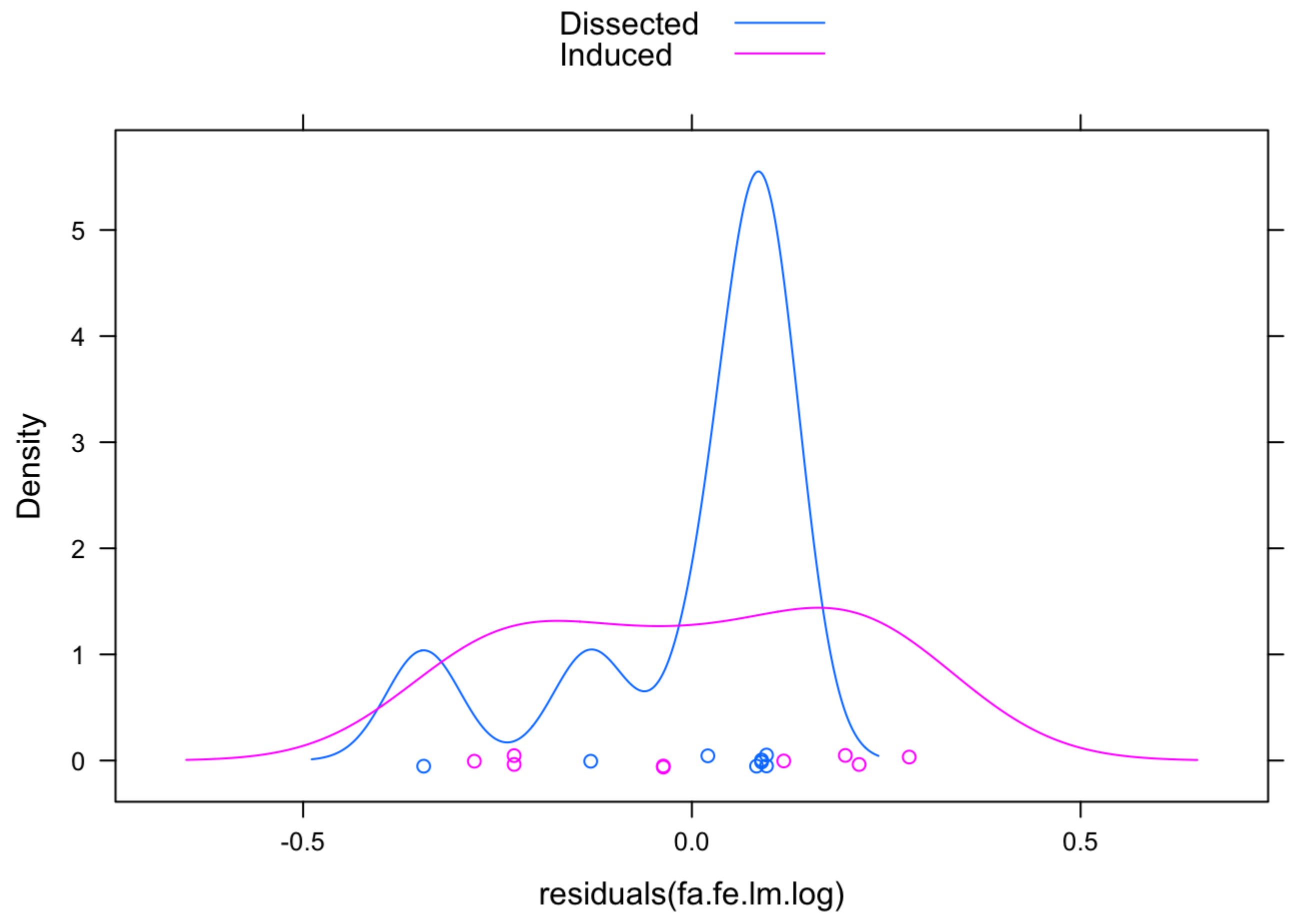


```
with(fa.FE, shapiro.test(LogFungus[MomTrmt == "Dissected"]))
```

```
##
##  Shapiro-Wilk normality test
##
## data:  LogFungus[MomTrmt == "Dissected"]
## W = 0.69214, p-value = 0.001835
```

```
with(fa.FE, shapiro.test(LogFungus[MomTrmt == "Induced"]))
```

```
##
##  Shapiro-Wilk normality test
##
## data:  LogFungus[MomTrmt == "Induced"]
## W = 0.90265, p-value = 0.2678
```

Square root transform

```
fa.FE$sqrtF <- sqrt(fa.FE$AvFungus)

fa.fe.lm.sq <- lm(sqrtF ~ MomTrmt, data = fa.FE)

densityplot(~ residuals(fa.fe.lm.sq), group = MomTrmt, data = fa.FE, auto.key=TRUE)
```

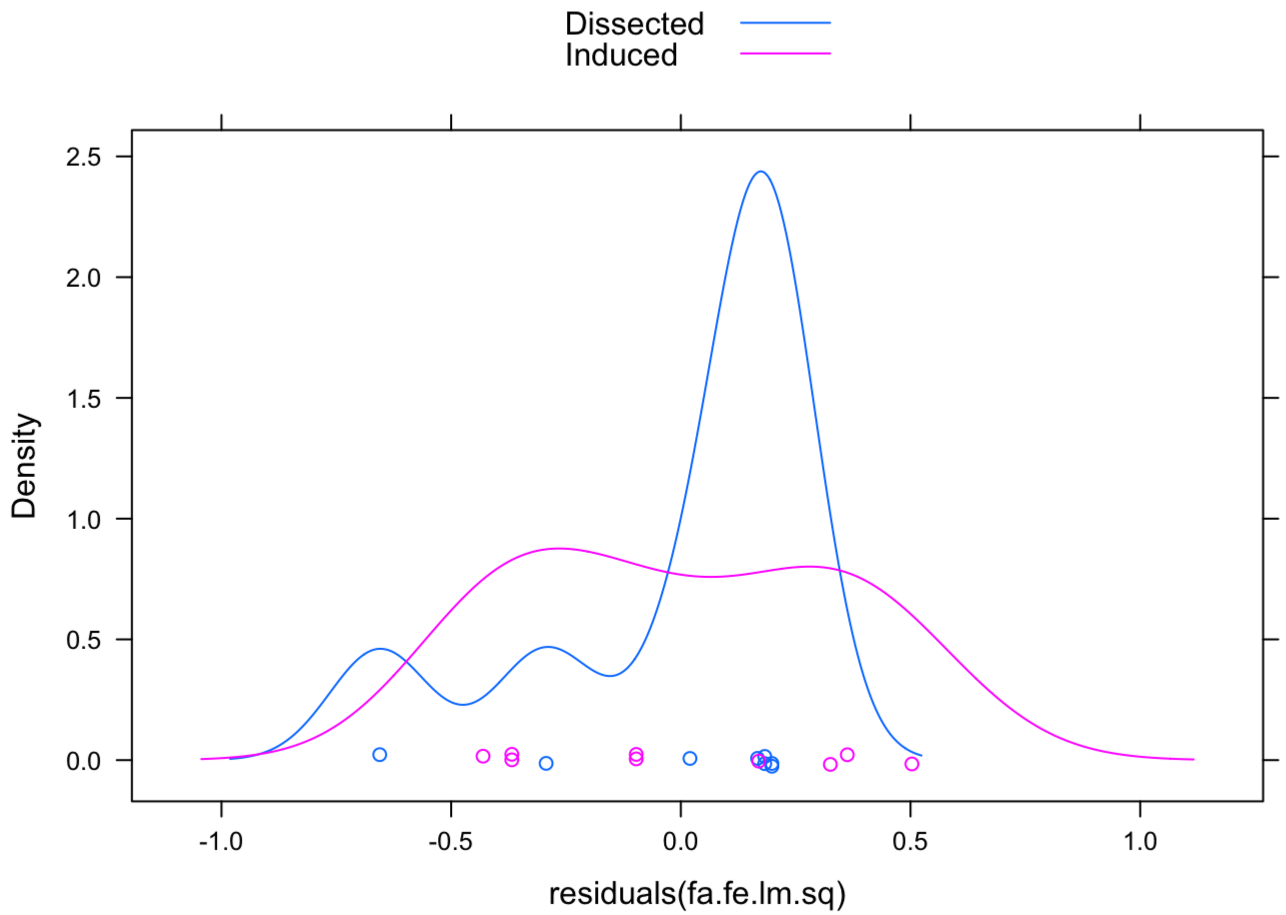


```
with(fa.FE, shapiro.test(sqrtF[MomTrmt == "Dissected"]))
```

```
##
##  Shapiro-Wilk normality test
##
## data:  sqrtF[MomTrmt == "Dissected"]
## W = 0.71379, p-value = 0.003223
```

```
with(fa.FE, shapiro.test(sqrtF[MomTrmt == "Induced"]))
```

```
##
##  Shapiro-Wilk normality test
##
## data:  sqrtF[MomTrmt == "Induced"]
## W = 0.90632, p-value = 0.291
```

Using non-parametric

```
fa.fe.wilcox <- wilcox.test(AvFungus ~ MomTrmt, data = fa.FE)
```

```
## Warning in wilcox.test.default(x = c(4.6, 3.866666667, 4.533333333,
## 2.733333333, : cannot compute exact p-value with ties
```

```
fa.fe.wilcox
```

```
##
##  Wilcoxon rank sum test with continuity correction
##
## data:  AvFungus by MomTrmt
## W = 62.5, p-value = 0.01209
## alternative hypothesis: true location shift is not equal to 0
```

# Neocosmospora

```
fa.HM <- fa[fa$Fungus == "HM",]

fa.hm.lm <- lm(AvFungus ~ MomTrmt, data = fa.HM)

densityplot(~ residuals(fa.hm.lm), group = MomTrmt, data = fa.HM, auto.key=TRUE)
```

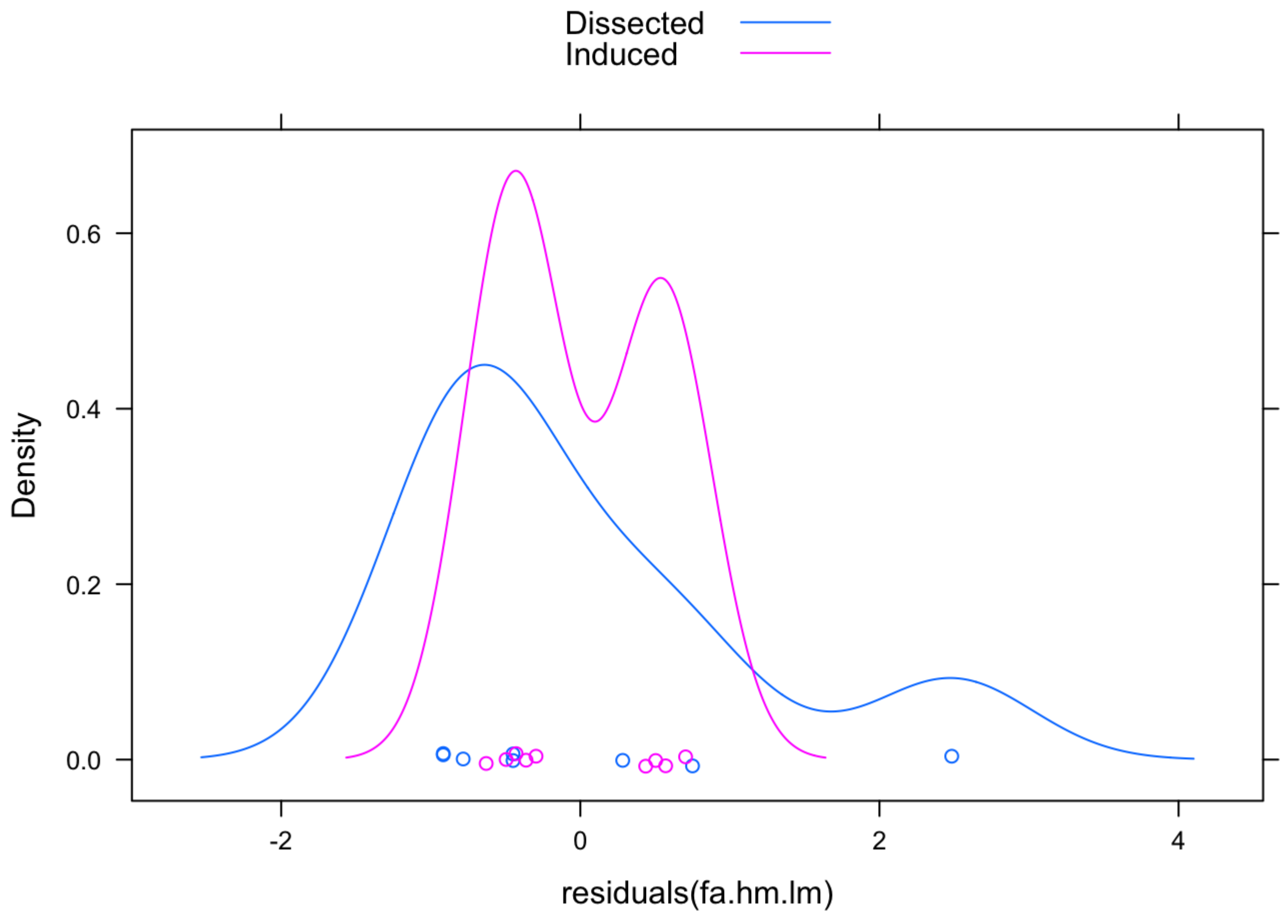


```
with(fa.HM, shapiro.test(AvFungus[MomTrmt == "Dissected"]))
```

```
##
##  Shapiro-Wilk normality test
##
## data:  AvFungus[MomTrmt == "Dissected"]
## W = 0.8076, p-value = 0.0345
```

```
with(fa.HM, shapiro.test(AvFungus[MomTrmt == "Induced"]))
```

```
##
##  Shapiro-Wilk normality test
##
## data:  AvFungus[MomTrmt == "Induced"]
## W = 0.83785, p-value = 0.05465
```

Log transform

```
fa.HM.lm.log <- lm(LogFungus ~ MomTrmt, data = fa.HM)

densityplot(~ residuals(fa.HM.lm.log), group = MomTrmt, data = fa.HM, auto.key=TRUE)
```

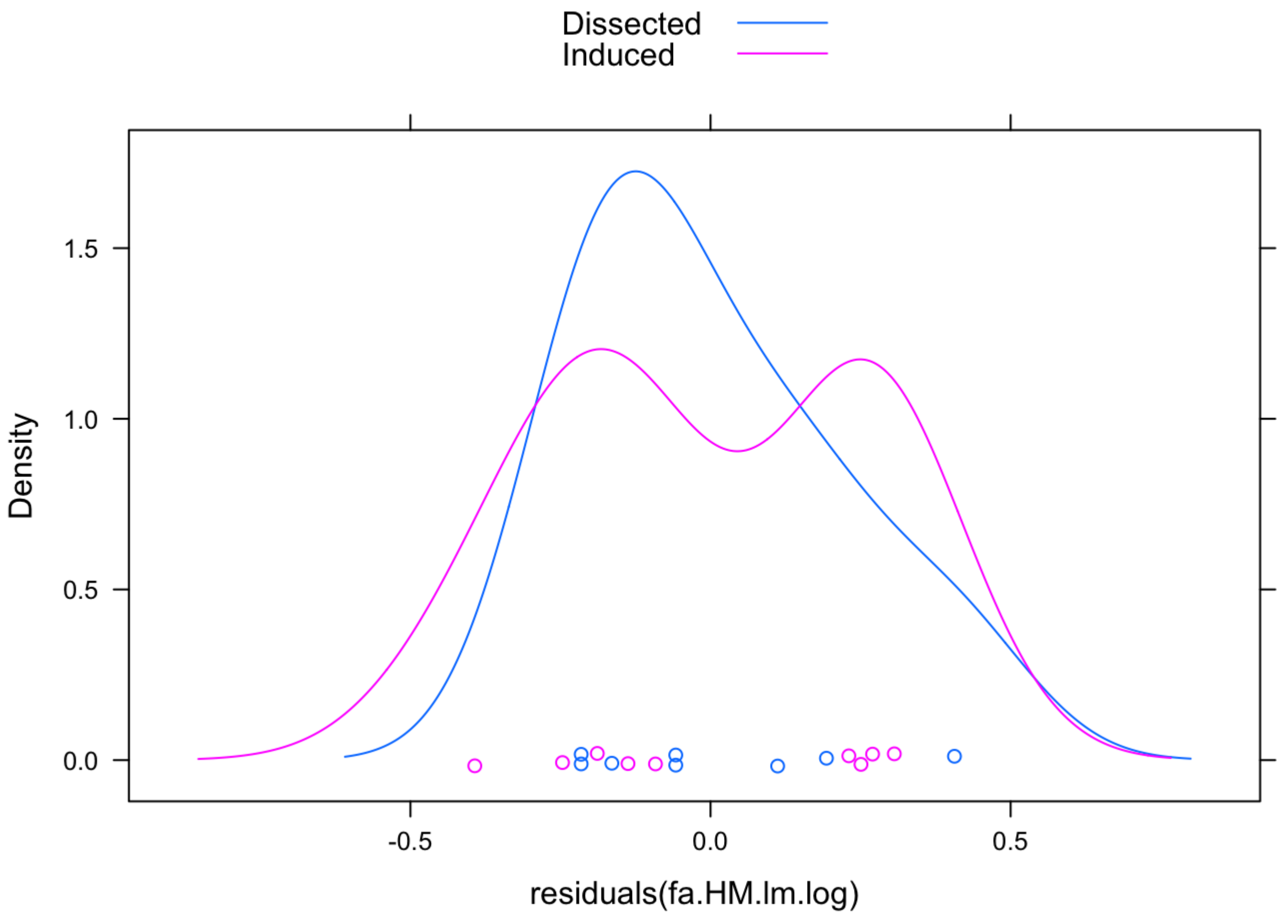


```
with(fa.HM, shapiro.test(LogFungus[MomTrmt == "Dissected"]))
```

```
##
##  Shapiro-Wilk normality test
##
## data:  LogFungus[MomTrmt == "Dissected"]
## W = 0.89824, p-value = 0.2786
```

```
with(fa.HM, shapiro.test(LogFungus[MomTrmt == "Induced"]))
```

```
##
##  Shapiro-Wilk normality test
##
## data:  LogFungus[MomTrmt == "Induced"]
## W = 0.87665, p-value = 0.1447
```

```
fa.hm.ttest <- t.test(LogFungus ~ MomTrmt, data = fa.HM, var.equal = TRUE)
fa.hm.ttest
```

```
##
##  Two Sample t-test
##
## data:  LogFungus by MomTrmt
## t = 2.7516, df = 15, p-value = 0.01484
## alternative hypothesis: true difference in means is not equal to 0
## 95 percent confidence interval:
##  0.07390755 0.58192768
## sample estimates:
## mean in group Dissected   mean in group Induced
##              0.24357196             -0.08434566
```

# Hatch Success

## Viable eggs at day 25

All sterile oviposited eggs were viable at day 25, so there is not statistic we can do including incubation environment with no variation in one category. Only looking at eggs incubated in fungal inoculated environment and examining for an effect of egg type

```
gh.d <- gh[gh$mom.treatment == "dissected",]
```

```
viable.glm <- glmer(cbind(not.viable,viable.d25) ~ egg.treatment + (1|mother.toe.clip), family =
"binomial", data = gh.d)

summary(viable.glm)
```

```
## Generalized linear mixed model fit by maximum likelihood (Laplace
##   Approximation) [glmerMod]
##  Family: binomial  ( logit )
## Formula: cbind(not.viable, viable.d25) ~ egg.treatment + (1 | mother.toe.clip)
##    Data: gh.d
## 
##      AIC      BIC   logLik deviance df.resid 
##     55.9     59.2    -25.0     49.9       19 
## 
## Scaled residuals: 
##     Min      1Q  Median      3Q     Max 
## -1.6139 -0.7108 -0.1519  0.7990  1.9767 
## 
## Random effects:
##  Groups          Name        Variance Std.Dev.
##  mother.toe.clip (Intercept) 0.09843  0.3137  
## Number of obs: 22, groups:  mother.toe.clip, 11
## 
## Fixed effects:
##                      Estimate Std. Error z value Pr(>|z|)  
## (Intercept)           -0.4487     0.3388  -1.324   0.1854  
## egg.treatmentsterile  -1.3602     0.5757  -2.363   0.0181 *
## ---
## Signif. codes:  0 '***' 0.001 '**' 0.01 '*' 0.05 '.' 0.1 ' ' 1
## 
## Correlation of Fixed Effects:
##             (Intr)
## egg.trtmnts -0.529
```

# Hatched Eggs

```
gh.hatch <- gh[gh$mother.toe.clip != "7"& gh$mother.toe.clip != "3",]
```

Removed two individuals which did not have an egg in each incubation environment

Model selection:

```
hatch.glm <- glmer(cbind(not.hatched,hatched) ~ mom.treatment * egg.treatment + (1|mother.toe.cl
ip), family = "binomial", data = gh.hatch)

hatch.glm.a <- glmer(cbind(not.hatched,hatched) ~ mom.treatment * egg.treatment + (egg.treatment
|mother.toe.clip), family = "binomial", data = gh.hatch)

anova (hatch.glm, hatch.glm.a)
```

```
## Data: gh.hatch
## Models:
## hatch.glm: cbind(not.hatched, hatched) ~ mom.treatment * egg.treatment +
## hatch.glm:     (1 | mother.toe.clip)
## hatch.glm.a: cbind(not.hatched, hatched) ~ mom.treatment * egg.treatment +
## hatch.glm.a:     (egg.treatment | mother.toe.clip)
##             npar    AIC    BIC  logLik deviance  Chisq Df Pr(>Chisq)
## hatch.glm      5 83.990 92.178 -36.995   73.990
## hatch.glm.a    7 87.875 99.339 -36.938   73.875 0.1146  2     0.9443
```

Continuing model selection using random effect modeled as (1|mother.toe.clip) based on AIC of hatch.glm vs hatch.glm.a

```
hatch.glm1 <- glmer(cbind(not.hatched,hatched) ~ mom.treatment + egg.treatment + (1|mother.toe.c
lip), family = "binomial", data = gh.hatch)

hatch.glm2 <- glmer(cbind(not.hatched,hatched) ~ mom.treatment + (1|mother.toe.clip), family =
"binomial", data = gh.hatch)

hatch.glm3 <- glmer(cbind(not.hatched,hatched) ~ egg.treatment + (1|mother.toe.clip), family =
"binomial", data = gh.hatch)

hatch.glm.null <- glmer(cbind(not.hatched,hatched) ~ (1|mother.toe.clip), family = "binomial", d
ata = gh.hatch)

anova(hatch.glm,hatch.glm.a, hatch.glm1, hatch.glm2, hatch.glm3, hatch.glm.null)
```

```
## Data: gh.hatch
## Models:
## hatch.glm.null: cbind(not.hatched, hatched) ~ (1 | mother.toe.clip)
## hatch.glm2: cbind(not.hatched, hatched) ~ mom.treatment + (1 | mother.toe.clip)
## hatch.glm3: cbind(not.hatched, hatched) ~ egg.treatment + (1 | mother.toe.clip)
## hatch.glm1: cbind(not.hatched, hatched) ~ mom.treatment + egg.treatment +
## hatch.glm1:     (1 | mother.toe.clip)
## hatch.glm: cbind(not.hatched, hatched) ~ mom.treatment * egg.treatment +
## hatch.glm:     (1 | mother.toe.clip)
## hatch.glm.a: cbind(not.hatched, hatched) ~ mom.treatment * egg.treatment +
## hatch.glm.a:     (egg.treatment | mother.toe.clip)
##                npar    AIC    BIC  logLik deviance   Chisq Df Pr(>Chisq)
## hatch.glm.null    2 94.366 97.641 -45.183   90.366
## hatch.glm2        3 82.792 87.705 -38.396   76.792 13.5740  1  0.0002293 ***
## hatch.glm3        3 94.721 99.634 -44.361   88.721  0.0000  0  1.0000000
## hatch.glm1        4 83.265 89.815 -37.632   75.265 13.4565  1  0.0002442 ***
## hatch.glm         5 83.990 92.178 -36.995   73.990  1.2747  1  0.2588819
## hatch.glm.a       7 87.875 99.339 -36.938   73.875  0.1146  2  0.9443084
## ---
## Signif. codes:  0 '***' 0.001 '**' 0.01 '*' 0.05 '.' 0.1 ' ' 1
```

hatch.glm2 has lowest AIC

```
summary(hatch.glm2)
```

```
## Generalized linear mixed model fit by maximum likelihood (Laplace
##   Approximation) [glmerMod]
##  Family: binomial  ( logit )
## Formula: cbind(not.hatched, hatched) ~ mom.treatment + (1 | mother.toe.clip)
##    Data: gh.hatch
## 
##      AIC      BIC   logLik deviance df.resid 
##     82.8     87.7    -38.4     76.8       35 
## 
## Scaled residuals: 
##     Min      1Q  Median      3Q     Max 
## -1.8002 -0.6051 -0.2338  0.7690  2.7282 
## 
## Random effects:
##  Groups          Name        Variance Std.Dev.
##  mother.toe.clip (Intercept) 0.02785  0.1669  
## Number of obs: 38, groups:  mother.toe.clip, 19
## 
## Fixed effects:
##                      Estimate Std. Error z value Pr(>|z|)    
## (Intercept)           -0.3967     0.2323  -1.708 0.087621 .  
## mom.treatmentinduced  -1.8992     0.5427  -3.500 0.000466 ***
## ---
## Signif. codes:  0 '***' 0.001 '**' 0.01 '*' 0.05 '.' 0.1 ' ' 1
## 
## Correlation of Fixed Effects:
##             (Intr)
## mm.trtmntnd -0.411
```

```
confint(hatch.glm2)
```

```
## Computing profile confidence intervals ...
```

```
##                           2.5 %     97.5 %
## .sig01                0.0000000  1.1085070
## (Intercept)          -0.9463398  0.1080154
## mom.treatmentinduced -3.2167488 -0.9363014
```

```
plot(hatch.glm2)
```

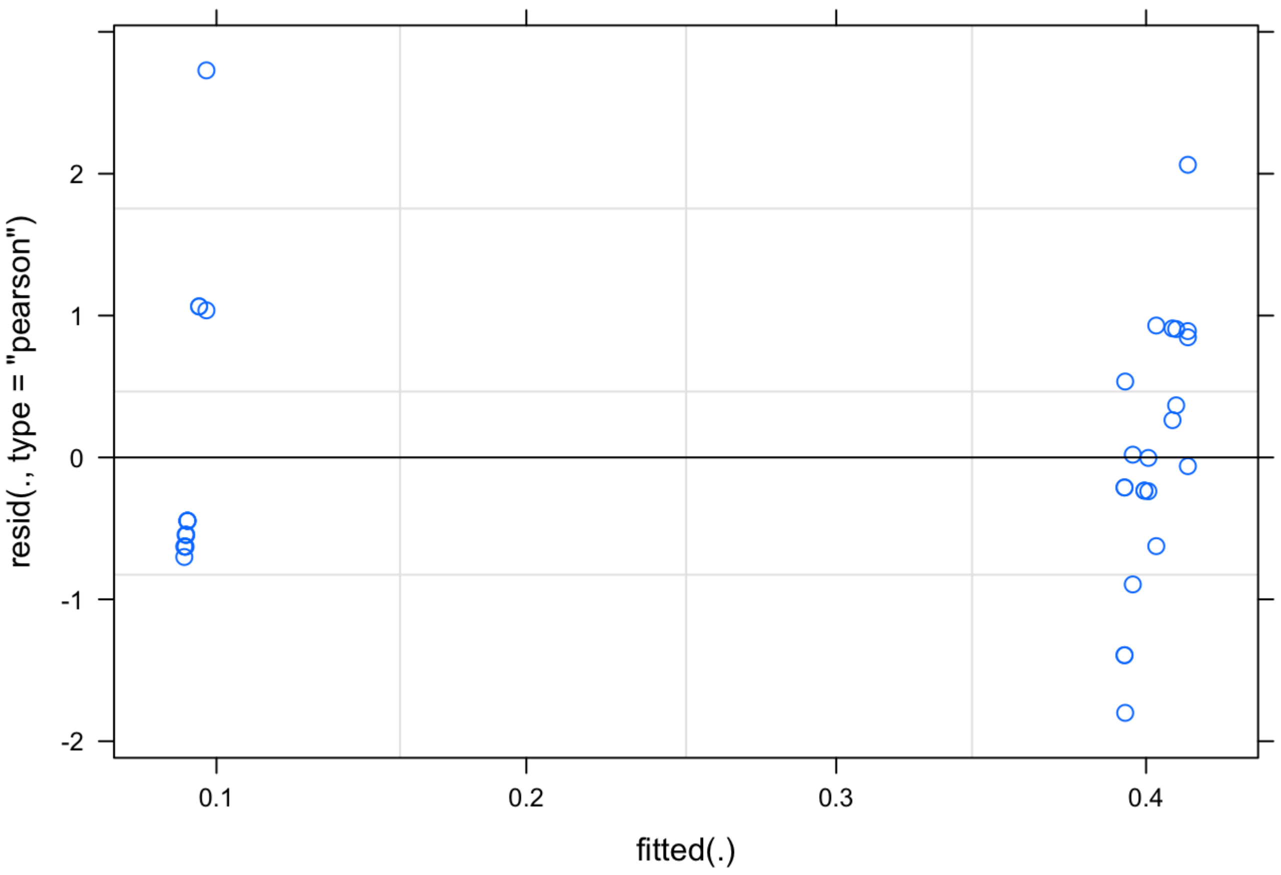


# Hatchling Demographics

## Incubation time

Model selection

```
inc.lmer <- lmer(inc.time ~ MomTrmt * EggTrmt + (1|MomTC), data = babes)
inc.lmer.a <- lmer(inc.time ~ MomTrmt * EggTrmt + (EggTrmt|MomTC), data = babes)
```

```
## boundary (singular) fit: see ?isSingular
```

```
anova(inc.lmer, inc.lmer.a)
```

```
## refitting model(s) with ML (instead of REML)
```

```
## Data: babes
## Models:
## inc.lmer: inc.time ~ MomTrmt * EggTrmt + (1 | MomTC)
## inc.lmer.a: inc.time ~ MomTrmt * EggTrmt + (EggTrmt | MomTC)
##            npar    AIC    BIC  logLik deviance Chisq Df Pr(>Chisq)
## inc.lmer      6 409.93 425.25 -198.97   397.93
## inc.lmer.a    8 411.25 431.68 -197.63   395.25 2.677  2     0.2622
```

Continuing model selection using random effect modeled as (1|MomTC) based on AIC of inc.lmer vs inc.lmer.a Also, inc.lmer.a gives boundary (singular) fit error. This is true for all response variables modeled below and so was excluded from script from here on out.

```
inc.lmer1 <- lmer(inc.time ~ MomTrmt + EggTrmt + (1|MomTC), data = babes)
inc.lmer2 <- lmer(inc.time ~ MomTrmt + (1|MomTC), data = babes)
inc.lmer3 <- lmer(inc.time ~ EggTrmt + (1|MomTC), data = babes)
inc.lmer.null <- lmer(inc.time ~ (1|MomTC), data = babes)

anova(inc.lmer, inc.lmer1, inc.lmer2, inc.lmer3, inc.lmer.null)
```

```
## refitting model(s) with ML (instead of REML)
```

```
## Data: babes
## Models:
## inc.lmer.null: inc.time ~ (1 | MomTC)
## inc.lmer2: inc.time ~ MomTrmt + (1 | MomTC)
## inc.lmer3: inc.time ~ EggTrmt + (1 | MomTC)
## inc.lmer1: inc.time ~ MomTrmt + EggTrmt + (1 | MomTC)
## inc.lmer: inc.time ~ MomTrmt * EggTrmt + (1 | MomTC)
##               npar    AIC    BIC  logLik deviance  Chisq Df Pr(>Chisq)
## inc.lmer.null    3 410.26 417.92 -202.13   404.26
## inc.lmer2        4 411.84 422.05 -201.92   403.84 0.4241  1    0.51492
## inc.lmer3        4 412.23 422.45 -202.12   404.23 0.0000  0    1.00000
## inc.lmer1        5 413.81 426.58 -201.91   403.81 0.4202  1    0.51682
## inc.lmer         6 409.93 425.25 -198.97   397.93 5.8836  1    0.01528 *
## ---
## Signif. codes:  0 '***' 0.001 '**' 0.01 '*' 0.05 '.' 0.1 ' ' 1
```

Lowest AIC model = inc.lmer

```
inc.lmer <- lmer(inc.time ~ MomTrmt * EggTrmt + (1|MomTC), data = babes)
summary(inc.lmer)
```

```
## Linear mixed model fit by REML. t-tests use Satterthwaite's method [
## lmerModLmerTest]
## Formula: inc.time ~ MomTrmt * EggTrmt + (1 | MomTC)
##    Data: babes
##
## REML criterion at convergence: 394.4
##
## Scaled residuals:
##      Min       1Q   Median       3Q      Max
## -2.30627 -0.43873  0.07794  0.53319  2.77660
##
## Random effects:
##  Groups   Name        Variance Std.Dev.
##  MomTC    (Intercept) 6.045    2.459
##  Residual             2.477    1.574
## Number of obs: 95, groups:  MomTC, 18
##
## Fixed effects:
##                              Estimate Std. Error      df t value Pr(>|t|)
## (Intercept)                   45.1856     0.8563 19.5783  52.768   <2e-16 ***
## MomTrmtInduced                -1.5764     1.2615 18.2712  -1.250   0.2272
## EggTrmtsterile                -0.7930     0.4783 75.6540  -1.658   0.1014
## MomTrmtInduced:EggTrmtsterile   1.6085     0.6578 75.3288   2.445   0.0168 *
## ---
## Signif. codes:  0 '***' 0.001 '**' 0.01 '*' 0.05 '.' 0.1 ' ' 1
##
## Correlation of Fixed Effects:
##             (Intr) MmTrmI EggTrm
## MmTrmtIndcd -0.679
## EggTrmtstrl -0.302  0.205
## MmTrmtIn:ET  0.220 -0.268 -0.727
```

```
confint(inc.lmer)
```

```
## Computing profile confidence intervals ...
```

```
##                                    2.5 %     97.5 %
## .sig01                         1.6432627  3.4159797
## .sigma                         1.3369376  1.8358060
## (Intercept)                   43.5281858 46.8512021
## MomTrmtInduced                -4.0316863  0.8712233
## EggTrmtsterile                -1.7301208  0.1431492
## MomTrmtInduced:EggTrmtsterile  0.3173424  2.8944736
```

```
plot(inc.lmer)
```

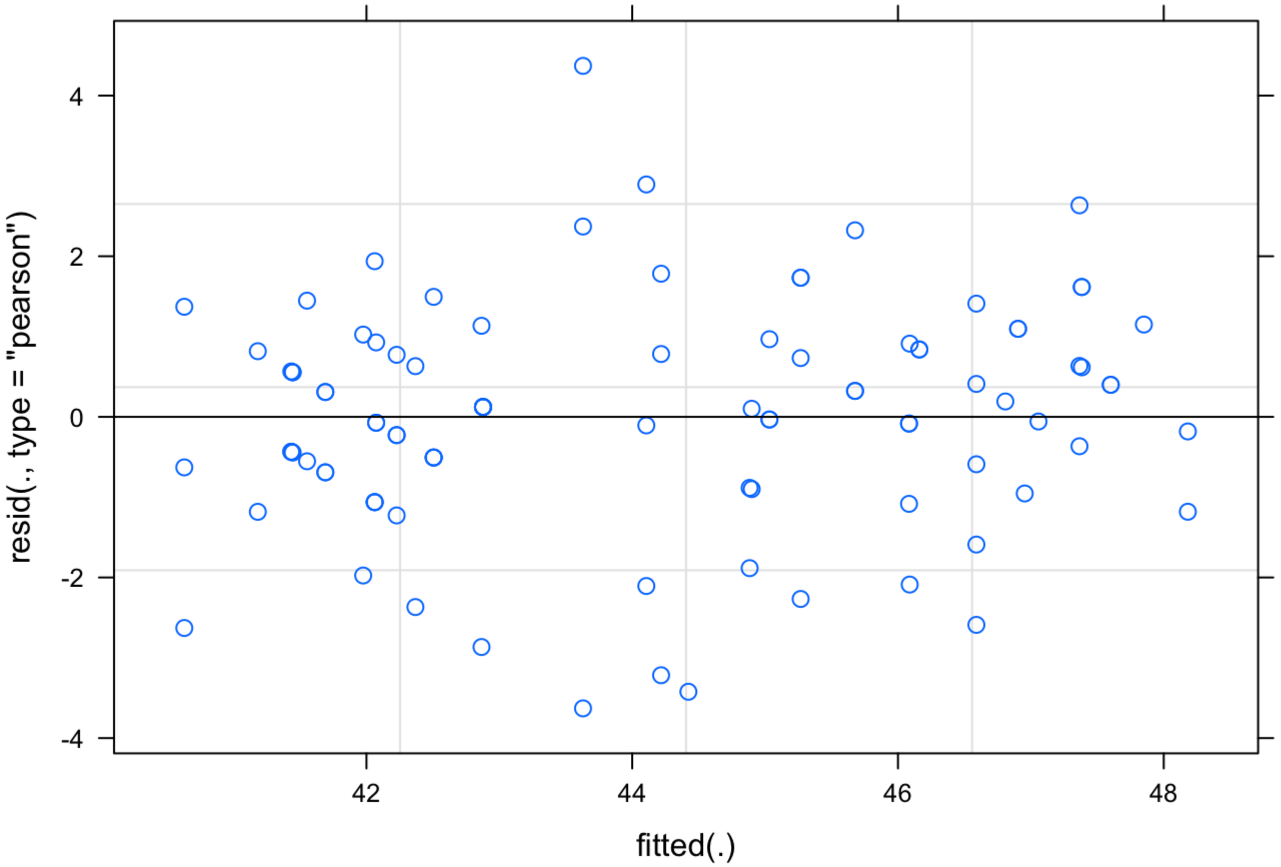


Significant interaction term. Looking at incubation environments separately for effect of egg type on incubation time

```
babes.fungal <- babes[babes$EggTrmt == "fungus",]
babes.sterile <- babes[babes$EggTrmt == "sterile",]
```

Sterile Environment

```
inc.sterile <- lmer(inc.time ~ MomTrmt + (1|MomTC), data = babes.sterile)
summary(inc.sterile)
```

```
## Linear mixed model fit by REML. t-tests use Satterthwaite's method [
## lmerModLmerTest]
## Formula: inc.time ~ MomTrmt + (1 | MomTC)
##    Data: babes.sterile
##
## REML criterion at convergence: 208.2
##
## Scaled residuals:
##     Min      1Q  Median      3Q     Max
## -3.0319 -0.4344 -0.0290  0.4741  2.2209
##
## Random effects:
##  Groups   Name        Variance Std.Dev.
##  MomTC    (Intercept) 4.712    2.171
##  Residual             2.319    1.523
## Number of obs: 49, groups:  MomTC, 18
##
## Fixed effects:
##                Estimate Std. Error       df t value Pr(>|t|)
## (Intercept)    44.31834    0.76296 15.78172  58.087   <2e-16 ***
## MomTrmtInduced  0.02638    1.12983 15.09280   0.023    0.982
## ---
## Signif. codes:  0 '***' 0.001 '**' 0.01 '*' 0.05 '.' 0.1 ' ' 1
##
## Correlation of Fixed Effects:
##             (Intr)
## MmTrmtIndcd -0.675
```

```
confint(inc.sterile)
```

```
## Computing profile confidence intervals ...
```

```
##                    2.5 %    97.5 %
## .sig01          1.281788  3.105479
## .sigma          1.209294  2.009735
## (Intercept)    42.838056 45.813264
## MomTrmtInduced -2.179912  2.227073
```

```
plot(inc.sterile)
```

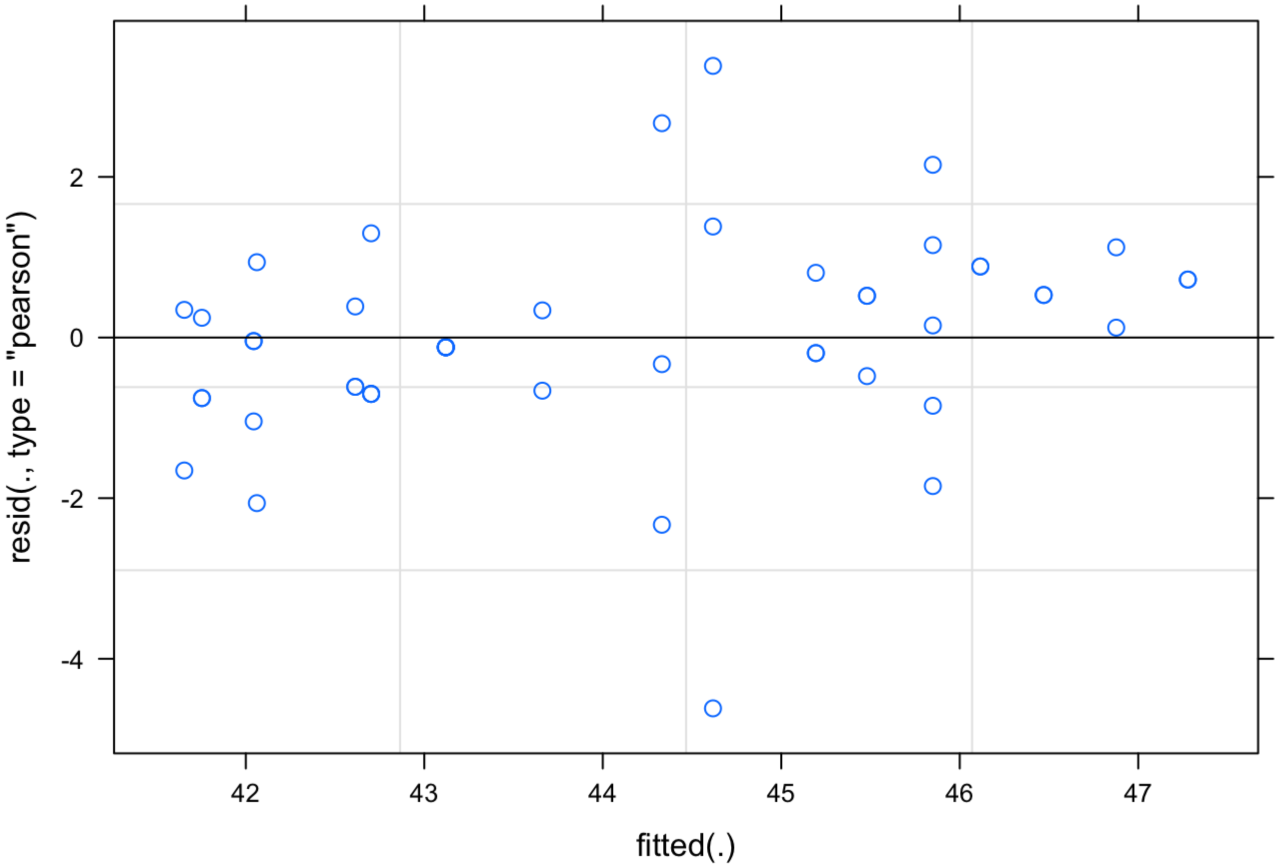


```
inc.s.null<-lmer(inc.time~(1|MomTC), data = babes.sterile)
anova(inc.s.null, inc.sterile)
```

```
## refitting model(s) with ML (instead of REML)
```

```
## Data: babes.sterile
## Models:
## inc.s.null: inc.time ~ (1 | MomTC)
## inc.sterile: inc.time ~ MomTrmt + (1 | MomTC)
##             npar    AIC    BIC  logLik deviance Chisq Df Pr(>Chisq)
## inc.s.null     3 216.85 222.53 -105.43   210.85
## inc.sterile    4 218.85 226.42 -105.43   210.85 8e-04  1     0.9776
```

Fungal Environment

```
inc.fungal <- lmer(inc.time ~ MomTrmt + (1|MomTC), data = babes.fungal)
summary(inc.fungal)
```

```
## Linear mixed model fit by REML. t-tests use Satterthwaite's method [
## lmerModLmerTest]
## Formula: inc.time ~ MomTrmt + (1 | MomTC)
##    Data: babes.fungal
##
## REML criterion at convergence: 205
##
## Scaled residuals:
##     Min      1Q  Median      3Q     Max
## -1.8706 -0.5586  0.1258  0.5995  1.3476
##
## Random effects:
##  Groups   Name        Variance Std.Dev.
##  MomTC    (Intercept) 7.651    2.766
##  Residual             2.505    1.583
## Number of obs: 46, groups:  MomTC, 18
##
## Fixed effects:
##                Estimate Std. Error     df t value Pr(>|t|)
## (Intercept)      44.923      0.953 16.748  47.140   <2e-16 ***
## MomTrmtInduced   -1.326      1.404 15.673  -0.945    0.359
## ---
## Signif. codes:  0 '***' 0.001 '**' 0.01 '*' 0.05 '.' 0.1 ' ' 1
##
## Correlation of Fixed Effects:
##             (Intr)
## MmTrmtIndcd -0.679
```

```
anova(inc.fungal)
```

```
## Type III Analysis of Variance Table with Satterthwaite's method
##         Sum Sq Mean Sq NumDF  DenDF F value Pr(>F)
## MomTrmt  2.235   2.235     1 15.673  0.8924 0.3592
```

```
confint(inc.fungal)
```

```
## Computing profile confidence intervals ...
```

```
##                    2.5 %    97.5 %
## .sig01          1.763059  3.894440
## .sigma          1.243906  2.108391
## (Intercept)    43.067309 46.780266
## MomTrmtInduced -4.065634  1.412795
```

```
inc.f.null <- lmer(inc.time~(1|MomTC), data = babes.fungal)
anova(inc.f.null, inc.fungal)
```

```
## refitting model(s) with ML (instead of REML)
```

```
## Data: babes.fungal
## Models:
## inc.f.null: inc.time ~ (1 | MomTC)
## inc.fungal: inc.time ~ MomTrmt + (1 | MomTC)
##            npar    AIC    BIC  logLik deviance  Chisq Df Pr(>Chisq)
## inc.f.null    3 215.52 221.01 -104.76   209.52
## inc.fungal    4 216.54 223.86 -104.27   208.54 0.9784  1     0.3226
```

# Body Mass

Model Selection

```
bm.lmer <- lmer(HatchBmass ~ MomTrmt * EggTrmt + (1|MomTC), data = babes)
bm.lmer1 <- lmer(HatchBmass ~ MomTrmt + EggTrmt + (1|MomTC), data = babes)
bm.lmer2 <- lmer(HatchBmass ~ MomTrmt + (1|MomTC), data = babes)
bm.lmer3 <- lmer(HatchBmass ~ EggTrmt + (1|MomTC), data = babes)
bm.lmer.null <- lmer(HatchBmass ~ (1|MomTC), data = babes)

anova(bm.lmer, bm.lmer1, bm.lmer2, bm.lmer3, bm.lmer.null)
```

```
## refitting model(s) with ML (instead of REML)
```

```
## Data: babes
## Models:
## bm.lmer.null: HatchBmass ~ (1 | MomTC)
## bm.lmer2: HatchBmass ~ MomTrmt + (1 | MomTC)
## bm.lmer3: HatchBmass ~ EggTrmt + (1 | MomTC)
## bm.lmer1: HatchBmass ~ MomTrmt + EggTrmt + (1 | MomTC)
## bm.lmer: HatchBmass ~ MomTrmt * EggTrmt + (1 | MomTC)
##              npar     AIC     BIC logLik deviance  Chisq Df Pr(>Chisq)
## bm.lmer.null    3 -335.99 -328.33 171.00  -341.99
## bm.lmer2        4 -342.83 -332.62 175.42  -350.83 8.8426  1   0.002943 **
## bm.lmer3        4 -337.16 -326.94 172.58  -345.16 0.0000  0   1.000000
## bm.lmer1        5 -344.24 -331.47 177.12  -354.24 9.0848  1   0.002577 **
## bm.lmer         6 -344.24 -328.92 178.12  -356.24 1.9961  1   0.157704
## ---
## Signif. codes:  0 '***' 0.001 '**' 0.01 '*' 0.05 '.' 0.1 ' ' 1
```

bm.lmer and .lmer1 have lowest AIC. Use bm.lmer

```
require(lmerTest)
bm.lmer <- lmer(HatchBmass ~ MomTrmt * EggTrmt + (1|MomTC), data = babes)
summary(bm.lmer)
```

```
## Linear mixed model fit by REML. t-tests use Satterthwaite's method [
## lmerModLmerTest]
## Formula: HatchBmass ~ MomTrmt * EggTrmt + (1 | MomTC)
##    Data: babes
##
## REML criterion at convergence: -326.9
##
## Scaled residuals:
##     Min      1Q  Median      3Q     Max
## -3.5851 -0.5202  0.0736  0.5315  2.9057
##
## Random effects:
##  Groups   Name        Variance  Std.Dev.
##  MomTC    (Intercept) 0.0009125 0.03021
##  Residual             0.0010468 0.03235
## Number of obs: 95, groups:  MomTC, 18
##
## Fixed effects:
##                                Estimate Std. Error        df t value Pr(>|t|)
## (Intercept)                    0.360867   0.012040 24.455661  29.972   <2e-16
## MomTrmtInduced                 0.042010   0.017383 21.311097   2.417   0.0247
## EggTrmtsterile                 0.002553   0.009796 76.303605   0.261   0.7951
## MomTrmtInduced:EggTrmtsterile  0.018942   0.013494 75.546616   1.404   0.1645
##
## (Intercept)                   ***
## MomTrmtInduced                *
## EggTrmtsterile
## MomTrmtInduced:EggTrmtsterile
## ---
## Signif. codes:  0 '***' 0.001 '**' 0.01 '*' 0.05 '.' 0.1 ' ' 1
##
## Correlation of Fixed Effects:
##             (Intr) MmTrmI EggTrm
## MmTrmtIndcd -0.693
## EggTrmtstrl -0.441  0.305
## MmTrmtIn:ET  0.320 -0.400 -0.726
```

```
confint(bm.lmer)
```

```
## Computing profile confidence intervals ...
```

```
##                                      2.5 %     97.5 %
## .sig01                         0.017967140 0.04355329
## .sigma                         0.027481499 0.03778013
## (Intercept)                    0.337617511 0.38409150
## MomTrmtInduced                 0.008386092 0.07564024
## EggTrmtsterile                -0.016586913 0.02178332
## MomTrmtInduced:EggTrmtsterile -0.007493599 0.04537880
```

```
plot(bm.lmer)
```

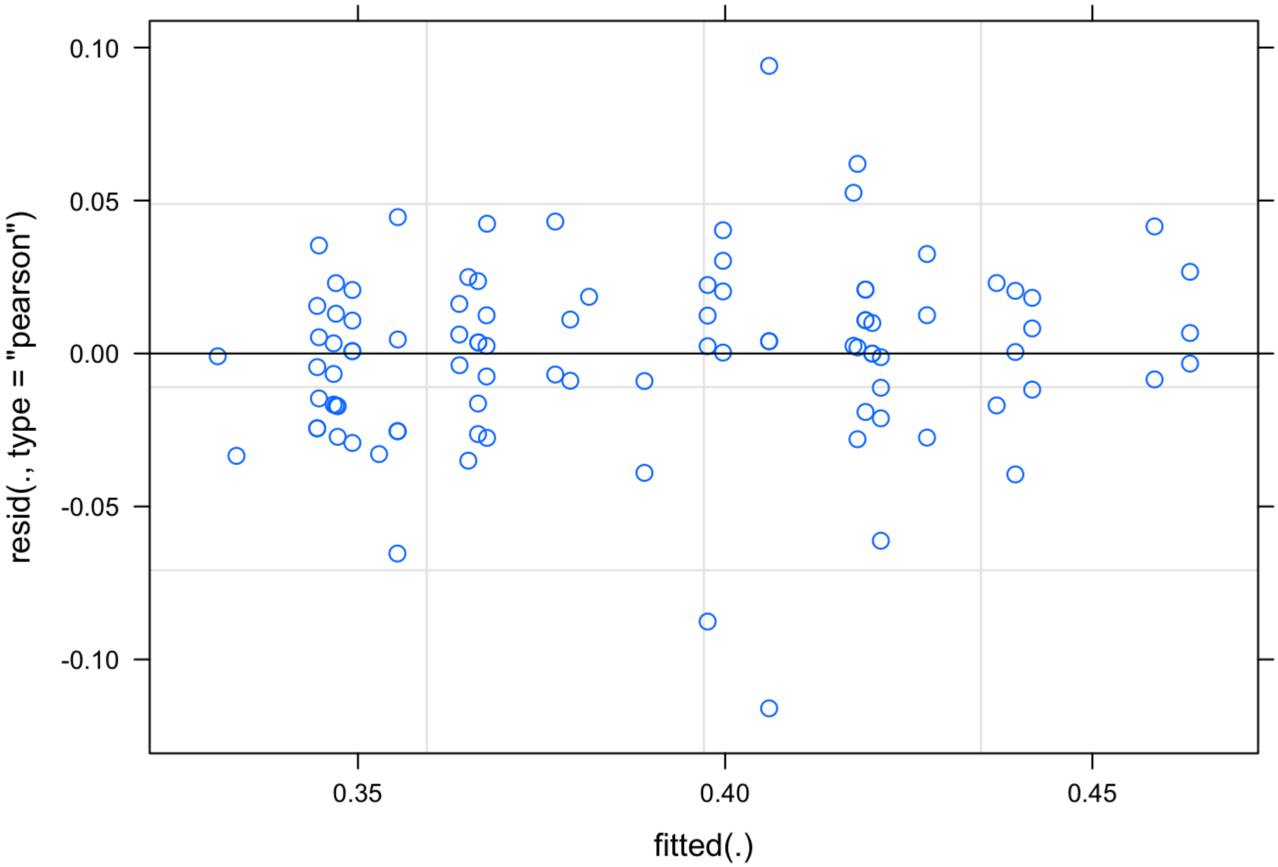


# Snout-vent length

Model Selection

```
svl.lmer <- lmer(HatchSVL ~ MomTrmt * EggTrmt + (1|MomTC), data = babes)
svl.lmer1 <- lmer(HatchSVL ~ MomTrmt + EggTrmt + (1|MomTC), data = babes)
svl.lmer2 <- lmer(HatchSVL ~ MomTrmt + (1|MomTC), data = babes)
svl.lmer3 <- lmer(HatchSVL ~ EggTrmt + (1|MomTC), data = babes)
svl.lmer.null <- lmer(HatchSVL ~ (1|MomTC), data = babes)

anova(svl.lmer, svl.lmer1, svl.lmer2, svl.lmer3, svl.lmer.null)
```

```
## refitting model(s) with ML (instead of REML)
```

```
## Data: babes
## Models:
## svl.lmer.null: HatchSVL ~ (1 | MomTC)
## svl.lmer2: HatchSVL ~ MomTrmt + (1 | MomTC)
## svl.lmer3: HatchSVL ~ EggTrmt + (1 | MomTC)
## svl.lmer1: HatchSVL ~ MomTrmt + EggTrmt + (1 | MomTC)
## svl.lmer: HatchSVL ~ MomTrmt * EggTrmt + (1 | MomTC)
##               npar    AIC    BIC  logLik deviance  Chisq Df Pr(>Chisq)
## svl.lmer.null    3 248.62 256.28 -121.31   242.62
## svl.lmer2        4 241.97 252.19 -116.99   233.97 8.6474  1   0.003275 **
## svl.lmer3        4 250.58 260.80 -121.29   242.58 0.0000  0   1.000000
## svl.lmer1        5 243.96 256.73 -116.98   233.96 8.6261  1   0.003314 **
## svl.lmer         6 236.34 251.66 -112.17   224.34 9.6192  1   0.001926 **
## ---
## Signif. codes:  0 '***' 0.001 '**' 0.01 '*' 0.05 '.' 0.1 ' ' 1
```

svl.lmer has lowest AIC

```
svl.lmer <- lmer(HatchSVL ~ MomTrmt * EggTrmt + (1|MomTC), data = babes)
summary(svl.lmer)
```

```
## Linear mixed model fit by REML. t-tests use Satterthwaite's method [
## lmerModLmerTest]
## Formula: HatchSVL ~ MomTrmt * EggTrmt + (1 | MomTC)
##    Data: babes
##
## REML criterion at convergence: 229.4
##
## Scaled residuals:
##      Min       1Q   Median       3Q      Max
## -2.09659 -0.70082 -0.00352  0.60783  3.04216
##
## Random effects:
##  Groups   Name        Variance Std.Dev.
##  MomTC    (Intercept) 0.3514   0.5928
##  Residual             0.4853   0.6967
## Number of obs: 95, groups:  MomTC, 18
##
## Fixed effects:
##                                Estimate Std. Error      df t value Pr(>|t|)
## (Intercept)                     23.2786     0.2449 24.8205  95.060  < 2e-16 ***
## MomTrmtInduced                   0.5332     0.3520 21.2968   1.515  0.14452
## EggTrmtsterile                  -0.5035     0.2107 75.8512  -2.389  0.01936 *
## MomTrmtInduced:EggTrmtsterile   0.9169     0.2904 74.9569   3.157  0.00229 **
## ---
## Signif. codes:  0 '***' 0.001 '**' 0.01 '*' 0.05 '.' 0.1 ' ' 1
##
## Correlation of Fixed Effects:
##             (Intr) MmTrmI EggTrm
## MmTrmtIndcd -0.696
## EggTrmtstrl -0.466  0.324
## MmTrmtIn:ET  0.338 -0.425 -0.726
```

```
confint(svl.lmer)
```

```
## Computing profile confidence intervals ...
```

```
##                                   2.5 %      97.5 %
## .sig01                        0.3284325  0.86856007
## .sigma                        0.5915938  0.81501399
## (Intercept)                  22.8047265 23.74932239
## MomTrmtInduced               -0.1466391  1.21349920
## EggTrmtsterile               -0.9160102 -0.09016599
## MomTrmtInduced:EggTrmtsterile  0.3484652  1.48709029
```

```
plot(svl.lmer)
```

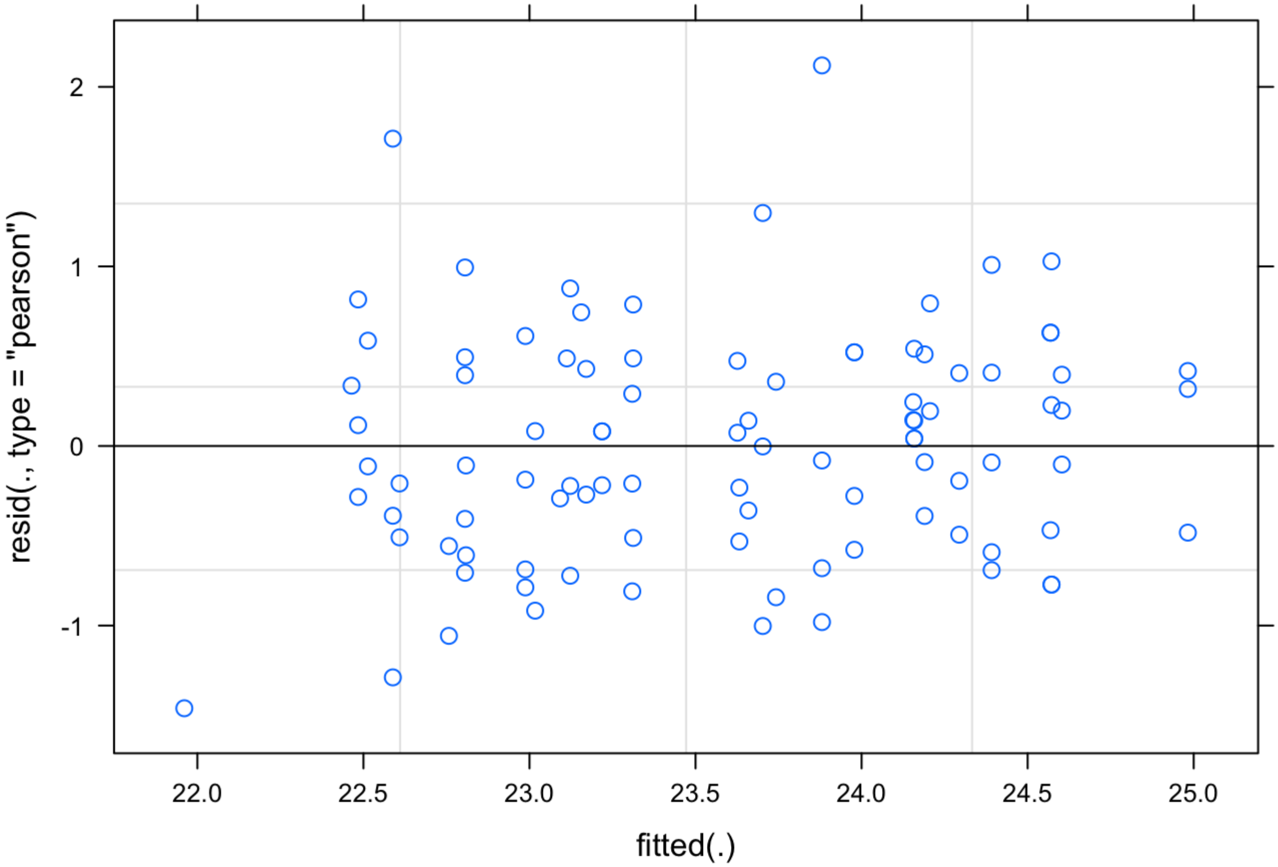


Significant interaction term. Looking at incubation environments separately for effect of egg type on hatchling SVL

Fungal Inoculated environment

Model Assumptions

```
svl.fungal <- lmer(HatchSVL ~ MomTrmt + (1|MomTC), data = babes.fungal)
summary(svl.fungal)
```

```
## Linear mixed model fit by REML. t-tests use Satterthwaite's method [
## lmerModLmerTest]
## Formula: HatchSVL ~ MomTrmt + (1 | MomTC)
##    Data: babes.fungal
##
## REML criterion at convergence: 113.9
##
## Scaled residuals:
##      Min       1Q   Median       3Q      Max
## -1.52771 -0.61732 -0.04858  0.65683  3.05570
##
## Random effects:
##  Groups   Name        Variance Std.Dev.
##  MomTC    (Intercept) 0.3718   0.6098
##  Residual             0.4575   0.6764
## Number of obs: 46, groups:  MomTC, 18
##
## Fixed effects:
##                Estimate Std. Error      df t value Pr(>|t|)
## (Intercept)     23.3383     0.2488 17.0771  93.789   <2e-16 ***
## MomTrmtInduced   0.4593     0.3572 14.7450   1.286    0.218
## ---
## Signif. codes:  0 '***' 0.001 '**' 0.01 '*' 0.05 '.' 0.1 ' ' 1
##
## Correlation of Fixed Effects:
##             (Intr)
## MmTrmtIndcd -0.697
```

```
confint(svl.fungal)
```

```
## Computing profile confidence intervals ...
```

```
##                     2.5 %     97.5 %
## .sig01          0.1640441  0.9319543
## .sigma          0.5317788  0.9071923
## (Intercept)    22.8547582 23.8219905
## MomTrmtInduced -0.2421561  1.1522553
```

```
plot(svl.fungal)
```

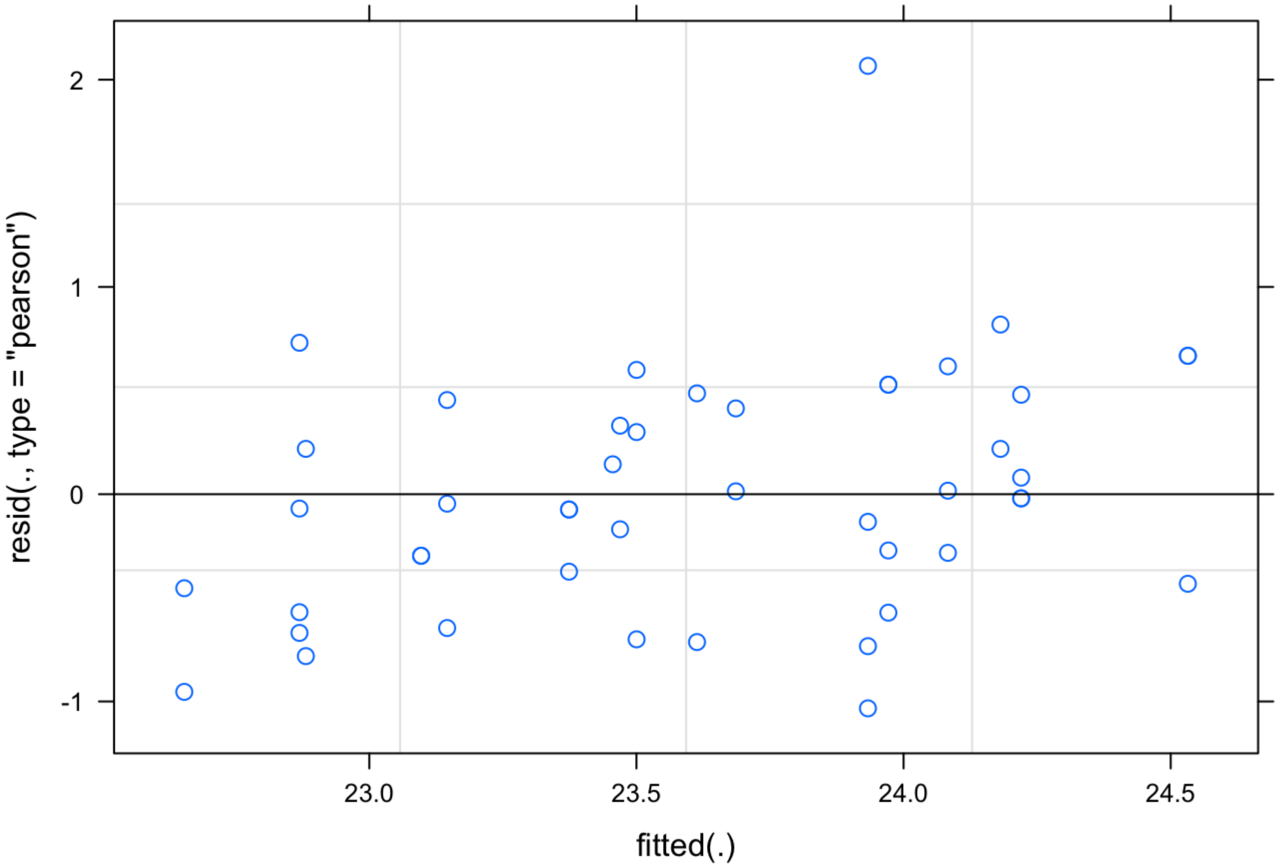


Sterile environment

```
svl.sterile <- lmer(HatchSVL ~ MomTrmt + (1|MomTC), data = babes.sterile)
summary(svl.sterile)
```

```
## Linear mixed model fit by REML. t-tests use Satterthwaite's method [
## lmerModLmerTest]
## Formula: HatchSVL ~ MomTrmt + (1 | MomTC)
##    Data: babes.sterile
##
## REML criterion at convergence: 125.9
##
## Scaled residuals:
##      Min       1Q   Median       3Q      Max
## -2.40545 -0.65286 -0.05546  0.56341  2.28833
##
## Random effects:
##  Groups   Name        Variance Std.Dev.
##  MomTC    (Intercept) 0.1188   0.3446
##  Residual             0.6527   0.8079
## Number of obs: 49, groups:  MomTC, 18
##
## Fixed effects:
##                Estimate Std. Error      df t value Pr(>|t|)
## (Intercept)     22.7970     0.1991  8.5283 114.520 6.81e-15 ***
## MomTrmtInduced   1.4976     0.2876  7.6014   5.208 0.000959 ***
## ---
## Signif. codes:  0 '***' 0.001 '**' 0.01 '*' 0.05 '.' 0.1 ' ' 1
##
## Correlation of Fixed Effects:
##             (Intr)
## MmTrmtIndcd -0.692
```

```
confint(svl.sterile)
```

```
## Computing profile confidence intervals ...
```

```
##                     2.5 %     97.5 %
## .sig01          0.0000000  0.7288091
## .sigma          0.6351064  1.0487263
## (Intercept)    22.4022632 23.1744478
## MomTrmtInduced  0.9468535  2.0499774
```

```
plot(svl.sterile)
```

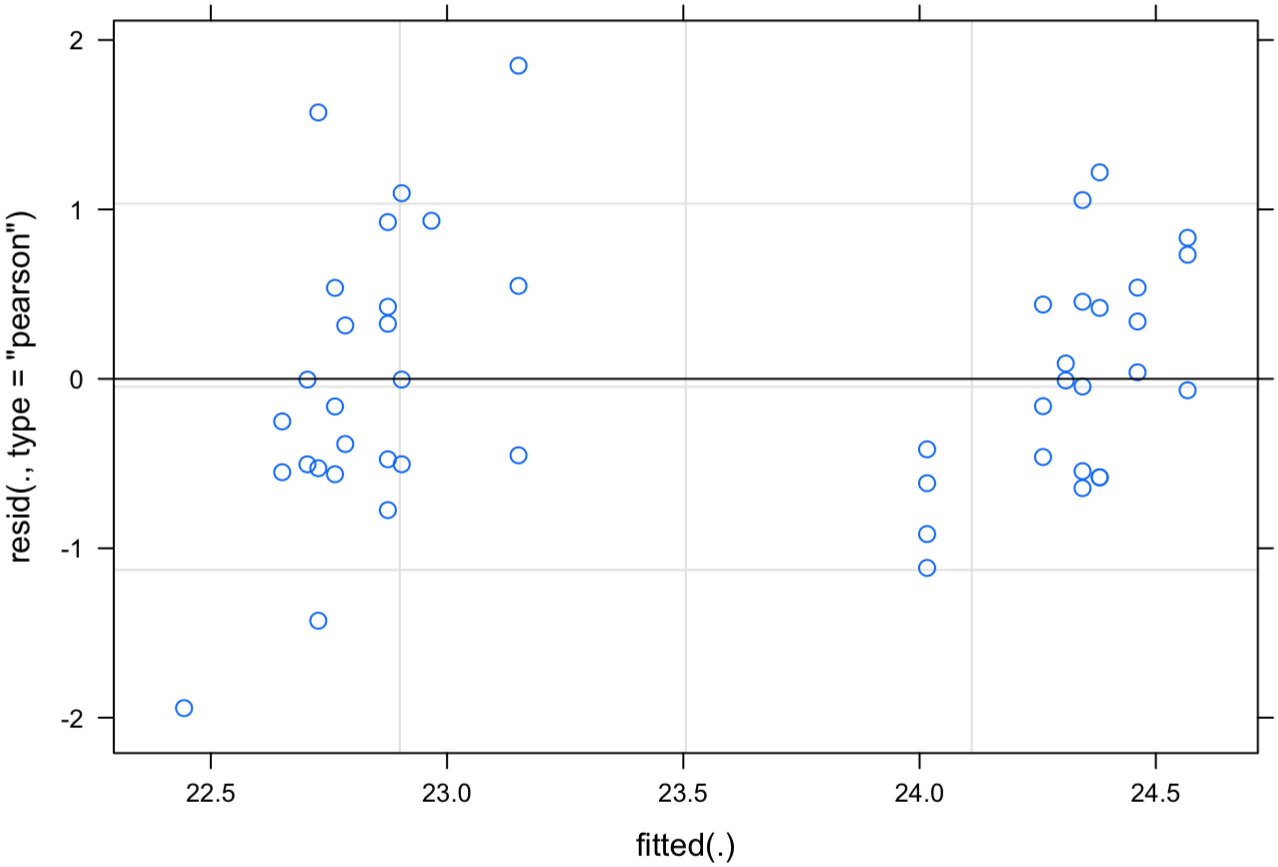


# 2017 Experimental data

## Hatch Success

```
EE17 <- read.csv("R_files/EE17_bymom.csv")
```

```
hatch.ee.glm <- glm(cbind(failed,hatched) ~ TRMT, family = "binomial", data = EE17)

summary(hatch.ee.glm)
```

```
##
## Call:
## glm(formula = cbind(failed, hatched) ~ TRMT, family = "binomial",
##     data = EE17)
##
## Deviance Residuals:
##     Min       1Q   Median       3Q      Max
## -1.5696  -1.0004   0.1856   0.8785   1.6604
##
## Coefficients:
##             Estimate Std. Error z value Pr(>|z|)
## (Intercept)   0.2657     0.2771   0.959    0.338
## TRMTInduced  -2.4903     0.5948  -4.187 2.83e-05 ***
## ---
## Signif. codes:  0 '***' 0.001 '**' 0.01 '*' 0.05 '.' 0.1 ' ' 1
##
## (Dispersion parameter for binomial family taken to be 1)
##
##     Null deviance: 38.476  on 13  degrees of freedom
## Residual deviance: 14.212  on 12  degrees of freedom
## AIC: 41.165
##
## Number of Fisher Scoring iterations: 5
```

# 2017 Hatchling Demographics

```
ee.babes <- read.csv("R_files/EE17_hatchedeggsonly_noA.csv")

ee.babes$MomTC <- as.factor(ee.babes$MomTC)
```

Incubation time

```
inc.lmer <- lmer(IncTime ~ MomTrmtGrp + (1|MomTC), data = ee.babes)
summary(inc.lmer)
```

```
## Linear mixed model fit by REML. t-tests use Satterthwaite's method [
## lmerModLmerTest]
## Formula: IncTime ~ MomTrmtGrp + (1 | MomTC)
##    Data: ee.babes
##
## REML criterion at convergence: 243.3
##
## Scaled residuals:
##     Min      1Q  Median      3Q     Max
## -3.2252 -0.4247  0.0959  0.5769  1.4587
##
## Random effects:
##  Groups   Name        Variance Std.Dev.
##  MomTC    (Intercept) 10.547   3.248
##  Residual              1.715   1.310
## Number of obs: 61, groups:  MomTC, 14
##
## Fixed effects:
##             Estimate Std. Error     df t value Pr(>|t|)
## (Intercept)   42.940      1.250 11.676  34.355 4.29e-13 ***
## MomTrmtGrpD    2.162      1.785 12.114   1.211    0.249
## ---
## Signif. codes:  0 '***' 0.001 '**' 0.01 '*' 0.05 '.' 0.1 ' ' 1
##
## Correlation of Fixed Effects:
##             (Intr)
## MomTrmtGrpD -0.700
```

```
plot(inc.lmer)
```

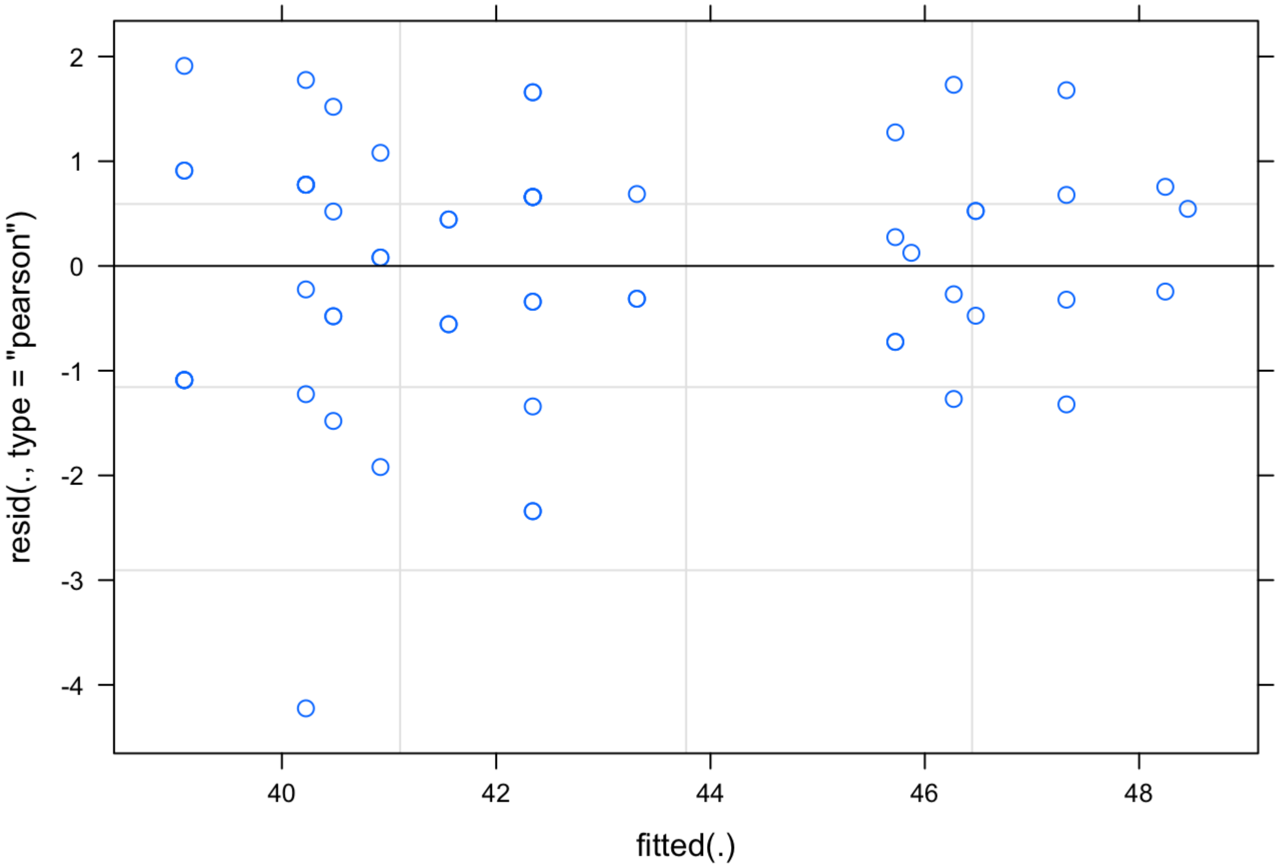


Body Mass

```
bmass.lmer <- lmer(hatchBmass ~ MomTrmtGrp + (1|MomTC), data = ee.babes)
summary(bmass.lmer)
```

```
## Linear mixed model fit by REML. t-tests use Satterthwaite's method [
## lmerModLmerTest]
## Formula: hatchBmass ~ MomTrmtGrp + (1 | MomTC)
##    Data: ee.babes
##
## REML criterion at convergence: -245.4
##
## Scaled residuals:
##     Min      1Q  Median      3Q     Max
## -2.8393 -0.4543  0.1591  0.5183  2.4582
##
## Random effects:
##  Groups   Name        Variance  Std.Dev.
##  MomTC    (Intercept) 0.0001734 0.01317
##  Residual             0.0007147 0.02673
## Number of obs: 61, groups:  MomTC, 14
##
## Fixed effects:
##              Estimate Std. Error        df t value Pr(>|t|)
## (Intercept)  0.403642   0.006801 11.054057  59.354 3.37e-15 ***
## MomTrmtGrpD -0.093928   0.010418 13.544105  -9.016 4.30e-07 ***
## ---
## Signif. codes:  0 '***' 0.001 '**' 0.01 '*' 0.05 '.' 0.1 ' ' 1
##
## Correlation of Fixed Effects:
##             (Intr)
## MomTrmtGrpD -0.653
```

```
plot(bmass.lmer)
```

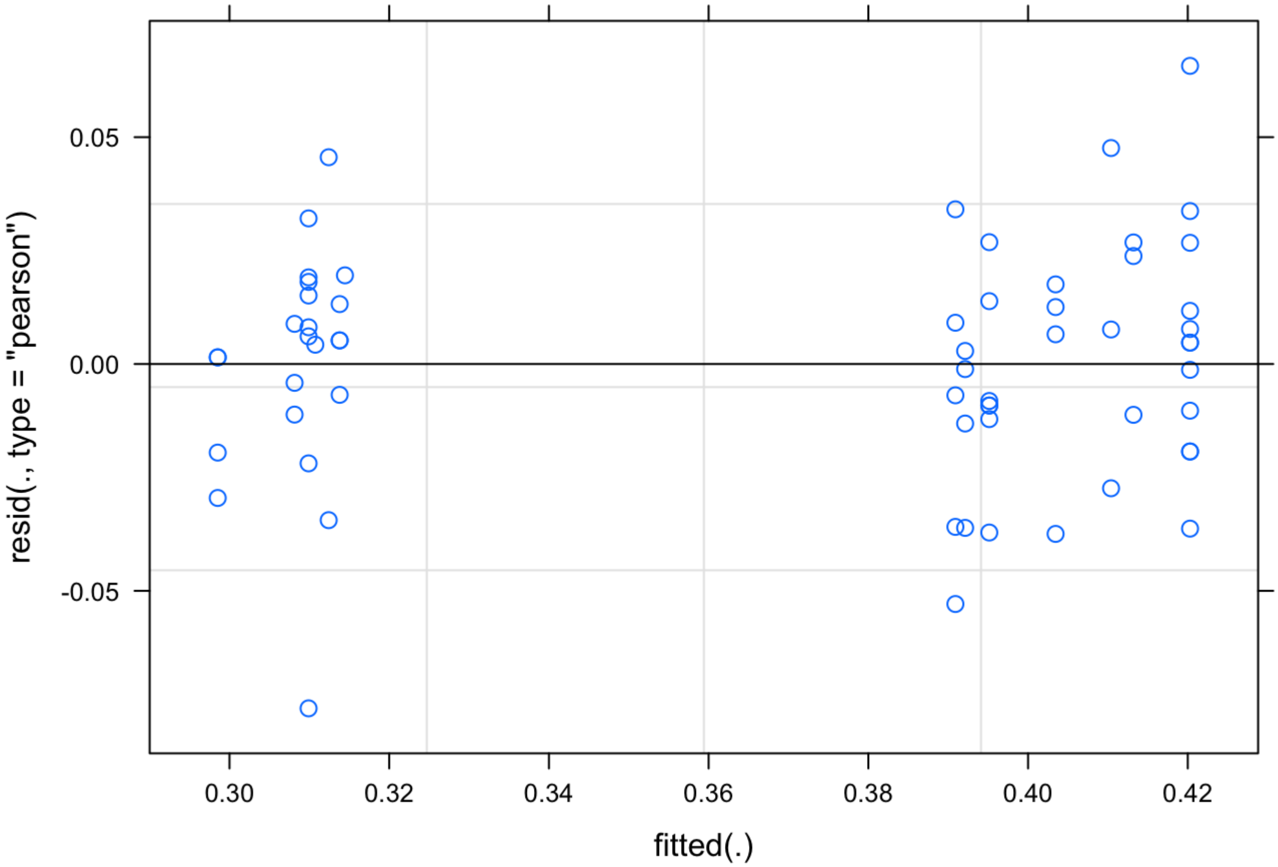


Snout-vent length

```
svl.lmer <- lmer(hatchSVL ~ MomTrmtGrp + (1|MomTC), data = ee.babes)
summary(svl.lmer)
```

```
## Linear mixed model fit by REML. t-tests use Satterthwaite's method [
## lmerModLmerTest]
## Formula: hatchSVL ~ MomTrmtGrp + (1 | MomTC)
##    Data: ee.babes
##
## REML criterion at convergence: 121.9
##
## Scaled residuals:
##     Min      1Q  Median      3Q     Max
## -2.5123 -0.5411  0.1526  0.6728  2.2176
##
## Random effects:
##  Groups   Name        Variance Std.Dev.
##  MomTC    (Intercept) 0.1695   0.4118
##  Residual             0.3325   0.5767
## Number of obs: 61, groups:  MomTC, 14
##
## Fixed effects:
##             Estimate Std. Error      df t value Pr(>|t|)
## (Intercept)  24.1306     0.1858  8.7973 129.862 9.50e-16 ***
## MomTrmtGrpD  -1.9917     0.2787 10.4514  -7.147 2.47e-05 ***
## ---
## Signif. codes:  0 '***' 0.001 '**' 0.01 '*' 0.05 '.' 0.1 ' ' 1
##
## Correlation of Fixed Effects:
##             (Intr)
## MomTrmtGrpD -0.667
```

```
plot(svl.lmer)
```

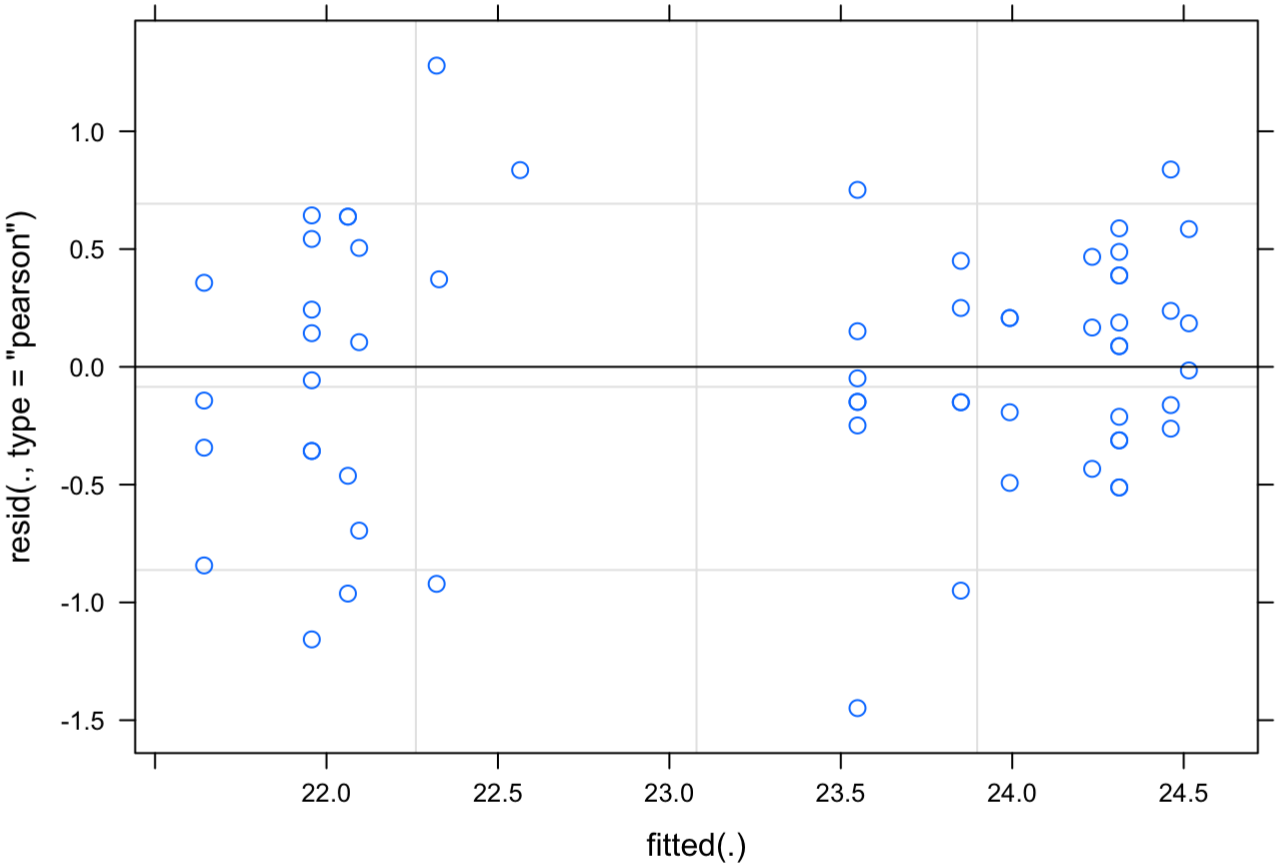
1.0
0.5
0.0
-0.5
-1.0
-1.5
resid(., type = "pearson")
22.0
22.5
23.0
23.5
24.0
24.5
fitted(.)
